# Double E─H Bond Activation of Ammonia and Water by Cyclic Gallaphosphene L(OCP)GaPGaL

**DOI:** 10.1002/anie.202525581

**Published:** 2026-01-16

**Authors:** Mahendra K. Sharma, Christoph Wölper, Gebhard Haberhauer, Stephan Schulz

**Affiliations:** ^1^ Institute of Inorganic Chemistry University of Duisburg‐Essen Universitätsstraße 5–7 Essen D‐45141; ^2^ Department of Chemistry Indian Institute of Technology Tirupati (IITTP) Venkatagiri Road, Yerpedu Post Tirupati District Andhra Pradesh 517619 India; ^3^ Institute of Organic Chemistry University of Duisburg‐Essen Universitätsstraße 5–7 Essen D‐45141; ^4^ Center for Nanointegration Duisburg‐Essen (CENIDE) University of Duisburg‐Essen Carl‐Benz‐Straße 199 Duisburg D‐47057

**Keywords:** Bond activation, Double bond, Gallium, Main group elements, Phosphorous

## Abstract

Cyclic gallaphosphene L(PCO)GaPGaL **1** (L = HC[C(Me)NAr]_2_; Ar = 2,6‐*i*‐Pr_2_‐C_6_H_3_) selectively reacts with NH_3_ and H_2_O at ambient temperature with twofold N─H and O─H bond activation and formation of compounds LGa(μ‐PH)(μ‐NHC(O)PH)GaL **2** and LGa(μ‐PH)(μ‐OC(O)PH)GaL **3**. Both nucleophilic P atoms of **1** are protonated in these reactions, while the electrophilic carbon atom of the bridging PCO unit binds to the remaining NH group / O atom. The formation of heterocycles **2** and **3**, which were characterized by heteronuclear NMR (^1^H, ^13^C{^1^H}, ^31^P{^1^H}) and IR spectroscopy, elemental analysis, and single‐crystal X‐ray diffraction (sc‐XRD), is only possible due to a beneficial interplay between the polar Ga─P double bond and the electrophilic nature of the C center in the bridging PCO unit. The reaction mechanism and energetics of the NH_3_ reaction was investigated in detail by quantum chemical calculations, which also highlight the importance of bimolecular reaction processes with the involvement of an additional NH_3_ molecule.

## Graphical abstract

Cyclic gallaphosphene **1** undergoes a twofold E─H bond activation with ammonia and water via a beneficial interplay between the polar “GaPGa” unit and the electrophilic nature of the C center in the bridging PCO unit, yielding six‐membered heterocycles **2** and **3**, in which both nucleophilic phosphorus atoms are protonated.

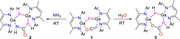

## Introduction

The direct utilization of ammonia (NH_3_) and water (H_2_O) in catalytic functionalization of E─H bonds (E = N or O) is a longstanding challenge in catalysis. NH_3_ is essential for the synthesis of pharmaceuticals, polymer additives, fertilizers, and industrial amines,^[^ ^1^, ^2^, ^3^, ^4^ ^]^ while the activation of O─H bonds in water is pivotal for advancing water‐splitting technologies, opening new possibilities for renewable hydrogen generation.^[^ ^4^ ^]^ However, functionalizing these molecules is challenging due to the strong N─H (BDE_N−H_ = 106.1 kcal mol^−^^1^; BDE = bond dissociation energy) and O─H bonds (BDE_O−H_ = 113.0 kcal mol^−^^1^).^[^ ^5^ ^]^ Moreover, the catalytic activation of both NH_3_ and water using metal complexes is often hindered by their tendency to form stable Werner‐type metal complexes, which diminishes the probability of successful E─H bond activation, or by their tendency to protonate basic and nucleophilic substituents of organometallic complexes.^[^ ^6^ ^]^ To overcome these challenges, modular strategies have been developed,^[^ ^1^, ^2^, ^3^, ^4^ ^]^ including the use of transition metal complexes with σ‐donating ligands,^[^ ^7^, ^8^, ^9^ ^]^ metal‐ligand cooperativity,^[^ ^10^, ^11^ ^]^ Lewis acid/base pairing,^[^ ^12^ ^]^ and aromatization/dearomatization sequences involving non‐innocent pincer‐type ligands,^[^ ^13^ ^]^ respectively.

**Scheme 1:**
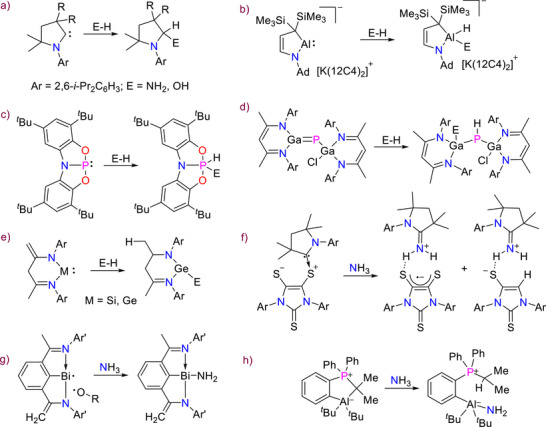
Key examples of different modes of single E‒H bond activation of ammonia and water. a–c) 1,1‐addition at a single carbon center, at a group 13 carbenoid, and at a geometrically constraint phosphorus atom; d) 1,2‐addition at a gallaphosphene; e) 1,4‐addition at the group 14 heterocarbenes; f) via SET and HAT at a cAAC‐dithiolene zwitterion; g) via radical pathway at a Bi(II) center; and (h) cooperativity at a FLP system.

The use of main group element compounds in small molecule activation reactions has also garnered increasing interest over the last two decades due to their ability to mimic transition metal reactivity in stoichiometric bond activation reactions as well as in catalytic reactions.^[^ ^14^, ^15^, ^16^, ^17^, ^18^, ^19^, ^20^, ^21^ ^]^ In 2007, Bertrand et al. reported the first NH_3_ activation by use of main group element compounds, namely cyclic (alkyl)(amino) carbenes (CAACs).^[^ ^22^ ^]^ Since this ground‐breaking report, stoichiometric E─H bond activation of NH_3_ and water were achieved with other carbenes,^[^ ^23^, ^24^ ^]^ heavier group 14 heterocarbenes and group 13 carbenoids,^[^ ^14^, ^15^, ^16^, ^17^, ^18^, ^19^, ^20^, ^21^, ^25^, ^26^, ^27^, ^28^, ^29^, ^30^, ^31^, ^32^, ^33^, ^34^ ^]^ multiple‐bonded main group element compounds,^[^ ^35^, ^36^, ^37^, ^38^, ^39^, ^40^, ^41^, ^42^ ^]^ geometrically constrained phosphines,^[^ ^43^, ^44^, ^45^, ^46^, ^47^, ^48^, ^49^, ^50^ ^]^ and frustrated Lewis pairs (FLPs),^[^ ^51^, ^52^, ^53^ ^]^ respectively, as depicted in Scheme 1.

Pörschke and co‐workers reported activation of a water O─H bond using a stannylene to get the Sn{CH(SiMe_3_)_2_}_2_(H)(OH) in 1998^[^ ^54^ ^]^ followed by numerous other researchers selectively cleaved one O─H bond of water using main group molecules. Remarkably, a dithiolene zwitterion complex^[^ ^55^ ^]^ and a Bi(II) compound^[^ ^56^ ^]^ were found to activate NH_3_ using SET/HAT and radical pathways, respectively (SET = single electron transfer; HAT = hydrogen atom transfer). Breher et al. recently reported an aluminum–carbon‐based ambiphile capable of activating the N─H bond of NH_3_ and subsequently catalytically transferring NH_3_ into a variety of organic substrates.^[^ ^57^ ^]^ Interestingly, all these reports have in common that only one single E─H bond of a single NH_3_ or water molecule was activated. Aldridge et al. recently demonstrated for the first time that also two NH_3_ molecules can be activated at a single metal center. The reaction of K[{(HCNAr)_2_BO}_2_Al] containing the strongly nucleophilic aluminyl anion [{(HCNAr)_2_BO}_2_Al]^−^ with an excess of NH_3_ occur with twofold single N─H bond activation to give the Al(III) bis(amide) K[{(HCNAr)_2_BO}_2_Al(NH_2_)_2_] as well as molecular H_2_.^[^ ^25^ ^]^ In marked contrast, the twofold E─H bond activation of only a single NH_3_ or water molecule is significantly more challenging and, to the best of our knowledge, has only observed rarely. Recently, Jana and Müller independently reported the double O─H bond activation reactions of water using a CAAC carbene and *N*‐heterocyclic‐carbene‐stabilized silylenes.^[^ ^58^, ^59^ ^]^ In addition, Schwarz et al. reported that the cationic aluminum oxo radical cluster [Al_2_O_3_]^·+^, generated by the collision‐induced dissociation of [Al_2_O_7_]^·+^, activates two N─H bonds of a single ammonia molecule.^[^ ^60^ ^]^

We recently reported on the synthesis of mono‐ and bisphosphaketenes LGa(X)PCO (X = Cl, PCO; L = HC[C(Me)N(Ar)]_2_; Ar = 2,6‐*i*‐Pr_2_C_6_H_3_) and their reactions with gallanediyl LGa to the corresponding gallaphosphenes LGa(X)PGaL (X = Cl, OCP) containing a Ga─P double bond.^[^ ^61^, ^62^ ^]^ The gallaphosphenes are valuable starting reagents for the activation of a variety of substrates. For instance, they reacted with heteroallenes to the corresponding cycloaddition products,^[^ ^63^ ^]^ were found to activate polar E─H bonds of small molecules at the Ga─P double bond,^[^ ^64^ ^]^ and reacted with N‐heterocyclic carbenes (NHCs) as phosphinidene transfer reagents,^[^ ^65^, ^66^ ^]^ respectively. In addition, the cyclic gallaphosphene LGa(OCP)PGaL **1** was found to isomerize at ambient temperature to give an unprecedented six‐membered metallacycle LGa(PCP)OGaL featuring a 1,3‐diphosphaallene unit, which reacted with singlet carbenes like an alkylidene carbene transfer reagent to the corresponding five‐membered metallaheterocycles featuring a unique 1,3‐diphospha‐1,3‐butadiene unit.^[^ ^67^ ^]^ These remarkable findings encouraged us to investigate the reactivity of the cyclic gallaphosphene LGa(OCP)PGaL **1** in more detail, and we herein report on its reactions with NH_3_ and water, which selectively occurred with double E─H (E = N, O) bond activation and formation of heterocycles **2** and **3**, respectively.^[^ ^62^ ^]^

## Results and Discussion

**Scheme 2:**
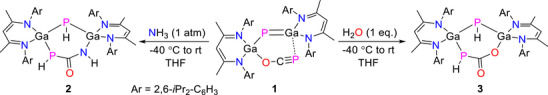
Reaction of gallaphosphene **1** with NH_3_ and H_2_O.

NH_3_ (1 atm) was added to a cooled (–40 °C) orange solution of **1** in THF and slowly warmed to ambient temperature, during which the color changed to colorless. LGa(μ‐PH)(μ‐NHC(O)PH)GaL **2** was isolated after workup of the solution as colorless solid in 59% yield (Scheme 2). This reaction also stands in remarkable contrast to the reaction with aniline (PhNH_2_), in which only a single N─H bond was activated at the Ga─P double bond.^[^ ^62^ ^]^ This difference may be attributed to the steric demand of the bulky phenyl group in aniline compared to the smaller hydrogen atoms in ammonia.

To prove if multifold E─H bond activation, as observed with NH_3_, can also be achieved with other p‐block element compounds, we reacted gallaphosphene **1** with water. Remarkably, the reaction occurred at mild reaction conditions with double O─H bond activation and formation of the six‐membered metallacycle LGa(μ‐PH)(μ‐OC(O)PH)GaL **3** as a colorless crystalline solid in 95% yield (Scheme 2).

Compounds **2** and **3** are stable for weeks under inert gas atmosphere at ambient temperature, but they rapidly decompose upon exposure to air and moisture. **2** and **3** show the expected doublets in the ^1^H NMR spectra (**2**: ‒1.14 ppm, ^1^ *J*_PH_ = 167.3 Hz; 3.85 ppm, ^1^ *J*_PH_ = 230.0 Hz, Figure S1; **3**: ‒0.60 ppm, ^1^ *J*_PH_ = 173.9 Hz; 0.39 ppm, ^1^ *J*_PH_ = 3.7 Hz, Figure S6) and in the proton‐coupled ^31^P NMR spectra for the P‒H units (**2**: ‒114.9 ppm, ^1^ *J*_PH_ = 230.7 Hz; ‒333.7 ppm, ^1^ *J*_PH_ = 167.3 Hz, Figure S4; **3**: ‒290.4 ppm, ^1^ *J*_PH_ = 173.6 Hz; ‒363.4 ppm, ^1^ *J*_PH_ = 5.0 Hz, Figure S9). The ^1^ *J*_PH_ coupling constants observed in the proton‐coupled ^31^P NMR spectra for the LGa(μ‐PH)GaL units in compounds **2** (167.3 Hz) and **3** (173.6 Hz) are similar to that reported for the 1,2‐addition product of aniline to gallaphosphene LGa(Cl)PGaL (−315.0 ppm, ^1^ *J*_PH_ = 177.0 Hz).^[^ ^64^ ^]^ The ^1^ *J*_PH_ coupling constant observed for the LGa[μ‐P(H)C(O)NH]GaL unit in compound **2** (^1^ *J*_PH_ = 230.0 Hz) is found larger than that of the LGa[μ‐P(H)C(O)O]GaL unit in compound **3** (^1^ *J*_PH_ = 5.0 Hz). This significant drop in the ^1^ *J*_PH_ coupling constant of **3** may be due to the more pronounced phosphine‐phospha‐enol tautomerism between the PH and adjacent CO group, which involves shifting the electronic structure from a typical P(III) pyramidal center toward a more P─C double‐bonded form and reduces the s‐character of the phosphorus atom thereby significantly reduces the ^1^ *J*_PH_ coupling constant.^[^ ^68^ ^]^ In addition, compound **2** shows a sharp doublet at 5.49 ppm (^3^ *J*_PH_ = 14.3 Hz) for the N−H group, which is shifted to a lower field compared to that of the LGa(PCO)PHGa(NHPh)L (2.77 ppm)^[^ ^62^ ^]^ and LGa(NH_2_)_2_ (−0.58 ppm),^[^ ^69^ ^]^ respectively, most likely due to the presence of the adjacent carbonyl group in **2**.

**Figure 1:**
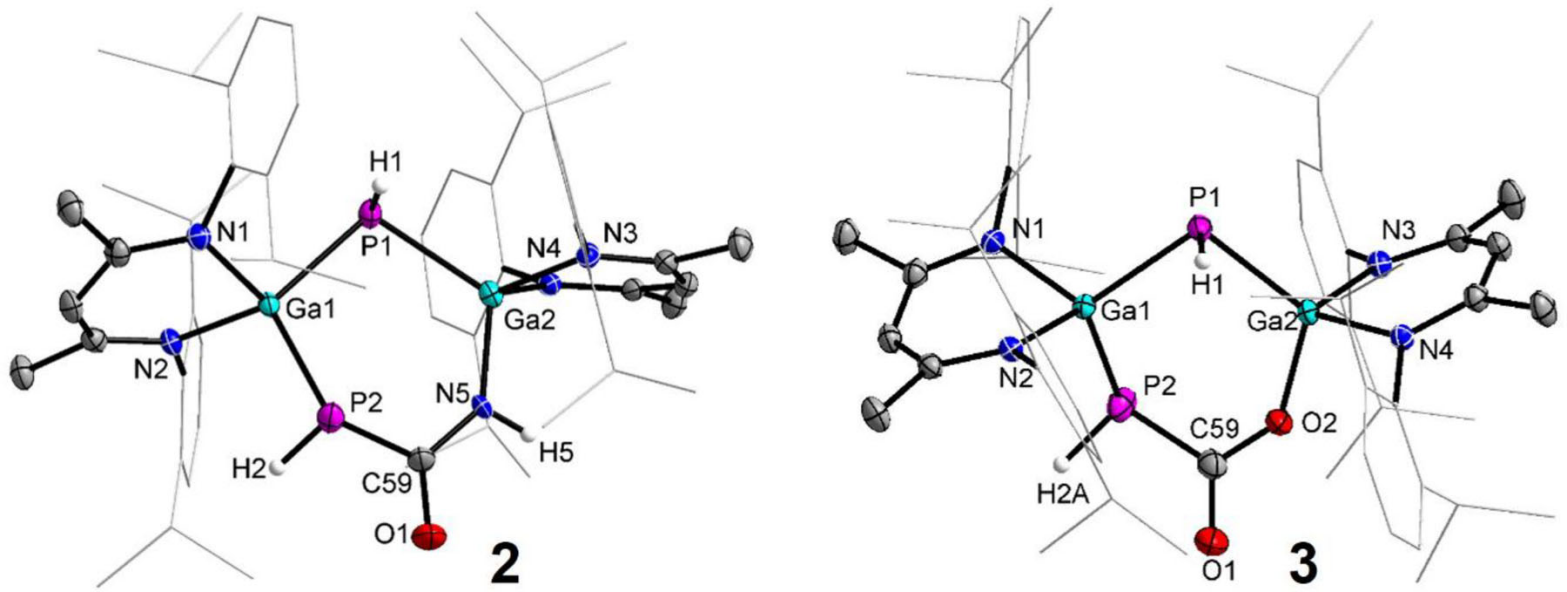
Molecular structures of compounds **2** and **3**. Thermal ellipsoids set at 50% probability; hydrogen atoms (except for those on nitrogen and phosphorus), solvent molecules (n‐hexane in **3**) and alternate positions of disordered parts omitted for clarity. Selected bond lengths (Å): **Compound 2**: Ga(1)˗P(1) 2.3185(9), Ga(1)˗P(2) 2.3715(18), P(2)˗C(59) 1.873(5), N(5)˗Ga(2) 1.847(5), P(1)˗Ga(2) 2.3180(6); **Compound 3**: Ga(1)‐P(1) 2.3367(4), Ga(1)‐P(2) 2.3589(5), Ga(2)‐O(2) 1.8668(11), Ga(2)‐P(1) 2.3248(4), P(2)‐C(59) 1.8524(17).

Single crystals of **2** and **3** suitable for X‐ray diffraction analysis were obtained by slow diffusion of *n*‐hexane into their saturated benzene/toluene solutions at room temperature (Figure 1).^[^ ^70^ ^]^ Compound **2** crystallizes in the monoclinic space group *Pn* and **3** in the triclinic space group *P*‐1. In both **2** and **3**, the Ga atoms within the central six‐membered metallacycle adopt fourfold‐coordinated distorted tetrahedral geometries, whereas the P atoms adopt trigonal pyramidal geometries. The Ga─P─Ga bond angles in **2** (111.26(4)°) and **3** (112.365(15)°) are similar to those in the acyclic compounds LGa(X)PHGa(NHPh)L (X = Cl, 111.07(18)° and PCO, 113.14(2)°).^[^ ^62^, ^64^ ^]^ Likewise, the Ga─P bond lengths in **2** (Ga1‒P1 2.3185(9) Å, Ga1‒P2 2.3715(18) Å, Ga2‒P1 2.3180(6) Å) and **3** (Ga1‐P1 2.3367(4) Å, Ga1‐P2 2.3589(5) Å, Ga2‒P1 2.3248(4) Å) are almost equidistant and agree with the sum of the calculated Ga‒P single‐bond radii (Ga 1.24 Å; P 1.11 Å)^[^ ^71^ ^]^ as well as with Ga─P single bonds observed in diphosphene [L(Cl)GaP]_2_ (2.313(3) Å),^[^ ^66^ ^]^ and in 1,2‐diphospha‐1,3‐butadiene (2.2844(4) Å).^[^ ^72^ ^]^ The Ga2‒N5 bond length in **2** (1.847(5) Å) is slightly shorter than that of the LGa(X)PHGa(NHPh)L (X = Cl, 1.8762(14) Å and PCO, 1.8746(18) Å),^[^ ^62^, ^64^ ^]^ which is consistent with the sterically less bulky N–H group in **2**.

DFT calculations were performed to gain deeper insight into the energetics of these reactions using the program packages Gaussian 16.^[^ ^73^ ^]^ The geometric parameters of the stationary points were optimized using the density functional methods PBE0^[^ ^74^ ^]^ with the empirical dispersion D3BJ.^[^ ^75^ ^]^ Def2‐SVP^[^ ^76^ ^]^ was used as the basis set. The energies of the stationary points were calculated using the density functionals PBE0‐D3BJ and the basis set def2‐TZVP.^[^ ^76^ ^]^ To account for solvent effects, the solvent model SMD^[^ ^77^ ^]^ (THF as solvent) was employed for the single‐point calculations. A well‐known problem in the calculation of Gibbs energies is the overestimation of the calculated entropies,^[^ ^78^, ^79^ ^]^ which are particularly significant in bi‐ and trimolecular reactions. To obtain a better comparison with experimental values, the entropy in solution is often set at 50%–70% of the calculated entropy in the gas phase for bimolecular reactions.^[^ ^80^, ^81^, ^82^ ^]^ Since we have found good agreement between calculations and experiments with a value of 70% so far,^[^ ^83^ ^]^ we have also used the G_70%_ values here.

**Figure 2:**
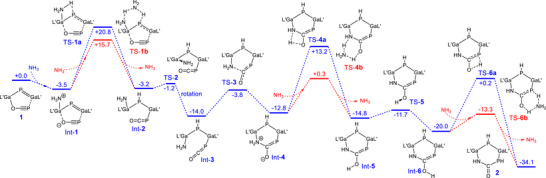
Gibbs energies (G70%) for the reaction gallaphosphene **1** with NH_3_ calculated by means of PBE0‐D3BJ/def2‐TZVP(SMD, THF)//PBE0‐D3BJ/def2‐SVP. L′ = HC[C(Me)NAr]_2_ and Ar = 2,6‐*i*‐Pr_2_C_6_H_3_. Herein G70% means that 70% calculated gas‐phase entropy contributes to solution‐phase free energy. The values are given in kcal mol^−1^.

Figure 2 presents the Gibbs energies for the reaction of gallaphosphene **1** and NH_3_. The reaction is started by a nucleophilic attack of NH_3_ on the gallium center, to which the oxygen atom of the PCO unit is bound, forming the adduct Int‐**1**. This finding is in remarkable contrast to previous reactions of gallaphosphene LGaPGa(Cl)L with NH_3_, H_2_O as well as primary amines RNH_2_, alcohols ROH, thio‐, and selenophenols,^[^ ^64^ ^]^ respectively, in which the electrophilic Ga atom of the Ga─P double bond is attacked by the nucleophilic substrate. The attack at the gallium atom of the Ga─P double bond of **1** as well as at the electrophilic carbon atom of the PCO unit leads to significantly higher‐energy intermediates (see Figure S18), which is why these pathways can be ruled out. The next step is tautomerization: For this reaction, as well as for the two other tautomerization reactions, we calculated on the one hand the *intramolecular* migration of the hydrogen atom (blue pathway) and, on the other hand, a *bimolecular* reaction in which an additional NH_3_ molecule catalyzes the hydrogen transfer via a six‐membered transition state (red pathway). In the first tautomerization (step from Int‐**1** to Int‐**2**), the bimolecular pathway with an activation barrier of +19.2 kcal mol^−1^ is significantly more favorable than the intramolecular pathway (24.3 kcal mol^−1^). Int‐**2** stabilizes by rotation to the energetically more favored (by 10.8 kcal mol^−1^) Int‐**3**. Unfortunately, neither the adduct Int‐**1** nor the subsequently formed N─H bond activation product L(H_2_N)Ga(μ‐PH)(PCO)GaL Int‐**3** were experimentally observed by variable‐temperature (VT) ^1^H and ^31^P NMR spectroscopy. The next reaction occurs when the amino group in Int‐**3** attacks the electrophilic carbon atom of the PCO unit intramolecularly, forming the zwitterion Int‐**4**. The activation barrier for this step amounts to 10.2 kcal mol^−1^. Stabilization can be achieved by a second tautomerization step leading to Int‐**5**. This can again proceed intramolecularly (blue pathway) or bimolecularly (red pathway) and the energy difference again is enormous (roughly 13 kcal mol^−1^). A rotation around the C─O bond with only a small barrier (approx. 3 kcal mol^−1^) results in Int‐**6**, which can be converted to product **2** through tautomerization. Here, too, there is an energy difference of 13 kcal mol^−1^ between the intramolecular and bimolecular processes. To summarize, the quantum chemical calculations prove that both phosphorus atoms are protonated via tautomerization reactions in which a further NH_3_ molecule acts as a catalyst. The formation of **2** from **1** and NH_3_ is strongly exergonic, with a Δ*G*° of ‐34.1 kcal mol^−1^. For the formation of **3** from **1** and H_2_O, a similar value of ‐34.8 kcal mol^−1^ is calculated, and the reaction pathway is expected to proceed similarly. An analysis of the frontier orbitals of **2** and **3** reveals that the HOMO and LUMO are similar in both compounds (Figures S16 and S17). While the HOMO essentially consists of a linear combination of the free electron pairs at the phosphorus atoms, the LUMO is localized at the β‐diketiminate ligand. The natural charges of the atoms in the central seven‐membered ring also exhibit comparable values.

## Conclusion

To conclude, we report for the first time on double E─H bond activation of single ammonia and water molecules in reactions with cyclic gallaphosphene L(OCP)GaPGaL **1** under very mild reaction conditions. While acyclic gallaphosphene L(Cl)GaPGaL was previously reported to react with polar E─H bonds with single E─H bond activation at the P─Ga double bond, the double E─H bond activation leading to heterocycles **2** and **3** is only possible due to a beneficial interplay between the polar “GaPGa” unit and the electrophilic nature of the C center in the bridging PCO unit. The electropositive Ga center is initially attacked by NH_3_ / H_2_O followed by the first E─H bond cleavage and protonation of the nucleophilic P atom, while the second E─H bond cleavage occurs after coordination of the NH_2_/OH groups to the electrophilic carbon atom of the bridging PCO. As formed heterocycles **2** and **3** were fully characterized including sc‐XRD. In addition, DFT calculations provided insight into the energetics of both reactions. The NH_3_ reaction mechanism was calculated in detail, and bimolecular reaction pathways were identified as most likely mechanism, in which one additional NH_3_/H_2_O molecule facilitates both proton transfer reactions to the nucleophilic P atoms. Large energy difference between the intramolecular and bimolecular NH_3_ activation of 5.1 (NH_3_) kcal mol^−1^ for the first N─H bond activation and of 12.9 (NH_3_) for the second N─H bond activation reaction were calculated. This study highlights the promising potential of multiply bonded gallaphosphenes featuring a polarized double bond in multifold bond activation reactions.

## Supporting Information

The authors have cited additional references within the supporting information.^[^ ^84^, ^85^, ^86^, ^87^ ^]^

## Conflict of Interests

The authors declare no conflict of interest.

## Supporting information



Supporting Information

## Data Availability

The data that support the findings of this study are available in the supporting information of this article.
