# Supplementary material for: Double E─H Bond Activation of Ammonia and Water by Cyclic Gallaphosphene L(OCP)GaPGaL
Source: Angew Chem Int Ed Engl. 2026 Jan 16;65(8):e25581. doi: 10.1002/anie.202525581 (PMC12910143; doi:10.1002/anie.202525581)
Supplement: Supplementary file 1 — Supporting Information [file ANIE-65-e25581-s001.pdf]

Supporting Information

Content

1. Experimental Section S3-5

Synthesis of LGa(OCPH)PH(NH)LGa (**2**) and LGa(PH)PH(CO_2)LGa (**3**)

2. Spectroscopic Characterization S6-11

Figure S1-S5. ^{1}H, ^{13}C, ^{31}P NMR and IR spectra of **2**.

Figure S6. IR spectrum of compound **2** simulated by means of PBE0-D3BJ/def2-SVP.

Figure S7-S11. ^{1}H, ^{13}C, ^{31}P NMR and IR spectra of **3**.

Figure S12. IR spectrum of compound **3** simulated by means of PBE0-D3BJ/def2-SVP.

3. Crystallographic Details S12-15

Table S1. Crystal data and structure refinement parameters of **2**, and **3**.

Figure S13, S14. Molecular structures of **2**, and **3**.

4. Computational Calculations S16-68

Figure S15. Gibbs energies ($G_{70\%}$) for the nucleophile attack of NH_3 at gallaphosphene **1** calculated by means of PBE0-D3BJ/def2-TZVP(SMD,THF)//PBE0-D3BJ/def2-SVP.

Figure S16. HOMO (left) and LUMO (right) of **2** calculated by means of PBE0-D3BJ/def2-TZVP(SMD,THF)//PBE0-D3BJ/def2-SVP.

Figure S17. HOMO (left) and LUMO (right) of **3** calculated by means of PBE0-D3BJ/def2-TZVP(SMD,THF)//PBE0-D3BJ/def2-SVP.

Figure S18. Natural charges from the NBO analysis of **2** and **3** calculated by means of PBE0-D3BJ/def2-TZVP(SMD,THF)//PBE0-D3BJ/def2-SVP.

Table S2. Absolute energies of the calculated compounds by means of different methods.

5. References S69-70

1. Experimental Section

General procedure. All experiments and manipulations were carried out under dry argon atmosphere using either standard Schlenk or glovebox techniques. Toluene, *n*-hexane and dichloromethane were dried using an MBraun solvent drying system (SPS), degassed and stored over molecular sieve (4 Å). THF was dried by refluxing over Na/K alloy, distilled under argon atmosphere, degassed and stored over molecular sieve (4 Å). Deuterated solvents (THF-d_8, and C_6D_6) were dried by refluxing over Na/K alloy, distilled prior to use and stored over molecular sieve (4 Å). NH_3 gas was purchased from Air Liquide and Co. and dried over potassium metal prior to use. Distilled water (H_2O) was degassed twice using Freeze-Pump-Thaw method and used for the preparation of a 1M solution in THF. THF·BH_3 was purchased from Sigma Aldrich and used as received. L(PCO)GaPGaL **1** (L = HC[C(Me)N(Dipp)]$_2$, Dipp = 2,6-*i*-Pr$_2$-C_6H_3) was prepared by literature method.[1] NMR spectra (δ in ppm) were recorded using a Bruker Avance II 400 (^{1}H 400 MHz, ^{13}C{^{1}H} 100 MHz, ^{31}P{^{1}H} 161 MHz) spectrometer and were referenced to internal C_6D_5H (^{1}H δ = 7.16; ^{13}C δ = 128.06). ^{31}P NMR spectra were referenced to internal C_6D_5H (^{1}H δ = 7.16) using chi-values (χ).[2] Elemental analyses were performed at the *Elementaranalyse Labor* der Universität Duisburg-Essen. IR spectra were recorded in a glovebox with a Bruker ALPHA-T FT-IR spectrometer equipped with a single-reflection ATR sampling module. Melting points were measured in sealed glass capillaries.

Compound **1** exist in two equilibrium states in solution: one form corresponds to the phosphaethynolato isomers, L(PCO)GaPGaL (left side, Scheme 1), while the second is represented by the corresponding phosphaketenyl isomers, LGaPGa(PCO)L (right side, Scheme S1) according to DFT calculations.[3]

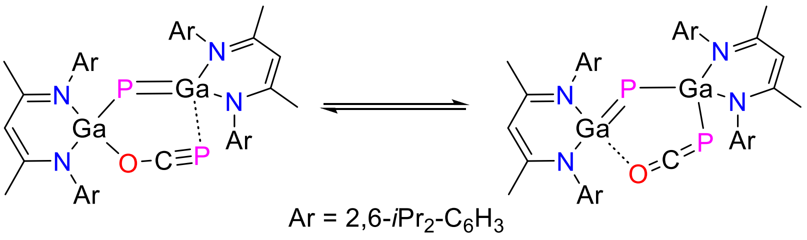

Scheme S1. Equilibrium states of **1** in solution.

Synthesis of LGa(OCPH)PH(NH)LGa (2).

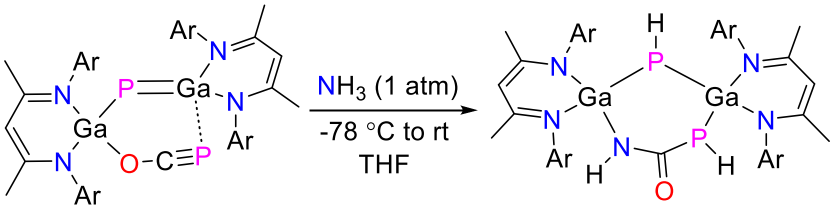

1 Ar = 2,6-*i*Pr$_2$-C$_6$H$_3$ 2

NH_3 (1 bar) was added to a cooled THF solution (5 mL) of **1** (200 mg, 0.19 mmol) at -78 ˚C and slowly brought to ambient temperature and stirred for 10 minutes. All volatiles were removed in vacuo to yield a colorless crystalline solid, which was washed with *n*-hexane (1 mL) and dried. Yield: 59% (120 mg). Single crystals suitable for sc-XRD were grown by diffusing *n*-hexane into a saturated toluene solution of **2** at room temperature. M.p. 161 °C (dec.). Anal. calcd. (%) for $C_{59}H_{85}Ga_2N_5OP_2$ (1079.47): C, 65.51; H, 7.92; N, 6.47. Found: C, 65.61; H, 8.03; N, 6.61. **^{1}H NMR** (500 MHz, C_6D_6, 298 K) δ = –1.14 (d, $^1J_{PH}$ = 167.3 Hz, 1H, P*H*), 0.95 (d, $^3J_{HH}$ = 6.8 Hz, 6H, CH(C*H*$_3$)$_2$), 0.98 (d, $^3J_{HH}$ = 6.8 Hz, 6H, CH(C*H*$_3$)$_2$), 1.11-1.14 (m, 18H, CH(C*H*$_3$)$_2$), 1.18 (d, $^3J_{HH}$ = 6.9 Hz, 6H, CH(C*H*$_3$)$_2$), 1.24 (d, $^3J_{HH}$ = 6.8 Hz, 6H, CH(C*H*$_3$)$_2$), 1.28 (d, $^3J_{HH}$ = 6.8 Hz, 6H, CH(C*H*$_3$)$_2$), 1.37 (s, 6H, CC*H*$_3$), 1.43 (s, 6H, CC*H*$_3$), 2.90 (sept, $^3J_{HH}$ = 6.8 Hz, 2H, C*H*(CH$_3$)$_2$), 3.19 (sept, $^3J_{HH}$ = 6.8 Hz, 4H, C*H*(CH$_3$)$_2$), 3.36 (sept, $^3J_{HH}$ = 6.8 Hz, 2H, C*H*(CH$_3$)$_2$), 3.85 (d, $^1J_{PH}$ = 230.0 Hz, 1H, P*H*), 4.52 (s, 1H, C*H*), 4.57 (s, 1H, C*H*), 5.49 (d, $^3J_{PH}$ = 14.3 Hz, 1H, N*H*), 6.94–6.98 (m, 4H, C$_6$*H*$_3$), 6.99–7.02 (m, 4H, C$_6$*H*$_3$), 7.11–7.14 (m, 4H, C$_6$*H*$_3$). **^{13}C{^{1}H} NMR** (126 MHz, C_6D_6, 298 K) δ = 23.6, 23.9, 24.1, 24.4, 24.6, 25.1, 25.4 (CH(*C*H$_3$)$_2$), 27.0, 28.3, 28.8, 29.6 (*C*H(CH$_3$)$_2$), 96.3, 96.6 (*C*H), 124.1, 125.5, 125.6, 127.0, 127.2, 141.5, 141.9, 142.5, 142.9, 145.0, 145.6 (*C*$_6$H$_3$), 168.6, 168.9 (*C*$_6$H$_3$), 183.1 (d, $^1J_{PC}$ = 17.6, *C*O). **^{31}P{^{1}H} NMR** (202 MHz, C_6D_6, 298 K) δ = –114.9 (d, $^3J_{PP}$ = 7.3, P*P*), –333.7 (br, *P*P). **^{31}P NMR** (202 MHz, C_6D_6, 298 K) δ = –114.9 (ddd, J_{PH} = 230.7, 14.5, 7.27, P*H*), –333.7 (dd, J_{PH} = 167.3, P*H*). **ATR-IR**: *v* 3060, 2957, 2922, 2862, 2258 (PH), 1898 (CO), 1524, 1433, 1383, 1315, 1257, 1174, 1056, 1019, 936, 858, 794, 757, 705, 612, 529 cm^{-1}.

Synthesis of LGa(PH)PH(CO$_2$)LGa (3).

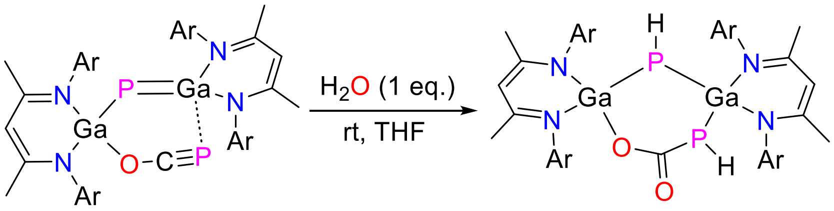

1 Ar = 2,6-*i*Pr$_2$-C$_6$H$_3$ 3

H_2O (19 µL, 0.19 mmol, 1M in THF) was added to a THF solution (5 mL) of **1** (200 mg, 0.19 mmol) at ambient temperature and stirred for 5 minutes. All volatiles were then removed in vacuo to yield a colorless crystalline solid, which was washed with *n*-hexane (1 mL) and dried. Yield: 95% (193 mg). Single crystals suitable for sc-XRD were grown by cooling a saturated *n*-hexane solution of **3** at –30 °C for overnight. M.p. 159 °C (dec.). Anal. calcd. (%) for $C_{59}H_{84}Ga_2N_4O_2P_2$ (1080.46): C, 65.45; H, 7.82; N, 5.17. Found: C, 65.49; H, 7.87; N, 5.21. **^{1}H NMR** (400 MHz, C_6D_6, 298 K) δ = –0.60 (d, $^1J_{PH}$ = 173.9 Hz, 1H, P*H*), 0.39 (d, $^1J_{PH}$

= 3.7 Hz, 1H, P*H*), 0.97 (d, $^{3}J_{HH}$ = 6.7 Hz, 6H, CH(C*H*$_3$)$_2$), 1.01 (d, $^{3}J_{HH}$ = 6.8 Hz, 6H, CH(C*H*$_3$)$_2$), 1.12 (d, $^{3}J_{HH}$ = 6.8 Hz, 12H, CH(C*H*$_3$)$_2$), 1.18 (pst, $^{3}J_{HH}$ = 7.7 Hz, 18H, CH(C*H*$_3$)$_2$), 1.28 (d, $^{3}J_{HH}$ = 6.7 Hz, 6H, CH(C*H*$_3$)$_2$), 1.45 (s, 6H, CC*H*$_3$), 1.49 (s, 6H, CC*H*$_3$), 3.09 (sept, $^{3}J_{HH}$ = 6.8 Hz, 2H, C*H*(CH$_3$)$_2$), 3.18 (sept, $^{3}J_{HH}$ = 6.8 Hz, 2H, C*H*(CH$_3$)$_2$), 3.54 (sept, $^{3}J_{HH}$ = 6.8 Hz, 2H, C*H*(CH$_3$)$_2$), 3.73 (sept, $^{3}J_{HH}$ = 6.8 Hz, 2H, C*H*(CH$_3$)$_2$), 4.73 (s, 1H, C*H*), 4.77 (s, 1H, C*H*), 6.97 (t, $^{3}J_{HH}$ = 6.8 Hz, 4H, C_6*H*$_5$), 7.07 (t, $^{3}J_{HH}$ = 6.7 Hz, 4H, C_6*H*$_3$), 7.12-7.16 (m, 4H, C_6*H*$_5$). **^{13}C{^{1}H} NMR** (101 MHz, C_6D_6, 298 K) δ = 24.0, 24.2, 24.3, 24.4, 24.7, 25.1, 25.8 (CH(*C*H$_3$)$_2$), 27.4, 27.5, 27.6, 28.1, 29.1, 29.2, 29.5 (*C*H(CH$_3$)$_2$), 96.5, 99.8 (*C*H), 124.0, 124.3, 125.1, 125.5, 127.2, 127.4, 141.3, 141.8, 142.6, 143.1, 145.2, 145.7 (*C*$_6$H$_3$, *C*$_6$H$_5$), 168.5, 170.0 (*C*$_6$H$_3$), 186.1 (d, $^{1}J_{PC}$ = 90.9 Hz, *C*O). **^{31}P{^{1}H} NMR** (162 MHz, C_6D_6, 298 K) δ = –290.4 (d, J_{PP} = 5.0 Hz, P*P*), –363.4 (d, J_{PP} = 5.0 Hz, *P*P). **^{31}P NMR** (162 MHz, C_6D_6, 298 K) δ = –290.4 (dd, J_{PH} = 173.6, 5.0 Hz, P*H*), –363.4 (br, P*H*). **ATR-IR**: *ν* 2957, 2924, 2865, 2261 (PH), 1902 (CO), 1528, 1460, 1437, 1383, 1315, 1257, 1176, 1100, 1056, 1019, 939, 862, 796, 754, 616, 564, 527 cm^{-1}.

2. Spectroscopic Characterization

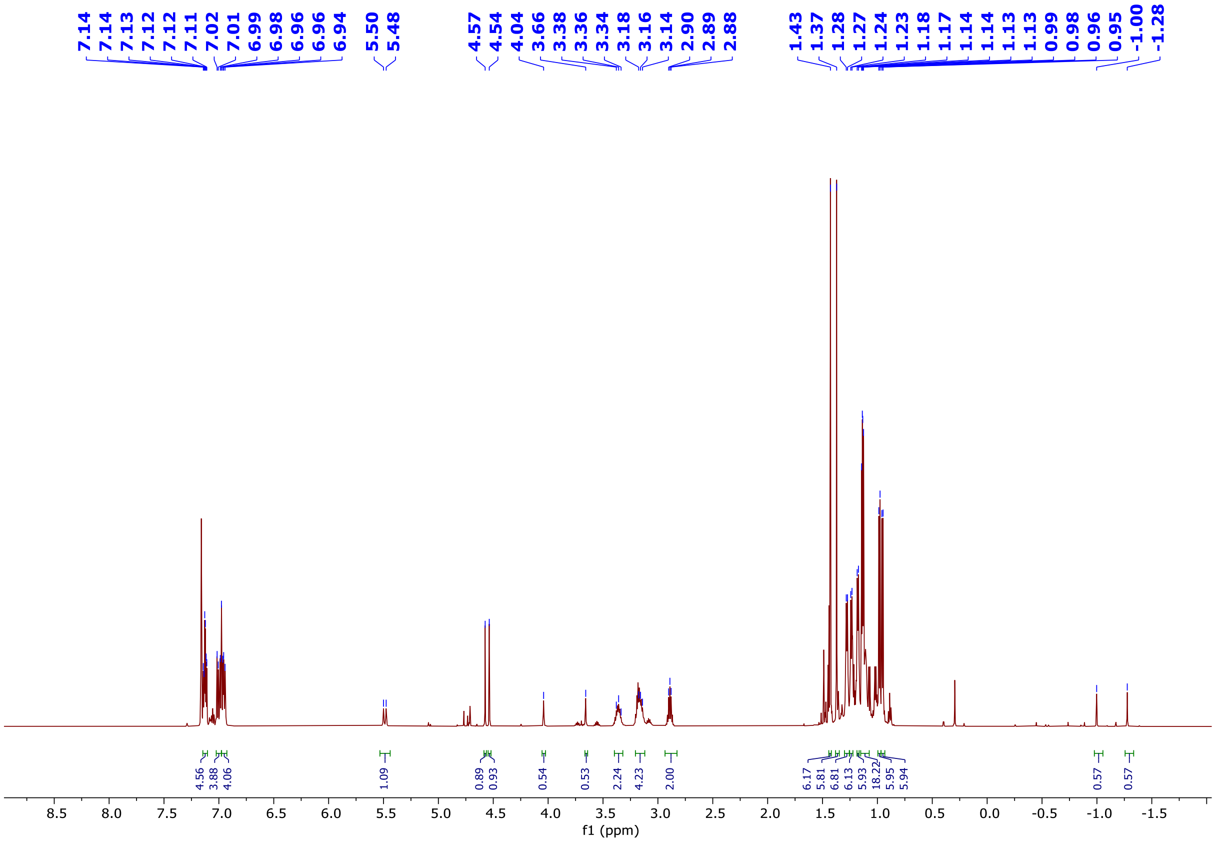

Figure S1. ^{1}H NMR (400 MHz, C_6D_6, 298 K) spectrum of compound **2**.

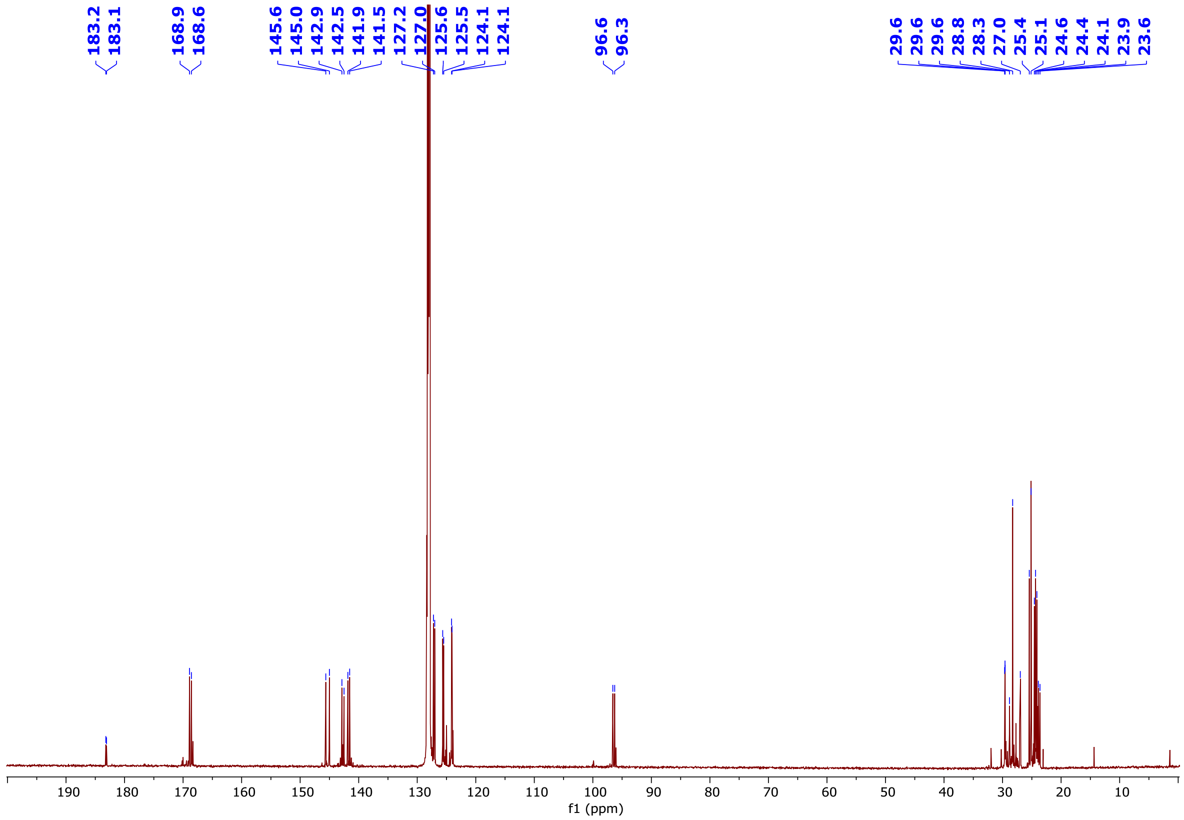

Figure S2. $^{13}C\{^{1}H\}$ NMR (101 MHz, C_6D_6, 298 K) spectrum of compound **2**.

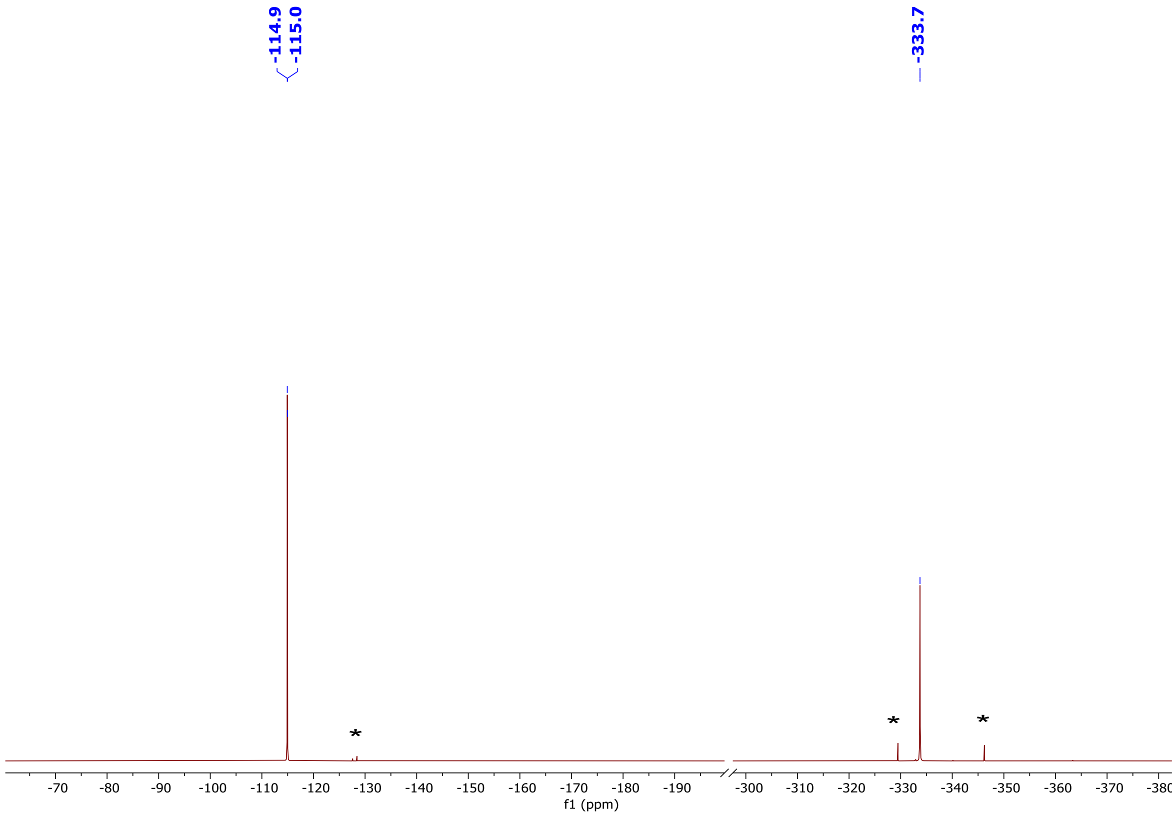

Figure S3. $^{31}P\{^{1}H\}$ NMR (162 MHz, C_6D_6, 298 K) spectrum of compound **2**. *Unidentified minor impurities.

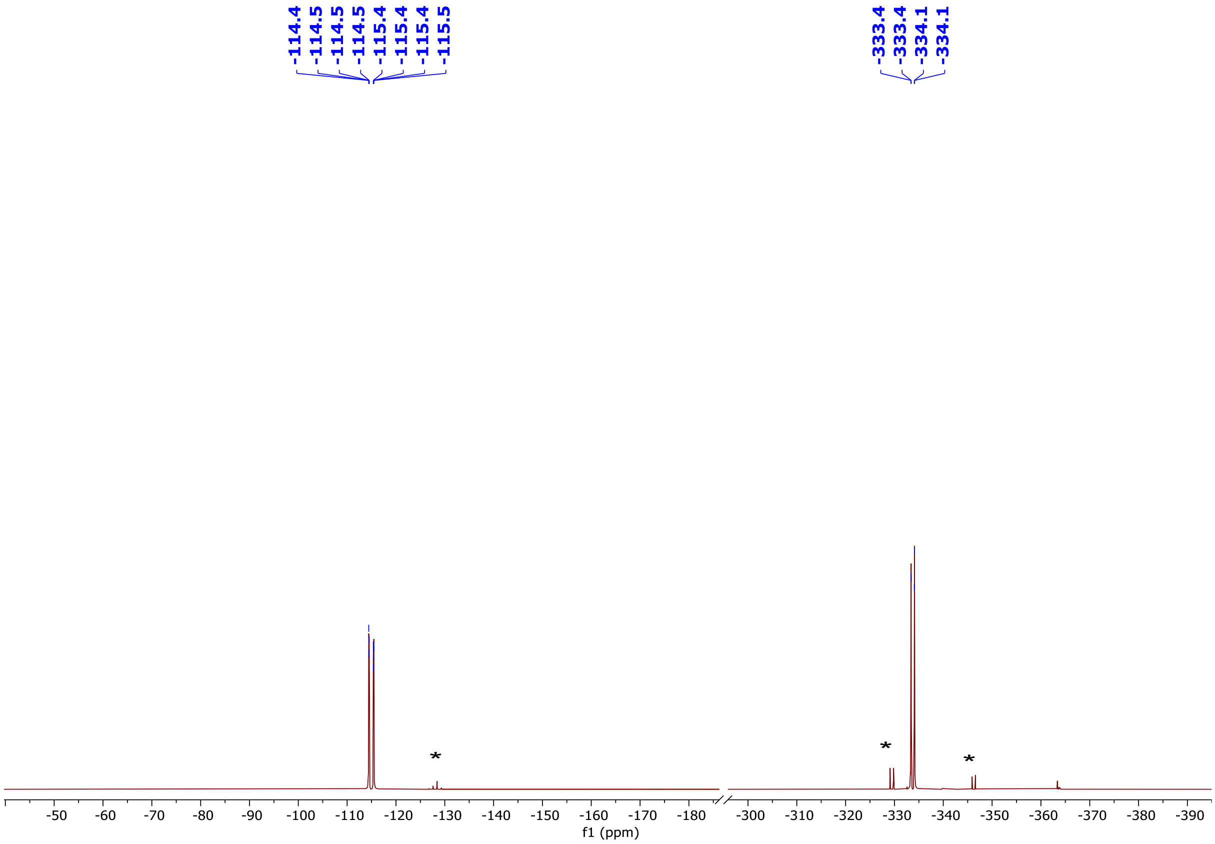

Figure S4. ^{31}P NMR (162 MHz, C_6D_6, 298 K) spectrum of compound **2**. *Unidentified minor impurities.

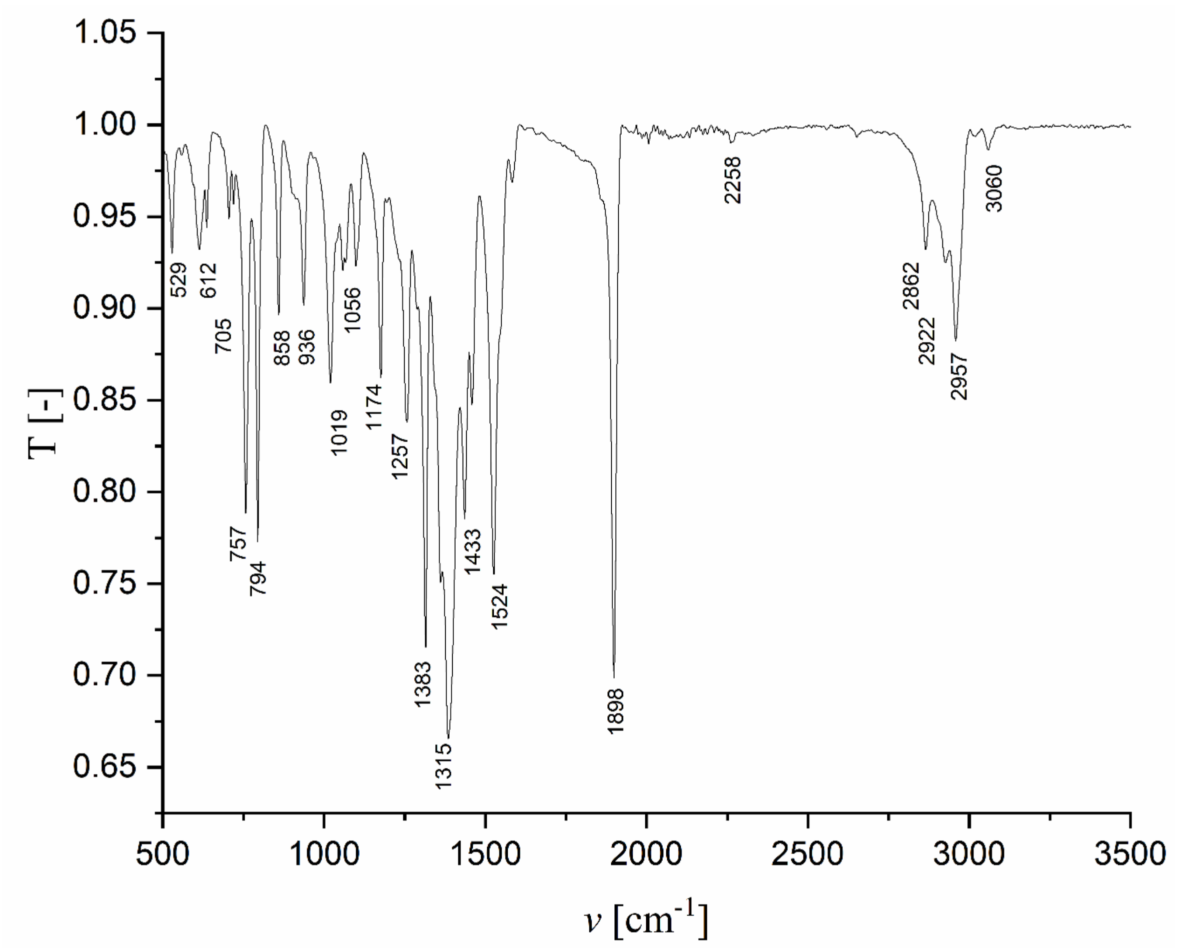

Figure S5. ATR-IR spectrum of **2**.

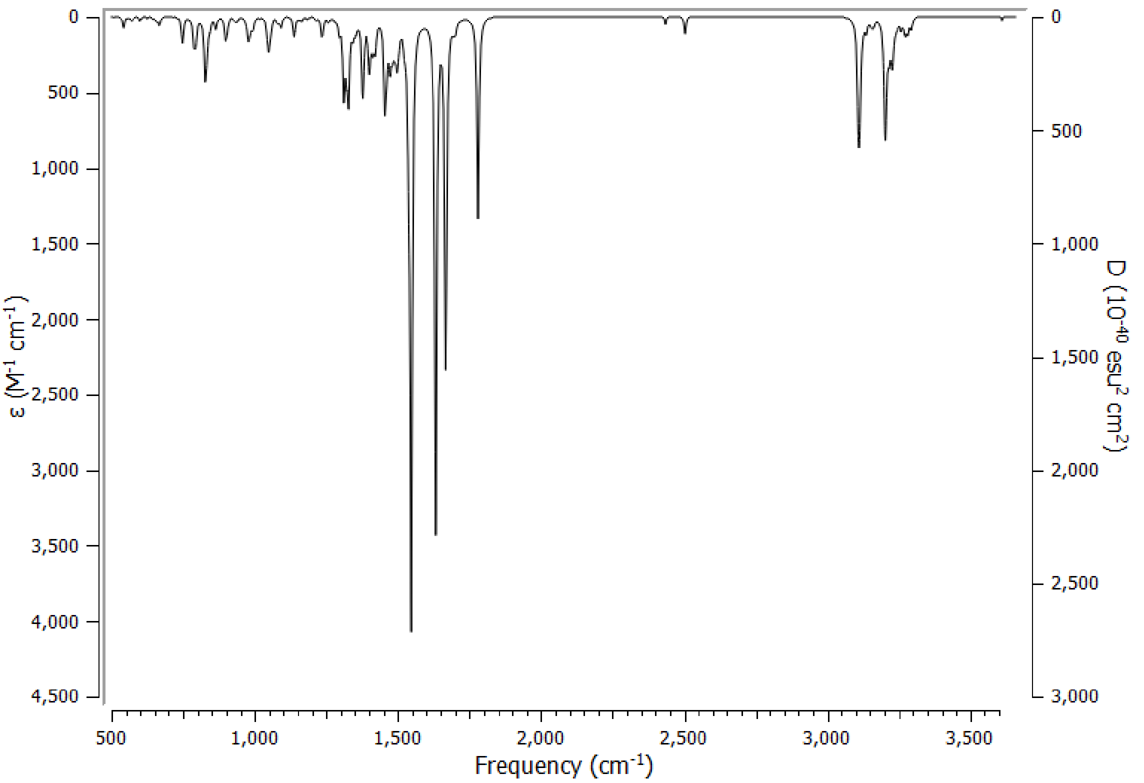

Figure S6. IR spectrum of compound **2** simulated by means of PBE0-D3BJ/def2-SVP. Selected stretching frequencies: C=O 1752 cm^{-1}, P-H 2394 cm^{-1} and 2460 cm^{-1}, N-H 3544 cm^{-1}.

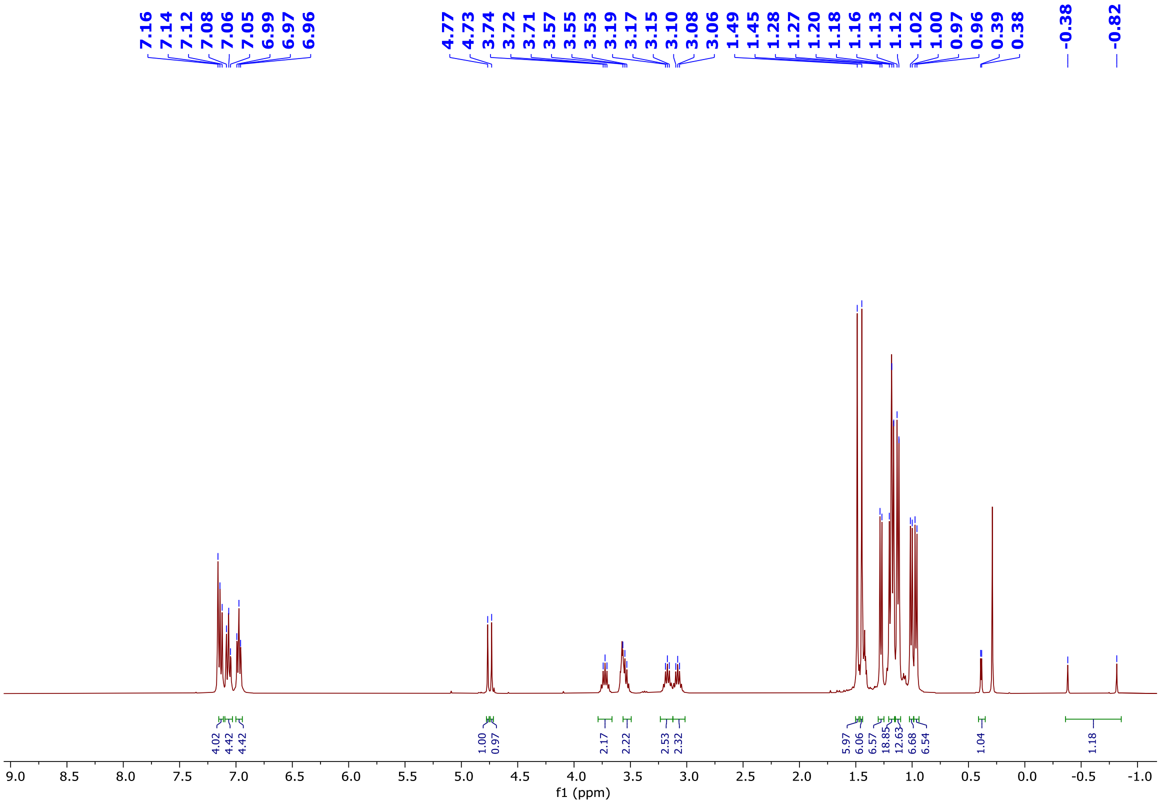

Figure S7. ^{1}H NMR (400 MHz, C_6D_6, 298 K) spectrum of compound **3**.

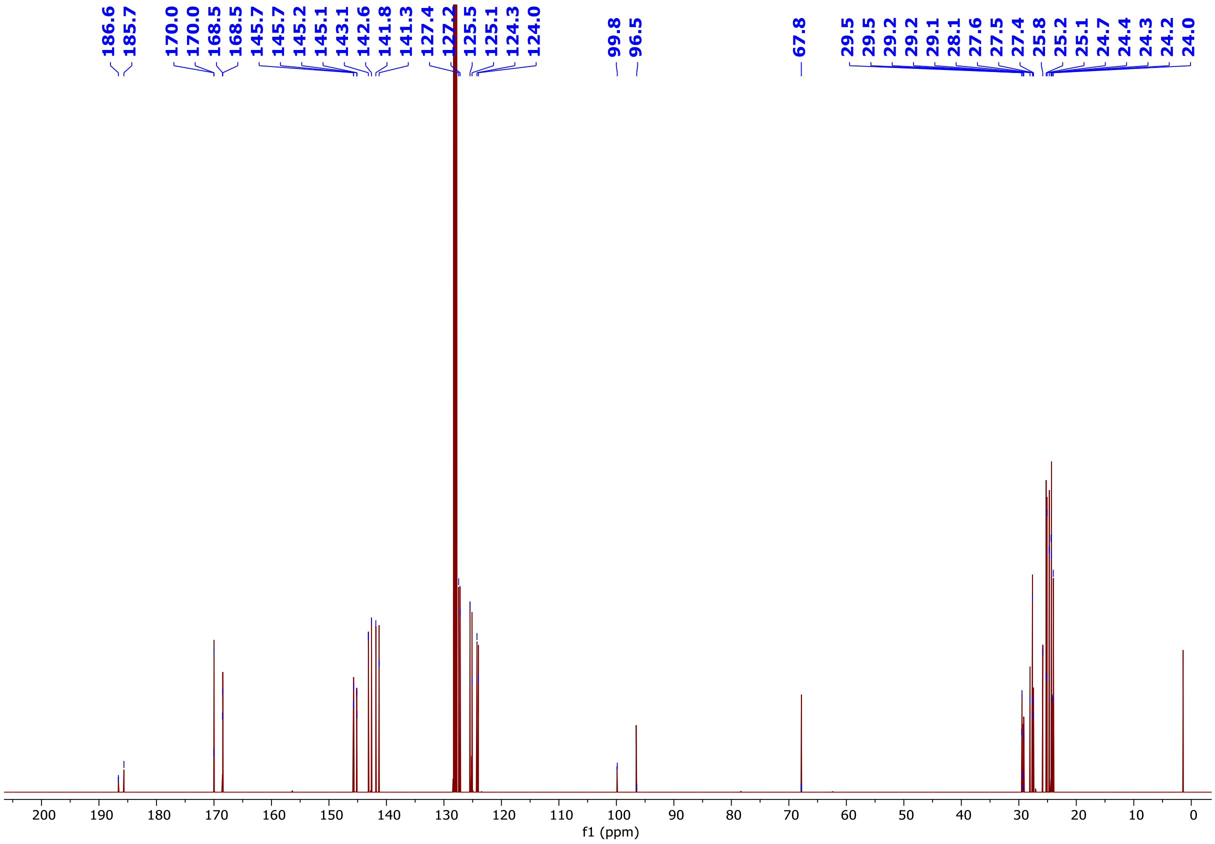

Figure S8. $^{13}C\{^1H\}$ NMR (101 MHz, C_6D_6, 298 K) spectrum of compound **3**.

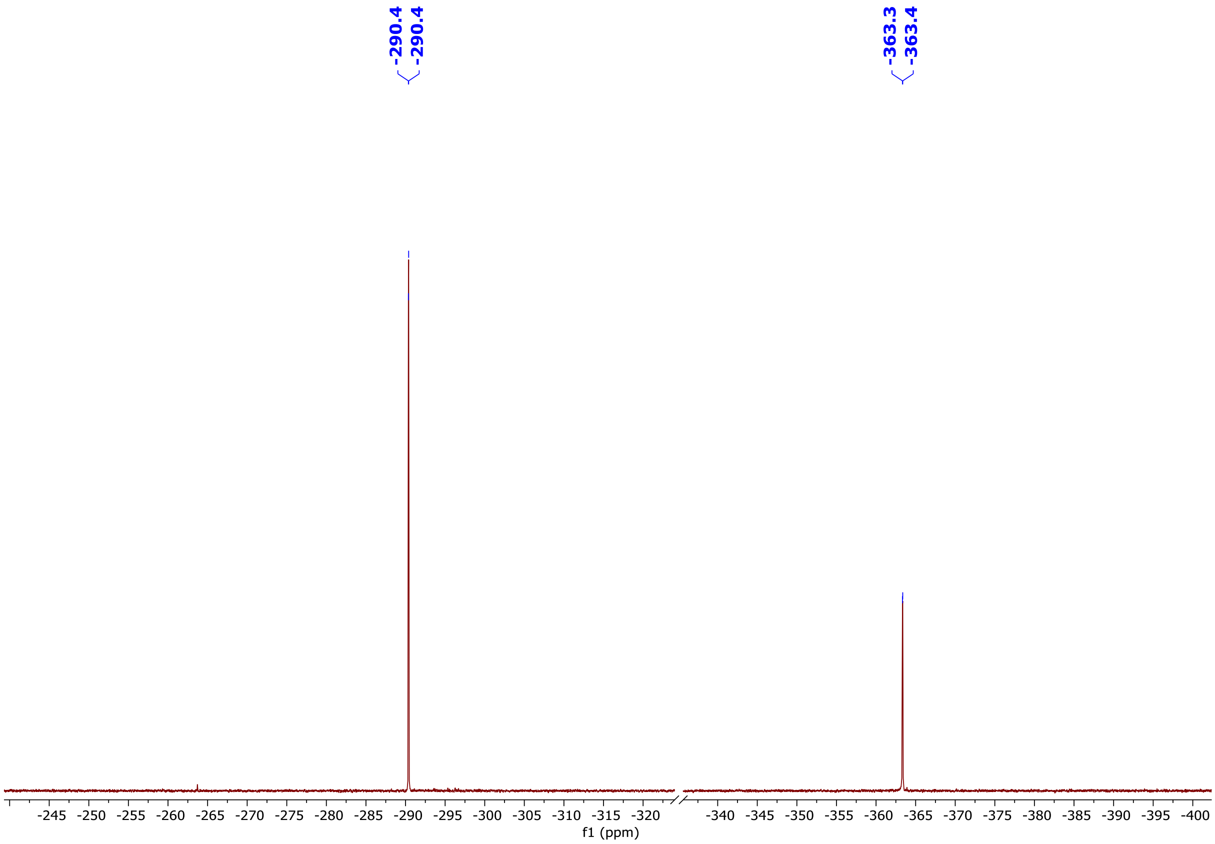

Figure S9. $^{31}P\{^1H\}$ NMR (162 MHz, C_6D_6, 298 K) spectrum of compound **3**.

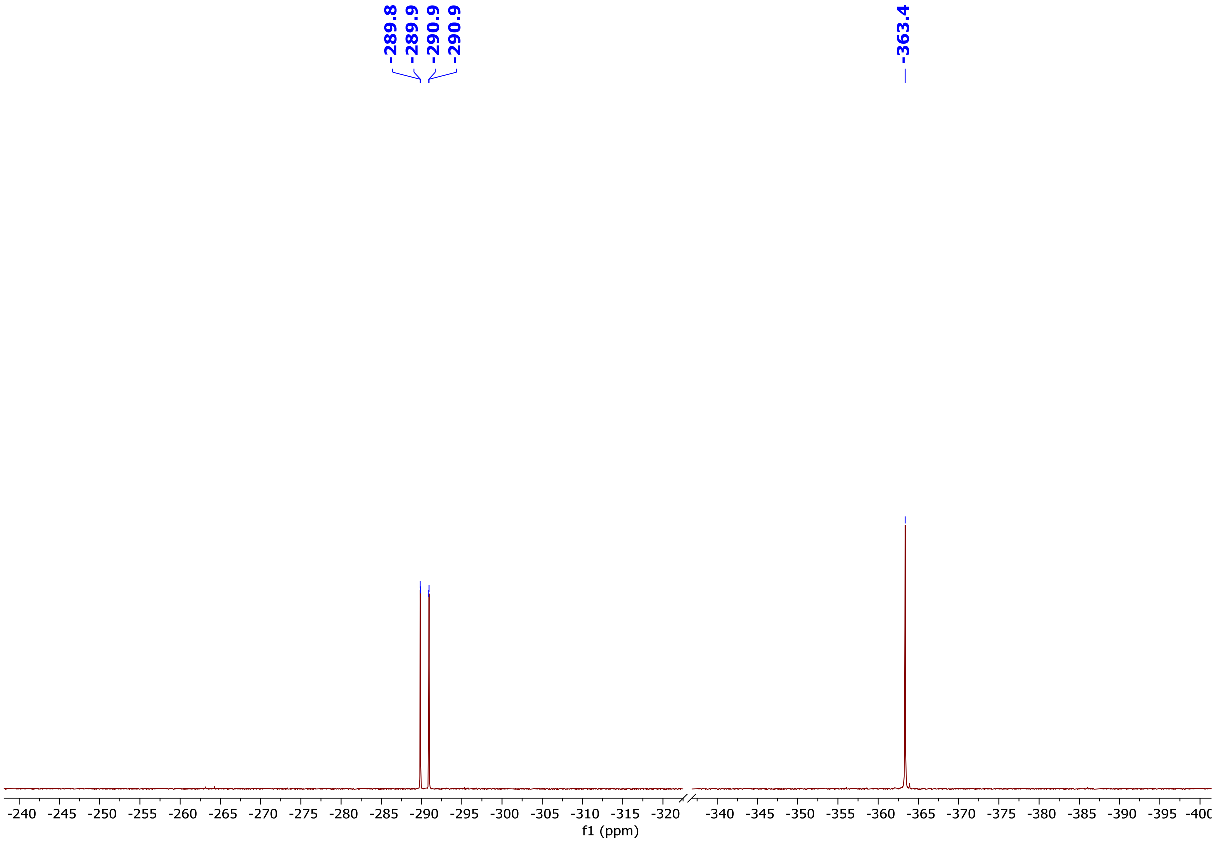

Figure S9. ^{31}P NMR (162 MHz, C_6D_6, 298 K) spectrum of compound **3**.

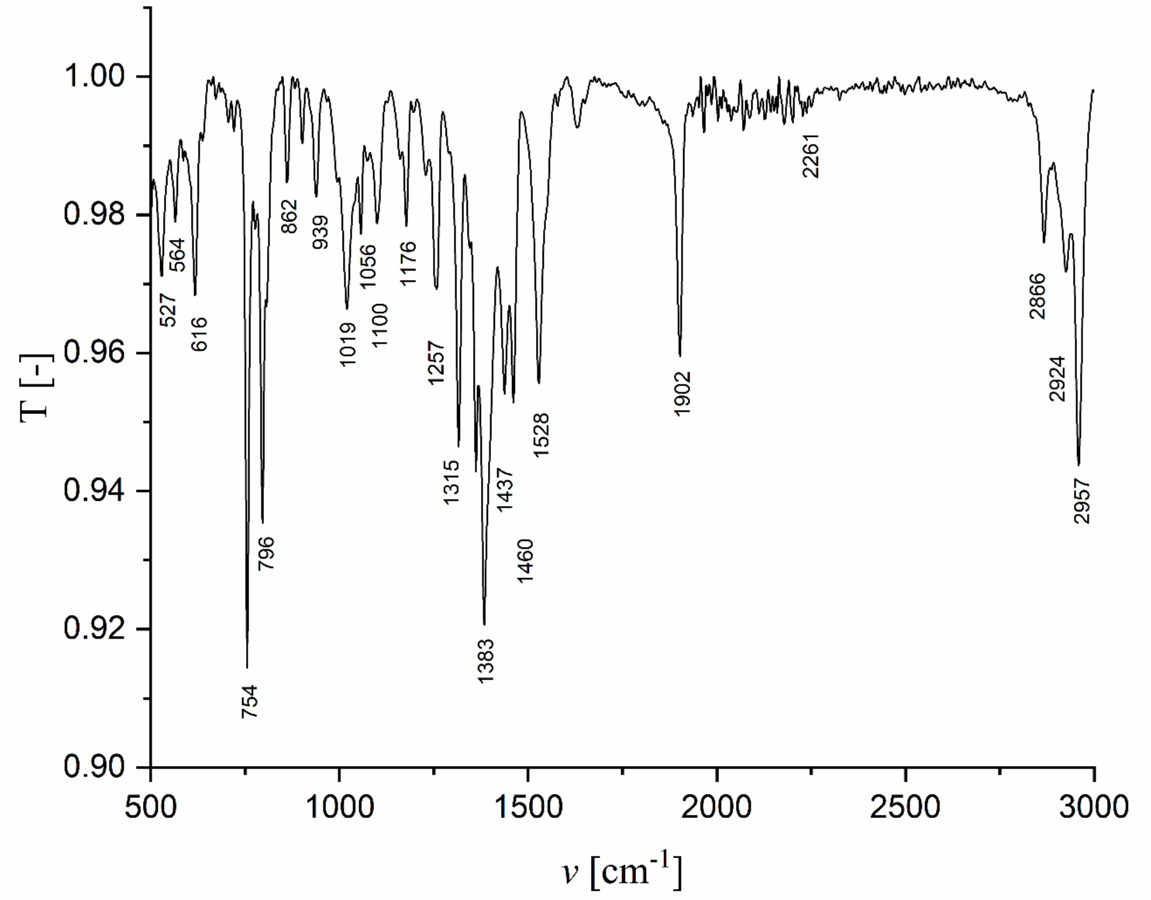

Figure S11. ATR-IR spectrum of **3**.

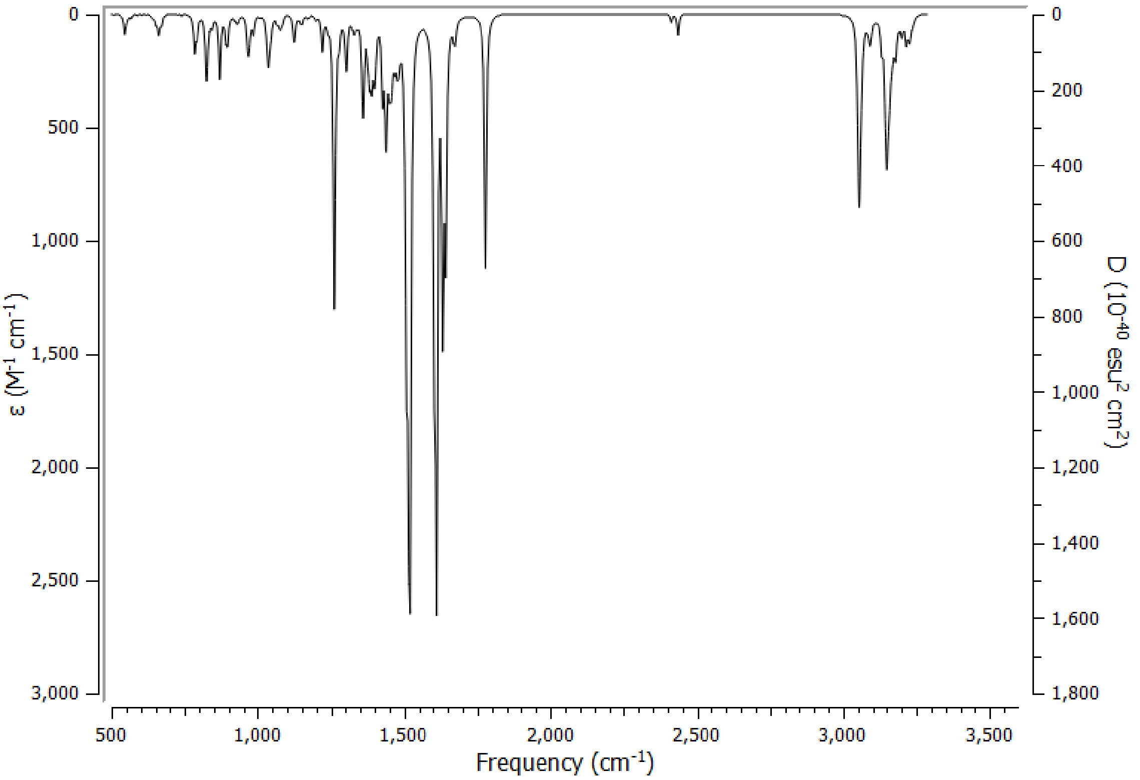

Figure S12. IR spectrum of compound **3** simulated by means of PBE0-D3BJ/def2-SVP. Selected stretching frequencies: C=O 1778 cm^{-1}, P-H 2413 cm^{-1} and 2436 cm^{-1}.

3. Crystallographic Details

The crystals were mounted on nylon loops in inert oil. Data were collected on a Bruker AXS D8 Venture diffractometer with Photon II detector (monochromated $Cu_{K\alpha}$ radiation, $\lambda = 1.54178$ Å, microfocus source) at 100(2) K. The structures were solved by Direct Methods (SHELXS-2013)[4] and refined anisotropically by full-matrix least-squares on F^2 (SHELXL-2017).[5,6] Absorption corrections were performed semi-empirically from equivalent reflections on basis of multi-scans (Bruker AXS APEX3). Hydrogen atoms were refined using a riding model or rigid methyl groups.

In **2** (mks_072m) the bridging N(H)C(O)P(H) moiety and Ga1 is disordered over two positions. In addition, the structure contains an impurity (less than 10%) of $L(H_2N)Ga(\mu\text{-}PH(PCO)GaL$. The displacement of all partially occupied positions was refined with RIGU and SIMU restraints. The alternate positions of Ga1 were refined with their displacement parameters constrained to be equal (EADP). Ga1 and Ga1' were refined with common co-ordinates (EXYZ). Due to their close proximity N5 and N5" were refined with common displacement parameters (EADP). The C=O bond length in the PCO group was restrained to be equal to 1.17 Å (DFIX). The P−H hydrogen atoms were refined freely. The one of the minor N(H)C(O)P(H) moiety could not be found but was included in the sum formula for completeness. The hydrogen atoms of the NH_2 group were placed at reasonable positions using shelxle's algorithm for non-planar NH_2 groups and refined by riding model (AFIX 3). All other NH hydrogen atoms were refined freely with its NH bond lengths restrained to be equal (SADI).

In **3** (mks_098m) one of the solvent molecules is disordered and could be crudely modelled with two alternate positions. The solvents' bond lengths and angles were restrained to be equal (SADI) and RIGU restraints applied to their displacement parameters. The residual electron density suggests that the OC(O)PH moiety is disordered, however it was not possible to refine an alternate orientation due to its low occupancy.

CCDC-2477073 (**2** (mks_072m)), and -2477074 (**3** (mks_098m)) contains the supplementary crystallographic data for this paper. These data can be obtained free of charge from The Cambridge Crystallographic Data Centre via www.ccdc.cam.ac.uk/data_request/cif.

Table S1. Crystal data and structure refinement of compounds **2** and **3**.

Compound	**2** (mks_072)	**3** (mks_098)
Emp. formula	$C_{59}H_{85}Ga_2N_5OP_2$	$C_{68}H_{105}Ga_2N_4O_2P_2$
Formula weight	1081.68	1211.93
Temperature [K]	100(2)	100(2)
Crystal system	monoclinic	triclinic
Space group	*Pn*	*P*-1
a [Å]	11.9829(5)	12.8211(5)
b [Å]	13.4660(8)	13.2204(5)
c [Å]	18.3564(9)	21.3512(8)
α [°]	90	106.7314(10)
β [°]	97.820(3)	106.5234(10)
γ [°]	90	90.3870(11)
V [Å^3]	2934.5(3)	3307.2(2)
Z	2	2
ρ [Mgm^{-3}]	1.224	1.217
μ [mm^{-1}]	1.954	1.791
F (000)	1148	1298
Crystal size [mm]	0.212 × 0.174 × 0.152	0.305 × 0.302 × 0.092
θmax [°]	79.998	79.368
Index ranges	$-15 \leq h \leq 15$<br>$-17 \leq k \leq 17$<br>$-23 \leq l \leq 23$	$-16 \leq h \leq 16$<br>$-15 \leq k \leq 16$<br>$-27 \leq l \leq 26$
No. of reflect. collected	110433	148946
Unique reflect.	12317	14232
R_{int}	0.0477	0.0292
Data / restraints / params.	12317 / 248 / 722	14232 / 165 / 793
Goodness-of-fit on F^2	1.056	1.018
$R1$ [I>2$\sigma(I)$]	0.0225	0.0324
$wR2$ [I>2$\sigma(I)$]	0.0563	0.0870
$R1$ [all data]	0.0244	0.0330
$wR2$ [all data]	0.0576	0.0876
Flack-Parameter *x*	-0.003(5)	–
Largest diff. peak and hole max./min.[e·Å^{-3}]	0.433/-0.330	1.421/-0.758

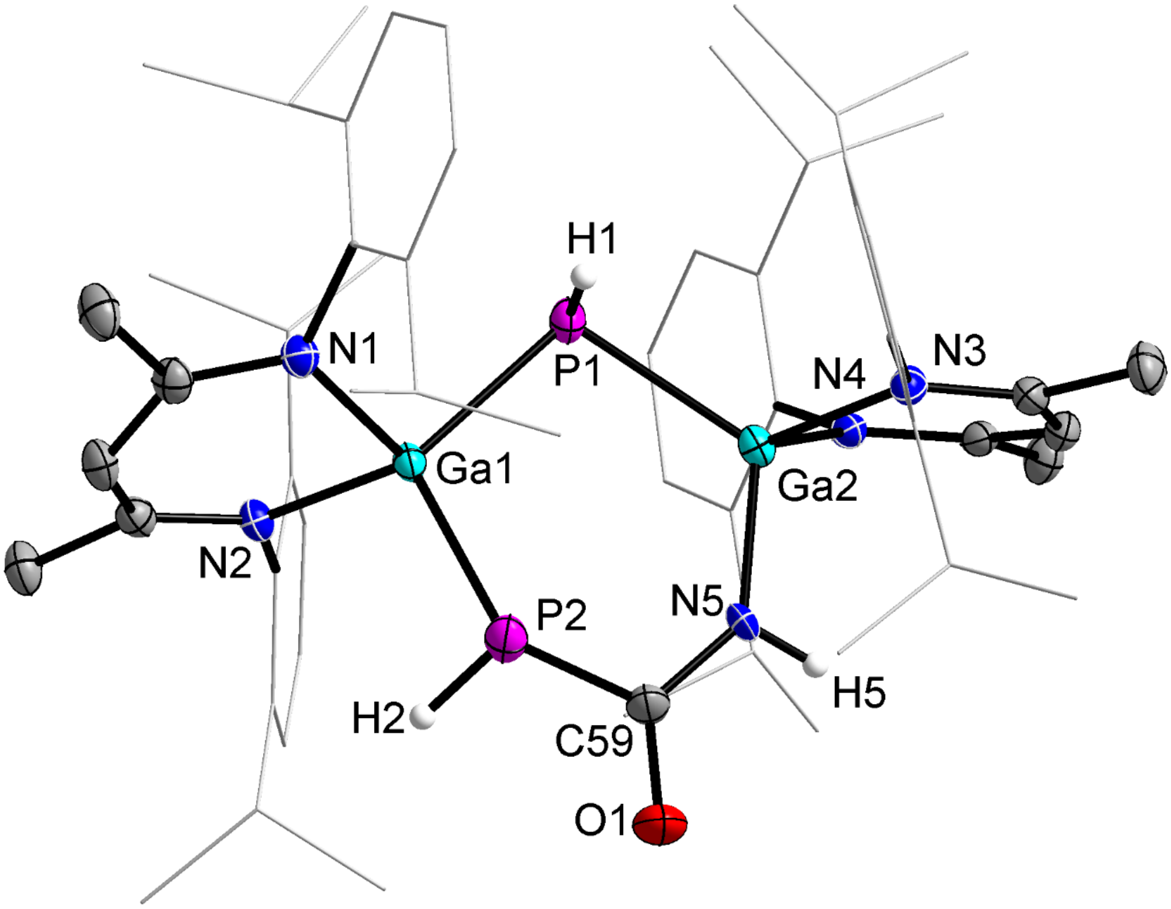

Figure S13. Molecular structure of **2** with thermal ellipsoids at 50% probability level. The hydrogen atoms and the minor disordered parts are omitted for clarity. Selected bond length (Å) and angels (°): Ga(1)-N(1) 1.996(2), Ga(1)-N(2) 2.000(2), Ga(1)-P(1) 2.3185(9), Ga(1)-P(2) 2.3715(18), P(2)-C(59) 1.873(5), O(1)-C(59) 1.231(5), N(5)-C(59) 1.379(7), N(5)-Ga(2) 1.847(5), P(1)-Ga(2) 2.3180(6), Ga(2)-N(3) 1.9766(18), Ga(2)-N(4) 1.9866(19); N(1)-Ga(1)-N(2) 94.06(8), N(1)-Ga(1)-P(1) 106.03(6), N(2)-Ga(1)-P(1) 121.76(6), N(1)-Ga(1)-P(2) 109.48(7), N(2)-Ga(1)-P(2) 112.12(7), P(1)-Ga(1)-P(2) 111.26(4), C(59)-P(2)-Ga(1) 109.41(15), C(59)-N(5)-Ga(2) 136.8(4), O(1)-C(59)-N(5) 122.0(5), O(1)-C(59)-P(2) 116.5(4), N(5)-C(59)-P(2) 121.0(3).

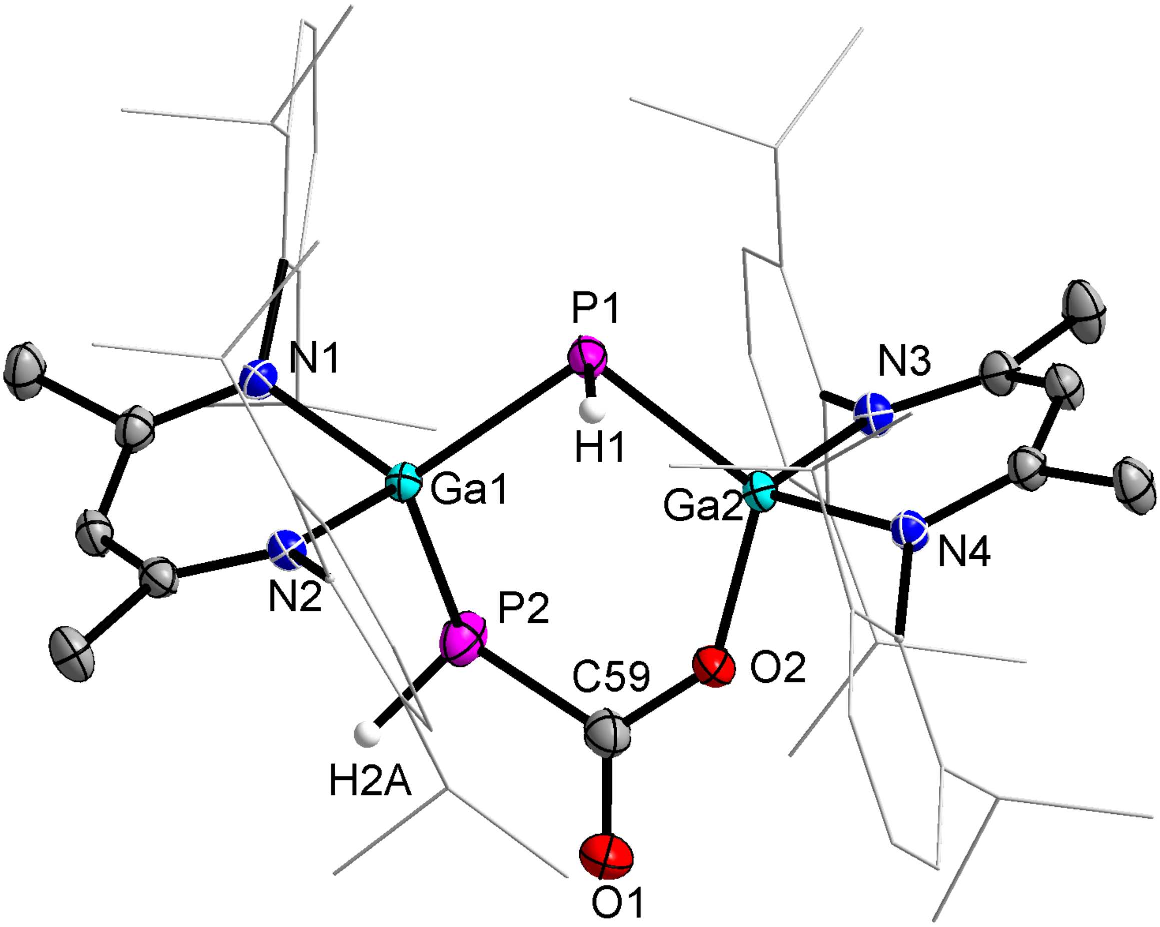

Figure S14. Molecular structure of **3** with thermal ellipsoids at 50% probability level. The hydrogen atoms and solvent molecules (*n*-hexane) are omitted for clarity. Selected bond length (Å) and angels (°): Ga(1)-N(2) 1.9726(12), Ga(1)-N(1) 1.9876(12), Ga(1)-P(1) 2.3367(4), Ga(1)-P(2) 2.3589(5), Ga(2)-O(2) 1.8668(11), Ga(2)-N(4) 1.9682(12), Ga(2)-N(3) 1.9769(12), Ga(2)-P(1) 2.3248(4), P(2)-C(59) 1.8524(17), O(1)-C(59) 1.222(2), O(2)-C(59) 1.3231(19); N(2)-Ga(1)-N(1) 94.81(5), N(2)-Ga(1)-P(1) 114.02(4), N(1)-Ga(1)-P(1) 116.08(4), N(2)-Ga(1)-P(2) 116.30(4), N(1)-Ga(1)-P(2) 101.53(4), P(1)-Ga(1)-P(2) 112.365(15), O(2)-Ga(2)-N(4) 99.44(5), O(2)-Ga(2)-N(3) 108.24(5), N(4)-Ga(2)-N(3) 95.27(5), O(2)-Ga(2)-P(1) 116.39(3), N(4)-Ga(2)-P(1) 117.31(4), N(3)-Ga(2)-P(1) 117.02(4), Ga(2)-P(1)-Ga(1) 99.341(15), C(59)-P(2)-Ga(1) 105.41(5), C(59)-O(2)-Ga(2) 138.99(10).

4. Computational Calculations

All calculations were performed by using the program packages Gaussian 16[7]. The geometrical parameters of the stationary points were optimized by means of the density functional methods functionals PBE0[8] with the empirical dispersion D3BJ[9]. As basis set def2-SVP[10,11] was employed. For all stationary points no symmetry restriction was applied. Frequency calculations were carried out at each of the structures to verify the nature of the stationary point. It turned out that all transition states have exactly one imaginary frequency, whereas all other structures have none. Furthermore, the energies of the stationary points were calculated using the density functionals PBE0-D3BJ and the basis set def2-TZVP[10]. To take solvent effects into account, the solvent model SMD[12] (THF as solvent) was used for the single point calculations.

A well-known problem during the calculation of the Gibbs energies is the overestimation of the calculated entropies. The entropies are always computed on the assumption of an ideal gas. This leads to a large deviation for the translation and conformational term of the entropy if the reaction takes place in solution.[13,14] This is particularly dramatic for bi- and trimolecular reactions. Some authors have estimated that the total entropy in solution should be about 50-70% of that in the gas phase.[14-17] In our case, we have therefore used the upper limit, i.e. 70% (instead of 100%) of the calculated entropy. The thus obtained the Gibbs energies G70% were used for comparison with experimental data.

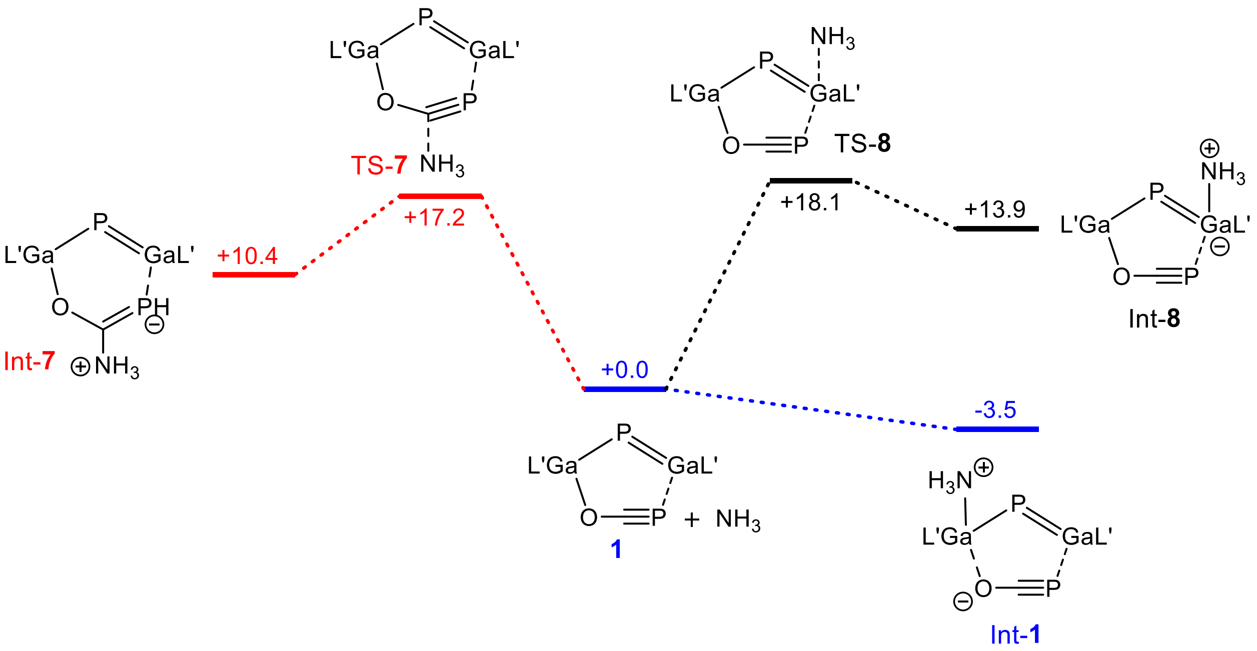

Figure S15. Gibbs energies ($G_{70\%}$) for the nucleophile attack of NH_3 at gallaphosphene **1** calculated by means of PBE0-D3BJ/def2-TZVP(SMD,THF)//PBE0-D3BJ/def2-SVP. L' = HC[C(Me)NAr]$_2$ and Ar = 2,6-iPr$_2$C$_6$H$_3$. Herein $G_{70\%}$ means that 70% calculated gas-phase entropy contributes to solution-phase free energy. The values are given in kcal/mol.

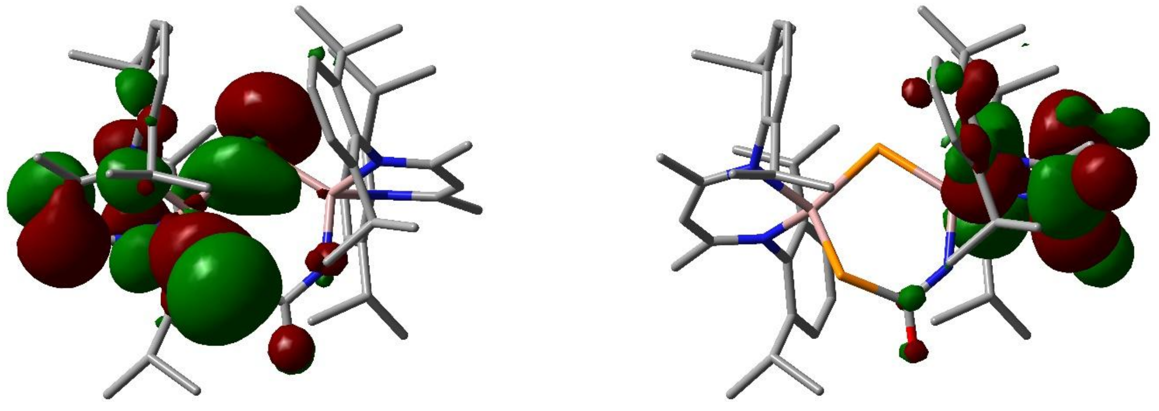

-5.52 -1.04

Figure S16. HOMO (left) and LUMO (right) of **2** calculated by means of PBE0-D3BJ/def2-TZVP(SMD,THF)//PBE0-D3BJ/def2-SVP. The energies are given in eV.

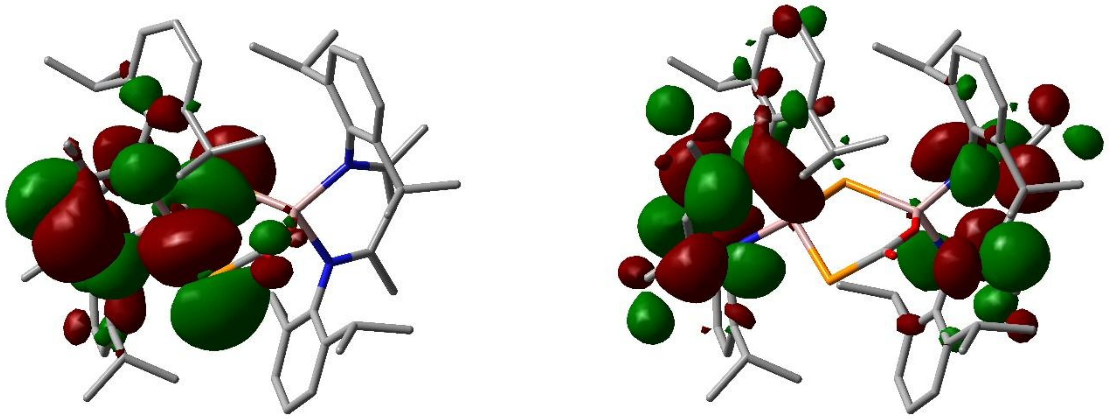

-5.70 -1.13

Figure S17. HOMO (left) and LUMO (right) of **3** calculated by means of PBE0-D3BJ/def2-TZVP(SMD,THF)//PBE0-D3BJ/def2-SVP. The energies are given in eV.

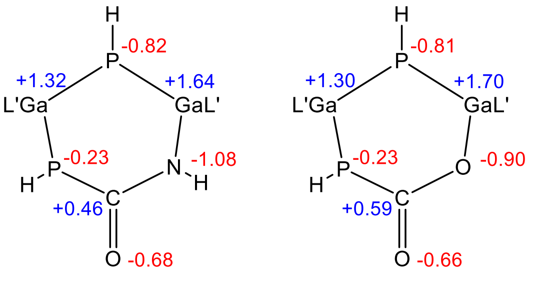

2 **3**

Figure S18. Natural charges from the NBO analysis of **2** and **3** calculated by means of PBE0-D3BJ/def2-TZVP(SMD,THF)//PBE0-D3BJ/def2-SVP. L' = HC[C(Me)NAr]$_2$ and Ar = 2,6-*i*-Pr$_2$C$_6$H$_3$

Table S2. Absolute energies [au] of the calculated compounds by means of different methods.

Compound	E^a	G^a	E^b
H_2O	-76.276604	-76.273310	-76.384270
NH_3	-56.441327	-56.425962	-56.518331
**1**	-7118.383810	-7117.199557	-7121.837100
Int-**1**	-7174.853196	-7173.634452	-7178.374413
TS-**1a**	-7174.814777	-7173.600756	-7178.332427
TS-**1b**	-7231.292351	-7230.042144	-7234.873375
Int-**2**	-7174.850973	-7173.637646	-7178.370055
TS-**2**	-7174.848973	-7173.632696	-7178.369011
Int-**3**	-7174.870242	-7173.656323	-7178.387821
TS-**3**	-7174.851485	-7173.634706	-7178.373471
Int-**4**	-7174.861680	-7173.639974	-7178.391246
TS-**4a**	-7174.824607	-7173.608619	-7178.345938
TS-**4b**	-7231.313425	-7230.057767	-7234.901752
Int-**5**	-7174.870708	-7173.650181	-7178.393648
TS-**5**	-7174.867610	-7173.646977	-7178.388744
Int-**6**	-7174.885801	-7173.663712	-7178.403099
TS-**6a**	-7174.849668	-7173.633208	-7178.366920
TS-**6b**	-7231.338054	-7230.085953	-7234.920861
**2**	-7174.905562	-7173.686215	-7178.423544
**3**	-7194.749549	-7193.541646	-7198.291136

[a] PBE0-D3BJ/def2-SVP.

[b] PBE0-D3BJ(THF as solvent)/def2-TZVP//PBE0-D3BJ/def2-SVP

Cartesian coordinates of the optimized geometry of H_2O at PBE0-D3BJ/def2-SVP level of theory:

O	0.00000000	0.11963300	0.00000000
H	0.75406400	-0.47856400	0.00000000
H	-0.75406400	-0.47850200	0.00000000

Cartesian coordinates of the optimized geometry of NH_3 at PBE0-D3BJ/def2-SVP level of theory:

N	0.00000300	0.00000100	-0.12157200
H	0.92837700	-0.11942300	0.28367300
H	-0.56762100	-0.74428800	0.28366200
H	-0.36077700	0.86370300	0.28366700

Cartesian coordinates of the optimized geometry of **1** at PBE0-D3BJ/def2-SVP level of theory:

Ga	-1.66109000	-0.08304500	0.63838200
Ga	1.64991200	0.09709200	-0.45904800
P	0.42013800	0.05036500	1.41163400
P	0.76631800	0.13427300	-2.90049100
O	-1.63138100	-0.13907400	-1.45648100
C	-0.61334300	-0.02173700	-2.09488700
N	-3.07971700	1.28608500	0.87459700
N	-2.90685900	-1.59180500	0.95118700
N	2.90701600	1.61834100	-0.74654400
N	3.03955000	-1.28208700	-0.84502500
C	-4.17808500	1.05078900	1.59536500
C	-4.56032600	-0.21857400	2.04766800
H	-5.45553600	-0.25537200	2.66508400
C	-4.01865400	-1.45489200	1.67698000
C	-5.08930300	2.19204900	1.95049200
H	-5.98575900	1.82857200	2.46563000
H	-5.38901400	2.75787600	1.05828100
H	-4.56851100	2.90642100	2.60229400
C	-4.76432900	-2.67997600	2.12634300
H	-5.69309800	-2.40508900	2.63915300
H	-4.13992800	-3.26951600	2.81158000
H	-4.99911100	-3.34247600	1.28281400
C	-2.91965000	2.60837600	0.34859700

C	-2.30451500	3.61393600	1.12247500
C	-2.28359300	4.91657000	0.61262500
H	-1.81153500	5.70802100	1.19961600
C	-2.85801700	5.22334800	-0.61407600
H	-2.84479100	6.25129700	-0.98454900
C	-3.43096100	4.21352300	-1.37893700
H	-3.86264700	4.45404200	-2.35333400
C	-3.46313300	2.89310400	-0.92418800
C	-1.68343500	3.34442600	2.48037400
H	-1.83182800	2.27876100	2.71278300
C	-2.32752800	4.17420000	3.59337700
H	-3.41631300	4.03154400	3.66121400
H	-2.14699300	5.25055700	3.44441400
H	-1.89399700	3.90122600	4.56779700
C	-0.18037500	3.59944300	2.44368500
H	0.26906200	3.37415000	3.42332700
H	0.04119800	4.65057000	2.20613700
H	0.31123700	2.95773400	1.69995500
C	-4.10948800	1.81919900	-1.78152200
H	-3.84376400	0.84958500	-1.34123900
C	-5.63554700	1.92027700	-1.77490800
H	-6.07553300	1.13212700	-2.40653300
H	-5.97311100	2.89322500	-2.16712600
H	-6.05073900	1.80230400	-0.76372700
C	-3.57256200	1.83011100	-3.21108700
H	-3.96157800	0.96593200	-3.77103100
H	-2.47520000	1.77824800	-3.22847100
H	-3.87715400	2.73740500	-3.75650200
C	-2.61264400	-2.89992200	0.45074100
C	-1.83070200	-3.79919700	1.20325100
C	-1.67051200	-5.09997900	0.71224600
H	-1.06701100	-5.81066700	1.28191600
C	-2.27226100	-5.50650100	-0.47162700
H	-2.14828500	-6.53290200	-0.82561800
C	-3.01700900	-4.59729600	-1.21555700
H	-3.47102500	-4.91479200	-2.15734800
C	-3.18940500	-3.28095200	-0.78243000
C	-1.18163300	-3.42151800	2.52163900
H	-1.42118600	-2.36627700	2.72304700
C	-1.69981000	-4.26652300	3.68767800
H	-2.79242500	-4.21427100	3.80401100
H	-1.24714000	-3.92672800	4.63196600
H	-1.43480100	-5.32805100	3.55898100
C	0.33627200	-3.53415500	2.43272700
H	0.80046000	-3.20022300	3.37327800
H	0.73216000	-2.89988400	1.62856600
H	0.65097600	-4.57268900	2.25168400
C	-4.01304100	-2.31424500	-1.61653500
H	-3.85537500	-1.31084000	-1.19906700
C	-5.51060000	-2.61432200	-1.53271500
H	-5.73537700	-3.62791200	-1.90226900
H	-6.08023900	-1.89877900	-2.14671200
H	-5.88915300	-2.53968500	-0.50348300
C	-3.55307100	-2.27610400	-3.07192300
H	-3.73552300	-3.23327000	-3.58550700
H	-2.48128100	-2.04921200	-3.14515300
H	-4.10348600	-1.49876800	-3.62406600
C	3.94184700	1.50069800	-1.57550000
C	4.37960200	0.27671600	-2.10825100
H	5.20953100	0.33863400	-2.80974700
C	4.05018300	-1.01493400	-1.67004300
C	4.73682800	2.72069300	-1.95034900
H	5.04932500	3.27306100	-1.05255500
H	4.12592200	3.41524500	-2.54378200
H	5.62427800	2.44472800	-2.53143600
C	4.94045600	-2.13460700	-2.13087800
H	5.41638700	-2.62604200	-1.26970500
H	5.72051800	-1.76313600	-2.80528000
H	4.35844300	-2.91209500	-2.64445400
C	2.66654700	2.89613900	-0.15016300
C	3.13034900	3.12684800	1.16257300

C	3.01796300	4.41605300	1.68834200
H	3.38526600	4.61733400	2.69582100
C	2.43219800	5.44410500	0.95849900
H	2.35521900	6.44594400	1.38795000
C	1.91645300	5.18336300	-0.30370700
H	1.42019600	5.98180900	-0.86005400
C	2.02198800	3.91430400	-0.88105800
C	3.75341000	2.01266500	1.98056800
H	3.27450700	1.07723500	1.65073700
C	5.25440700	1.88783500	1.71671000
H	5.69161900	1.08926400	2.33610900
H	5.77478500	2.82782000	1.96181500
H	5.46830000	1.64387100	0.66608900
C	3.46544700	2.14706300	3.47143700
H	2.38499700	2.23477800	3.65462600
H	3.97035500	3.01856300	3.91721100
H	3.82592100	1.25761200	4.00722000
C	1.39972700	3.65974200	-2.23786000
H	1.76645600	2.68803000	-2.60154900
C	1.75802600	4.71474400	-3.28248200
H	1.36149700	4.42197000	-4.26684400
H	2.84586600	4.85149100	-3.38352700
H	1.32398400	5.69659800	-3.03677700
C	-0.11404800	3.54311400	-2.08868800
H	-0.38694600	2.79011100	-1.33531500
H	-0.58205800	3.25776700	-3.04302100
H	-0.54921500	4.49907100	-1.76459200
C	2.94810000	-2.60287800	-0.30238100
C	3.57124200	-2.87234000	0.93336000
C	3.54465600	-4.18029500	1.42449500
H	4.03037000	-4.40133400	2.37821000
C	2.90925600	-5.19923300	0.72652100
H	2.89689000	-6.21563400	1.12754700
C	2.27861400	-4.91501200	-0.47815700
H	1.76614000	-5.71274000	-1.02085800
C	2.29249700	-3.62586400	-1.01795100
C	4.28299300	-1.79690900	1.72880800
H	4.17232300	-0.85243100	1.17683800
C	5.77973500	-2.08548300	1.84651100
H	6.29128200	-1.27134300	2.38320400
H	6.25352700	-2.18457000	0.85801600
H	5.96779000	-3.01846000	2.40128000
C	3.63773000	-1.60842800	3.10019800
H	2.58634300	-1.29882000	2.99879100
H	4.17247500	-0.83652000	3.67397700
H	3.66849400	-2.53801900	3.69047700
C	1.59654400	-3.36397700	-2.33598000
H	1.87105000	-2.35017000	-2.66359800
C	2.01278300	-4.33723300	-3.43744300
H	1.68473500	-5.36570500	-3.21930400
H	3.10422100	-4.36232900	-3.58022600
H	1.55402300	-4.04438100	-4.39448300
C	0.08509400	-3.38178900	-2.14216000
H	-0.26357100	-4.38416000	-1.85584800
H	-0.43098100	-3.09033400	-3.06927100
H	-0.22631900	-2.69241800	-1.34465600

Cartesian coordinates of the optimized geometry of **2** at PBE0-D3BJ/def2-SVP level of theory:

Ga	1.20797300	1.31396000	-0.47125700
P	0.42775700	1.17861900	-2.67397300
H	1.63031200	1.26754100	-3.42403400
O	0.51058000	-1.05786700	-4.14905400
N	-0.75159300	-1.33018700	-2.28726100
H	-0.95943700	-2.21230800	-2.75785400
C	0.08440600	-0.62204800	-3.09041400
C	2.07571300	4.07008000	-0.05232700
N	1.07040600	3.21515100	0.13462500
P	-0.08543400	0.05897300	1.03171000

H	-1.08124700	1.08555000	0.98256200
Ga	-1.23066500	-1.30456800	-0.44315600
C	3.37483600	3.67905700	-0.38954500
H	4.09664800	4.48702500	-0.50273900
N	3.20939700	1.26327900	-0.33864800
N	-1.11268600	-3.20050400	0.11134700
C	3.92255500	2.38386500	-0.38011200
N	-3.22194200	-1.29130800	-0.44673900
C	1.83898200	5.54518600	0.12023700
H	1.30023500	5.93015300	-0.75875600
H	2.78695300	6.08870900	0.20811000
H	1.21186400	5.76115500	0.99493700
C	-2.36101800	4.61375400	2.15218500
H	-3.24262900	4.97974900	2.68403000
C	-2.29244800	4.70343400	0.76902900
H	-3.12715900	5.13876200	0.21452100
C	-1.17058300	4.25186400	0.06491500
C	-0.10874300	3.68061100	0.79279800
C	5.42822500	2.34306000	-0.39093200
H	5.82309200	3.08957800	0.31128000
H	5.79186100	2.61513700	-1.39309000
H	5.83118200	1.35735700	-0.13474400
C	-0.17043500	3.56719200	2.20164400
C	-1.30337000	4.05042100	2.85933600
H	-1.36142700	3.98026900	3.94748400
C	-1.13890000	4.39212700	-1.44265400
H	-0.16892000	4.00744200	-1.79317200
C	-1.27530200	5.84672700	-1.89544200
H	-2.26621300	6.25310200	-1.63767600
H	-1.16534900	5.91810100	-2.98865200
H	-0.52409500	6.50431300	-1.43396700
C	0.98376800	2.98560300	2.99718400
H	1.56742700	2.36294500	2.30304000
C	-2.22506500	3.53771600	-2.08439100
H	-2.18322800	2.50150000	-1.72495800
H	-2.10627900	3.51517500	-3.17758200
H	-3.22642600	3.93042600	-1.85134000
C	1.91414900	4.08727700	3.50909600
H	1.37294500	4.78120200	4.17249400
H	2.34631500	4.67461600	2.68707600
H	2.74911100	3.65284000	4.08080000
C	0.52818300	2.08609200	4.14156300
H	-0.01123700	2.64620400	4.92151600
H	1.40133900	1.62201400	4.62490900
H	-0.12342900	1.27948500	3.77612600
C	3.87897600	0.00078600	-0.27458200
C	4.50656000	-0.54567100	-1.41660400
C	5.17016000	-1.76956700	-1.27793800
H	5.66765000	-2.20230700	-2.14922000
C	5.20127200	-2.44696400	-0.06717400
H	5.71775500	-3.40675200	0.01193100
C	4.57431300	-1.89845900	1.04565400
H	4.60424200	-2.43077400	1.99715200
C	3.92156000	-0.66644700	0.97064900
C	4.54352800	0.14528900	-2.76911000
H	4.02942200	1.11337200	-2.66788800
C	5.98274800	0.40867000	-3.22460100
H	6.58745600	0.91057700	-2.45699800
H	5.98769200	1.03435700	-4.13062100
H	6.49311300	-0.53365300	-3.47872700
C	3.81011300	-0.65216400	-3.84642800
H	2.74732100	-0.80222300	-3.62392400
H	4.27060700	-1.64359900	-3.98445200
H	3.86721800	-0.12536700	-4.81189000
C	3.36846500	-0.01369200	2.22216600
H	2.51242400	0.60373900	1.91447100
C	4.40449300	0.92084400	2.84941700
H	3.99533600	1.39677300	3.75461400
H	4.70721800	1.72204800	2.16109500
H	5.31003500	0.36373500	3.13879300
C	2.84252600	-1.01038300	3.24356100

H	2.32945500	-0.47907900	4.05848200
H	3.64997500	-1.60454100	3.70092300
H	2.12340200	-1.70282200	2.78747200
C	-2.08318300	-4.07537500	-0.15322700
C	-3.36890000	-3.70251700	-0.55907800
H	-4.06675700	-4.51847400	-0.74070400
C	-3.92591400	-2.41113000	-0.57619100
C	-1.81107100	-5.54494500	0.00814400
H	-1.37268100	-5.76730800	0.99056300
H	-1.07342400	-5.87156100	-0.74027000
H	-2.72759900	-6.13223300	-0.12057600
C	-5.42014400	-2.35278400	-0.74529200
H	-5.88818600	-3.23652900	-0.29383400
H	-5.66920700	-2.35521800	-1.81715800
H	-5.85360000	-1.44506100	-0.30856700
C	0.04336900	-3.65897100	0.81215800
C	1.14546000	-4.17796600	0.10600200
C	2.23964300	-4.64741200	0.83850100
H	3.10799800	-5.04189700	0.30609600
C	2.23506800	-4.62816200	2.22774600
H	3.09396400	-5.01173800	2.78393600
C	1.13871100	-4.11130200	2.91053200
H	1.14577500	-4.09147000	4.00270700
C	0.03304000	-3.60429200	2.22329000
C	1.16278100	-4.22385700	-1.40737900
H	0.14469600	-3.98742300	-1.74954700
C	1.52787600	-5.60250900	-1.95503200
H	0.87492000	-6.39500700	-1.55758700
H	2.56549500	-5.87447800	-1.70563300
H	1.44486400	-5.60932900	-3.05268200
C	2.09321500	-3.15565300	-1.97208600
H	1.89892600	-2.17317900	-1.51912500
H	1.95736500	-3.05063900	-3.05793800
H	3.14386300	-3.40451100	-1.76675100
C	-1.16259400	-3.06096100	2.98514700
H	-1.76053400	-2.48031300	2.26705400
C	-2.05098400	-4.19132800	3.50807100
H	-2.91819000	-3.78211700	4.04984100
H	-1.49452100	-4.84145600	4.20246200
H	-2.43667800	-4.82078400	2.69357500
C	-0.76496100	-2.11272200	4.11211200
H	-1.66575300	-1.69542500	4.58857000
H	-0.16565200	-1.27707300	3.72421800
H	-0.18881200	-2.62250900	4.90042700
C	-3.89447400	-0.03252800	-0.42888700
C	-4.38012100	0.53467300	-1.62672000
C	-5.05661500	1.75594000	-1.54529700
H	-5.44515700	2.20940200	-2.46026400
C	-5.22609000	2.40995100	-0.33262500
H	-5.74841600	3.36905500	-0.29560100
C	-4.72099300	1.84795400	0.83493200
H	-4.84934000	2.37287600	1.78239500
C	-4.06057700	0.61753500	0.81484900
C	-4.20336900	-0.12483300	-2.98365700
H	-3.75299300	-1.11484600	-2.81950700
C	-5.53796200	-0.31447300	-3.70820300
H	-6.28863200	-0.82173900	-3.08473600
H	-5.39441500	-0.90727500	-4.62475600
H	-5.96714800	0.65317900	-4.01218000
C	-3.23673400	0.65845900	-3.87025000
H	-3.64247700	1.65290100	-4.11529500
H	-3.06296500	0.12183500	-4.81575400
H	-2.26287200	0.80274400	-3.38628400
C	-3.59909600	-0.03974900	2.10287900
H	-2.67379300	-0.59070500	1.87120300
C	-4.61804700	-1.06619600	2.60196000
H	-4.27100300	-1.52359000	3.54194500
H	-4.77552500	-1.87865800	1.87955900
H	-5.59174300	-0.58869600	2.79639600
C	-3.25760400	0.95570600	3.20354200
H	-4.15590600	1.45427400	3.60183600

H	-2.56759500	1.73268000	2.84710000
H	-2.77631300	0.43478100	4.04451000

Cartesian coordinates of the optimized geometry of **3** at PBE0-D3BJ/def2-SVP level of theory:

Ga	1.56944900	0.50441500	-0.60883500
Ga	-1.56258800	-0.61285100	0.50850600
P	0.36567600	0.34944700	1.39782100
H	-0.16252200	1.65657500	1.16117400
P	0.66903900	-0.87046700	-2.28900100
H	1.12272400	-0.21455600	-3.46772600
O	-1.58848600	-0.04841600	-3.49912700
O	-1.83001800	-0.35090600	-1.32330800
N	3.50133700	-0.02743600	-0.58307100
N	1.94495600	2.40731100	-1.06313400
N	-1.95381400	-2.50492800	0.89270700
N	-3.27036200	0.09389700	1.20787400
C	4.35711100	0.61496400	-1.37531400
C	4.04831500	1.79051100	-2.08053700
H	4.82617800	2.15465300	-2.74949000
C	3.00019400	2.68678700	-1.83136400
C	5.76568200	0.10810300	-1.51745200
H	6.21416400	-0.08747500	-0.53342000
H	6.38563900	0.83088500	-2.05978400
H	5.78201900	-0.84666200	-2.05905600
C	3.16020700	4.06594200	-2.41247100
H	2.19890500	4.50284900	-2.70895300
H	3.83445500	4.03460100	-3.27762000
H	3.60000800	4.74202300	-1.66440300
C	4.00145300	-1.13070500	0.17850400
C	4.35105200	-0.91038200	1.52898600
C	4.96014000	-1.94748300	2.23971900
H	5.25039600	-1.78498300	3.27934400
C	5.20718100	-3.17956800	1.64620600
H	5.69332400	-3.97587300	2.21480800
C	4.81135600	-3.39842800	0.33374400
H	4.97850600	-4.37711700	-0.12240100
C	4.19871400	-2.39375900	-0.42275500
C	4.13075600	0.43448100	2.19506000
H	3.32364900	0.94016300	1.64370300
C	5.38134200	1.30872800	2.08657600
H	5.22706300	2.27659600	2.58817600
H	5.64254700	1.51688300	1.03871400
H	6.24658400	0.81760500	2.56022900
C	3.66640300	0.30130600	3.64185700
H	2.76315600	-0.32235800	3.70735700
H	3.41814600	1.28961300	4.05373200
H	4.44213600	-0.13449500	4.29069800
C	3.74267000	-2.72821600	-1.83119500
H	3.36427900	-1.80341900	-2.29418800
C	4.86007400	-3.28331900	-2.71621100
H	5.21296600	-4.26047600	-2.35085400
H	5.73631400	-2.62068200	-2.77136100
H	4.48892900	-3.43387700	-3.74147300
C	2.58664300	-3.72510000	-1.77694200
H	2.92209600	-4.68899000	-1.36247800
H	2.17848900	-3.90832600	-2.78236800
H	1.76139700	-3.35641000	-1.15611600
C	1.15168400	3.52187800	-0.64524500
C	1.63508400	4.36041700	0.38074700
C	0.93682900	5.53872700	0.66325200
H	1.30463800	6.20283500	1.44934000
C	-0.21911300	5.87122200	-0.02991700
H	-0.74864500	6.79932000	0.19851000
C	-0.72263500	4.99943200	-0.98941100
H	-1.65632900	5.24046300	-1.49850300
C	-0.05753200	3.81321200	-1.31286300
C	2.85052100	4.00380300	1.21570600
H	3.31094800	3.10745800	0.77468600

C	3.90837800	5.10612800	1.23548200
H	3.53793300	6.01670600	1.73198500
H	4.22942600	5.38879900	0.22205300
H	4.80036500	4.77118000	1.78720800
C	2.41357600	3.64852000	2.63768400
H	1.89423300	4.49534300	3.11348800
H	3.28465800	3.39900500	3.26216600
H	1.72932300	2.78726300	2.64341300
C	-0.62136700	2.86279100	-2.34769200
H	-0.39742000	1.85233000	-1.97253000
C	0.04320200	2.97191000	-3.71977400
H	-0.42523300	2.24557100	-4.40030700
H	1.11722900	2.74495400	-3.68689500
H	-0.08614200	3.98223500	-4.14129100
C	-2.13192400	2.96395200	-2.48402800
H	-2.49725800	2.12950100	-3.09685200
H	-2.43709200	3.90224700	-2.97530900
H	-2.62724000	2.91223100	-1.50672200
C	-2.88234300	-2.84417600	1.79211200
C	-3.74578100	-1.93843100	2.41853700
H	-4.42140700	-2.36605800	3.15808400
C	-4.00429900	-0.60455600	2.06889000
C	-3.05478300	-4.29109400	2.16468700
H	-2.20372900	-4.63780200	2.76729700
H	-3.08507700	-4.93147700	1.27289700
H	-3.97377700	-4.43324100	2.74468800
C	-5.22146800	-0.00059900	2.71818500
H	-6.08879800	-0.66056900	2.58085600
H	-5.46585900	0.99443900	2.33286000
H	-5.04159200	0.07153100	3.80187600
C	-1.21701700	-3.56660600	0.27403600
C	-1.60797600	-4.01560900	-1.00576700
C	-0.94426600	-5.12147400	-1.54562700
H	-1.23322100	-5.48565300	-2.53295400
C	0.06633500	-5.76969900	-0.84625100
H	0.56599000	-6.63674700	-1.28475000
C	0.45436700	-5.29909200	0.40162300
H	1.26778400	-5.79514000	0.93594700
C	-0.16822000	-4.19141800	0.98229500
C	-2.75572200	-3.37123900	-1.76126100
H	-2.80756600	-2.32245000	-1.44319900
C	-4.09582600	-4.01755200	-1.40431800
H	-4.34053200	-3.89779400	-0.33893200
H	-4.09283700	-5.09535700	-1.63513300
H	-4.90902900	-3.55009300	-1.98190400
C	-2.54092800	-3.36305900	-3.27221200
H	-2.64992900	-4.36780400	-3.71206800
H	-1.54541800	-2.97649500	-3.53412900
H	-3.28283300	-2.70884100	-3.75232900
C	0.31550500	-3.68529400	2.32829700
H	-0.42229400	-2.96053300	2.70466400
C	0.46139300	-4.79386100	3.37071000
H	0.70480100	-4.36100600	4.35310800
H	1.27681600	-5.48687600	3.11162000
H	-0.45527100	-5.39298000	3.48187900
C	1.63758300	-2.94383200	2.16392100
H	1.97093900	-2.51535800	3.12070600
H	1.55098500	-2.11889700	1.44543500
H	2.42831000	-3.61527000	1.79860400
C	-3.78736300	1.32597900	0.69349900
C	-4.66762400	1.27334900	-0.41009500
C	-5.22217300	2.47250700	-0.86473900
H	-5.91177000	2.45224600	-1.71168600
C	-4.90590000	3.68729500	-0.26686700
H	-5.34676900	4.61363400	-0.64274000
C	-4.01927000	3.72144200	0.80260200
H	-3.76950900	4.67836100	1.26640900
C	-3.44877500	2.54837400	1.30851200
C	-5.07389300	-0.03642600	-1.06197500
H	-4.41148500	-0.81978100	-0.66800500
C	-6.51094400	-0.40600600	-0.68602300

H	-6.65756800	-0.44809000	0.40236800
H	-6.77574500	-1.39078400	-1.10205800
H	-7.22577700	0.33042900	-1.08746500
C	-4.89859300	-0.02227800	-2.57807300
H	-5.20250800	-0.99573600	-2.99514800
H	-3.85126000	0.14997900	-2.86136100
H	-5.53036600	0.74349400	-3.05596800
C	-2.55764500	2.61830700	2.53478900
H	-2.07908700	1.63380700	2.65317300
C	-3.37125900	2.89589600	3.80124000
H	-4.14885700	2.13998200	3.97125400
H	-3.86983700	3.87646700	3.73744100
H	-2.71361100	2.91077700	4.68450900
C	-1.45333900	3.65928800	2.38874300
H	-1.85689100	4.68354200	2.38557200
H	-0.88919400	3.53210700	1.45554200
H	-0.74510400	3.58743200	3.22785600
C	-1.11014500	-0.32301200	-2.41960900

Cartesian coordinates of the optimized geometry of Int-**1** at PBE0-D3BJ/def2-SVP level of theory:

Ga	1.66126200	-0.01040300	0.98038700
Ga	-1.72494900	0.06939900	-0.55188900
P	-0.54504800	0.04225100	1.35641500
P	-0.84651900	0.16797900	-2.88454100
O	1.55216900	-0.27447500	-1.48644400
C	0.54759800	-0.08543800	-2.08460700
N	2.97348300	-1.53503500	0.91680600
N	3.08510000	1.36943000	0.72946500
N	-3.11015500	-1.34565100	-0.85933900
N	-3.04743400	1.56249300	-0.72320500
C	4.15888700	-1.35017300	1.48752100
C	4.69158700	-0.08257100	1.79702800
H	5.65432600	-0.09153200	2.30785200
C	4.26455500	1.16536700	1.31865300
C	5.04590400	-2.51738000	1.83782800
H	5.41342400	-2.39056600	2.86682000
H	5.93038400	-2.53337300	1.18556900
H	4.53571100	-3.48281800	1.74975900
C	5.20460100	2.32401500	1.51543300
H	6.14559200	1.99336000	1.97048900
H	4.74000400	3.08434500	2.16006400
H	5.42376900	2.82916300	0.56545200
C	2.66337700	-2.83070900	0.40095600
C	1.78763400	-3.67480500	1.11877100
C	1.59090700	-4.97819000	0.65624300
H	0.91823100	-5.64131800	1.20241400
C	2.23816500	-5.44353500	-0.48267800
H	2.08280500	-6.47081100	-0.82114800
C	3.05853900	-4.58709300	-1.20472200
H	3.53782300	-4.94534900	-2.11918900
C	3.27698300	-3.26844100	-0.79395100
C	1.11853400	-3.20727400	2.39542000
H	0.94324100	-2.12689100	2.27582500
C	2.00830100	-3.44121500	3.61681900
H	2.98444500	-2.94103700	3.52714700
H	2.20834200	-4.51552800	3.75916100
H	1.51551900	-3.07546900	4.53332100
C	-0.26827500	-3.79490100	2.60038100
H	-0.75375800	-3.31512300	3.46348100
H	-0.24994200	-4.87933100	2.79388200
H	-0.90057900	-3.60656200	1.72347200
C	4.15245300	-2.37119300	-1.65195200
H	4.15932400	-1.37299200	-1.19064600
C	5.59589400	-2.87078800	-1.73729400
H	6.21572000	-2.15741100	-2.30284800
H	5.64968800	-3.84026700	-2.25791000
H	6.05369700	-3.00549300	-0.74823200
C	3.57709200	-2.22306300	-3.06085700

H	4.18766800	-1.52126800	-3.65014300
H	2.54894800	-1.84432900	-3.03301300
H	3.57499300	-3.18636400	-3.59492100
C	2.87723100	2.64300200	0.11064800
C	2.31633500	3.70735100	0.84655100
C	2.24922000	4.96932300	0.24843900
H	1.81478100	5.80085500	0.80855800
C	2.73389500	5.18525800	-1.03453300
H	2.68859700	6.18247900	-1.47887100
C	3.25328300	4.12016500	-1.76087600
H	3.60878400	4.28695800	-2.78014400
C	3.32247400	2.83509100	-1.21667300
C	1.80402600	3.53808300	2.26303000
H	2.04582300	2.51097800	2.56888100
C	2.48456000	4.49111800	3.24712800
H	3.58224300	4.41061400	3.22158400
H	2.15260700	4.28137400	4.27620600
H	2.22795400	5.53994400	3.03151600
C	0.28702400	3.69560100	2.31776000
H	-0.08069300	3.56162200	3.34816000
H	-0.21511000	2.94837300	1.68539800
H	-0.01956600	4.69765200	1.98348200
C	3.88323600	1.69758000	-2.05093500
H	3.60038700	0.76497200	-1.54721900
C	5.40959800	1.73246400	-2.14011100
H	5.76409500	2.67864200	-2.58052300
H	5.77361000	0.90954800	-2.77603900
H	5.88411300	1.61895300	-1.15493000
C	3.26371900	1.66652800	-3.44716200
H	3.60128400	2.51419400	-4.06467600
H	2.16588000	1.69879700	-3.40255100
H	3.55384500	0.74453200	-3.97255800
C	-4.16103800	-1.09145200	-1.63588500
C	-4.55042900	0.19872200	-2.02799000
H	-5.41494600	0.24896000	-2.68758700
C	-4.12323100	1.42642200	-1.49236800
C	-5.03339700	-2.22878300	-2.08984000
H	-5.47585500	-2.74532900	-1.22561500
H	-4.44198900	-2.98253900	-2.62761600
H	-5.83931000	-1.87012400	-2.74053400
C	-4.97821500	2.62637500	-1.79462800
H	-5.24754300	3.15573400	-0.86940400
H	-5.89324200	2.33173100	-2.32136700
H	-4.42871500	3.34810500	-2.41483100
C	-2.96914700	-2.66788000	-0.33569600
C	-3.57525400	-2.97443100	0.90093200
C	-3.51426800	-4.28952900	1.37004600
H	-3.98934500	-4.53829400	2.32244400
C	-2.85206800	-5.27986600	0.65475200
H	-2.80974600	-6.30160900	1.03983900
C	-2.23111000	-4.95762600	-0.54560400
H	-1.69658000	-5.73121000	-1.10220600
C	-2.28471100	-3.66114300	-1.06700600
C	-4.29670700	-1.92588700	1.72370600
H	-4.20733300	-0.97042600	1.18719800
C	-5.78652100	-2.23964100	1.86011800
H	-6.30136500	-1.44189900	2.41806900
H	-5.95197000	-3.18457100	2.40203300
H	-6.27485400	-2.32898400	0.87793100
C	-3.63259700	-1.75019700	3.08794600
H	-2.58712400	-1.42533700	2.96978700
H	-3.64392400	-2.68879300	3.66502800
H	-4.16702200	-0.99223200	3.68095000
C	-1.60786300	-3.36183000	-2.38722400
H	-1.88468700	-2.33789200	-2.67841000
C	-2.04768900	-4.30145700	-3.50874600
H	-1.59709200	-3.98802000	-4.46322000
H	-3.14077000	-4.31027700	-3.63986600
H	-1.72935300	-5.33920700	-3.32095200
C	-0.09175300	-3.38716500	-2.22539500
H	0.23647300	-2.74628100	-1.39475000

H	0.40240800	-3.03693000	-3.14434300
H	0.26572800	-4.40480700	-2.01101000
C	-2.79834200	2.83556700	-0.12416400
C	-3.17773400	3.03724800	1.22043500
C	-3.05458900	4.31949300	1.76080300
H	-3.36054800	4.49666500	2.79328800
C	-2.54080700	5.37203400	1.01152400
H	-2.45765800	6.36905800	1.45120100
C	-2.10524500	5.14135500	-0.28628300
H	-1.66396900	5.95886300	-0.86138500
C	-2.22059300	3.87886400	-0.87665600
C	-3.73051300	1.90039500	2.05694100
H	-3.28436200	0.97537900	1.66051100
C	-5.24871000	1.78417900	1.91975800
H	-5.63321000	0.96270800	2.54454400
H	-5.54993900	1.57793800	0.88235900
H	-5.74602500	2.71393600	2.24064100
C	-3.30812700	1.99250000	3.51858200
H	-2.21378400	2.06717400	3.59767800
H	-3.62323800	1.08931000	4.06042400
H	-3.76163200	2.85443700	4.03326900
C	-1.67587500	3.66222100	-2.27342300
H	-2.01074200	2.67239100	-2.61782500
C	-2.15869000	4.70080400	-3.28446700
H	-1.76879900	5.70506800	-3.05509000
H	-3.25655600	4.77474100	-3.31936500
H	-1.80640100	4.43805800	-4.29386600
C	-0.15061500	3.63149100	-2.22664100
H	0.25000700	4.61349700	-1.93626400
H	0.26676700	3.36682300	-3.21017600
H	0.21582900	2.89680100	-1.49533400
N	1.95728000	0.08557900	3.19979700
H	2.00153600	-0.84943100	3.60206100
H	1.07826900	0.51202500	3.50008000
H	2.74815100	0.61724600	3.55936100

Cartesian coordinates of the optimized geometry of Int-**2** at PBE0-D3BJ/def2-SVP level of theory:

Ga	1.83302700	0.16468300	1.22652900
Ga	-1.84691500	0.02119000	-0.81846500
P	-0.25179300	-0.69955100	0.67100300
P	-1.42968000	0.45027200	-3.15060700
O	1.25209700	0.41098400	-2.29499300
C	0.13766200	0.42991100	-2.64321900
N	3.20326700	-1.28449400	1.02321800
N	3.10353100	1.57339100	0.61825900
N	-3.09251700	-1.52287800	-0.94073200
N	-3.27728900	1.34250900	-0.45992400
C	4.39635700	-0.99535300	1.51684500
C	4.81785100	0.32721200	1.78287600
H	5.77317900	0.42486400	2.29784400
C	4.27995600	1.50842000	1.25749100
C	5.40349800	-2.07361300	1.80633300
H	5.78176500	-1.94280000	2.83102000
H	6.26586300	-1.97724500	1.13059800
H	4.98461600	-3.08072500	1.70069100
C	5.07678600	2.76950900	1.45841300
H	6.05922900	2.54881400	1.89217700
H	4.53139900	3.44371100	2.13731600
H	5.21299100	3.32186600	0.51946800
C	2.91227200	-2.59366200	0.53764100
C	2.08947700	-3.45288100	1.30011100
C	1.80357500	-4.72110700	0.78867600
H	1.16045400	-5.39116800	1.36161100
C	2.32723300	-5.14776600	-0.42608100
H	2.09639800	-6.14740700	-0.80284500
C	3.14289200	-4.29619700	-1.15856400
H	3.55154800	-4.63113900	-2.11532500
C	3.44346000	-3.00843000	-0.70346700

C	1.59448500	-3.06729600	2.67943500
H	1.56803000	-1.96891100	2.75426900
C	2.57619700	-3.54137400	3.75360300
H	3.57809800	-3.11337800	3.60713000
H	2.67508800	-4.63954900	3.74726200
H	2.22695900	-3.23450000	4.75207900
C	0.18666300	-3.57317500	2.96913500
H	-0.18957000	-3.11899400	3.89895900
H	0.15080000	-4.66665100	3.10534600
H	-0.50025000	-3.30776200	2.15320200
C	4.32933400	-2.12214100	-1.56061000
H	4.44567600	-1.15796700	-1.04360600
C	5.72189400	-2.72643300	-1.75180600
H	6.37903000	-2.01986900	-2.28304800
H	5.67557300	-3.64905000	-2.35206200
H	6.19776100	-2.98252300	-0.79482500
C	3.68604400	-1.83695000	-2.91673400
H	4.33844700	-1.18764900	-3.52096800
H	2.71724400	-1.33528700	-2.80219900
H	3.52622600	-2.76644600	-3.48574400
C	2.78851900	2.79035100	-0.05608100
C	2.13544700	3.82830400	0.64033800
C	1.92388600	5.04417400	-0.01545700
H	1.41888500	5.85602300	0.51273600
C	2.34939600	5.23604200	-1.32309600
H	2.18263700	6.19458200	-1.82056300
C	2.97549800	4.19831400	-2.00450400
H	3.29354600	4.35434700	-3.03653600
C	3.20754500	2.96148300	-1.39510900
C	1.65486700	3.64475700	2.06511800
H	2.17357600	2.77023200	2.48535900
C	1.95857200	4.84212600	2.96187800
H	3.02301300	5.12178800	2.92534700
H	1.70434000	4.60750200	4.00720200
H	1.37142800	5.73000900	2.67910300
C	0.16423600	3.31569600	2.07891200
H	-0.19390900	3.15547400	3.10844300
H	-0.05873500	2.40765400	1.49819400
H	-0.42815500	4.13264400	1.64274700
C	3.91081000	1.85105000	-2.15751600
H	3.50273200	0.90703400	-1.77131300
C	5.42300400	1.82685200	-1.91850700
H	5.88289400	2.79521900	-2.17492500
H	5.89083800	1.05606100	-2.55191000
H	5.67952200	1.58754400	-0.87813100
C	3.62974300	1.90445700	-3.65670400
H	4.15183100	2.74471000	-4.14211600
H	2.55532000	2.00012600	-3.86437800
H	3.98592400	0.98302200	-4.13988700
C	-4.25710400	-1.32079700	-1.54644300
C	-4.81479900	-0.04133500	-1.72361900
H	-5.75811900	-0.00974400	-2.26611200
C	-4.43910300	1.15845200	-1.09311700
C	-5.03960800	-2.50036100	-2.04601500
H	-5.28561000	-3.18561600	-1.22294700
H	-4.42951800	-3.07914500	-2.75536800
H	-5.96643200	-2.18393300	-2.53835000
C	-5.43741500	2.28175500	-1.13572700
H	-5.61955800	2.68433400	-0.12918700
H	-6.38686700	1.94420600	-1.56651800
H	-5.05124400	3.11699000	-1.73736400
C	-2.73071900	-2.84266700	-0.52503500
C	-3.18994500	-3.30703900	0.72642800
C	-2.84715700	-4.60134400	1.12551400
H	-3.19952800	-4.97330700	2.09072500
C	-2.05213300	-5.41530800	0.32841000
H	-1.78413200	-6.42010000	0.66369000
C	-1.59194900	-4.93807200	-0.89145800
H	-0.95382200	-5.57052500	-1.51292600
C	-1.92571800	-3.65879000	-1.34441900
C	-4.03485100	-2.44963900	1.64956700

H	-4.10240800	-1.44808800	1.19997000
C	-5.45858100	-2.99035800	1.78715100
H	-6.06331700	-2.33244100	2.43104200
H	-5.46143500	-3.99520000	2.23882800
H	-5.96614500	-3.06160500	0.81409900
C	-3.37661600	-2.29998600	3.01959200
H	-2.37597000	-1.85374100	2.93375500
H	-3.26380400	-3.27258700	3.52294800
H	-3.98930200	-1.66273400	3.67485400
C	-1.39545800	-3.18517000	-2.67938100
H	-1.84983700	-2.20649600	-2.89213200
C	-1.77139800	-4.12207000	-3.82581100
H	-1.43907600	-3.69957800	-4.78673700
H	-2.85925200	-4.28226900	-3.88493000
H	-1.29715100	-5.11049500	-3.71824800
C	0.11521000	-2.97506900	-2.61274400
H	0.39682600	-2.33940000	-1.76045100
H	0.48305400	-2.50085600	-3.53548300
H	0.64642400	-3.92984400	-2.48124500
C	-3.08119400	2.56912900	0.25053000
C	-3.31066000	2.58768800	1.64248400
C	-3.27400800	3.81576100	2.30830600
H	-3.47023200	3.84977500	3.38144900
C	-2.98753100	4.99406200	1.62904500
H	-2.97034600	5.94594400	2.16506400
C	-2.68833100	4.94916600	0.27347100
H	-2.42086700	5.86951500	-0.25070500
C	-2.72073600	3.74634500	-0.43913500
C	-3.62568200	1.31496600	2.40369200
H	-3.18566300	0.48446900	1.83006000
C	-5.13322400	1.06872200	2.47811600
H	-5.35298500	0.15594200	3.05301700
H	-5.57435800	0.94392200	1.47861200
H	-5.64321000	1.91089200	2.97274500
C	-2.99115100	1.29596700	3.79125100
H	-1.91332700	1.51004200	3.73786100
H	-3.11651100	0.30839900	4.25633200
H	-3.45058000	2.03345300	4.46761000
C	-2.31915000	3.73791600	-1.89918100
H	-2.59275800	2.75950700	-2.32125800
C	-3.01365500	4.81495100	-2.72992200
H	-2.69913600	5.82820100	-2.43424900
H	-4.11005800	4.77036400	-2.63966500
H	-2.75813000	4.69289400	-3.79354700
C	-0.80086400	3.86546500	-2.01412300
H	-0.46225100	4.85000200	-1.65878700
H	-0.47622800	3.74618500	-3.05898600
H	-0.27703600	3.10714000	-1.41379000
N	1.71888500	0.31676800	3.09750700
H	2.56540900	0.57843100	3.59538900
H	-0.87700800	-0.18321500	1.84412700
H	0.94221300	0.85057500	3.47414500

Cartesian coordinates of the optimized geometry of Int-**3** at PBE0-D3BJ/def2-SVP level of theory:

Ga	-1.96247500	-0.14327300	-0.64570800
P	-2.72113000	-0.71603500	-2.86430800
O	-0.13502500	-0.95973000	-3.94649900
N	1.95174300	0.79450900	-2.50965000
H	1.29290400	0.35958000	-3.14861900
C	-1.18914200	-0.85290800	-3.45353700
C	-4.50445800	-1.14137900	0.17419600
N	-3.19895900	-1.34089800	0.34787300
P	0.03958100	-0.12089100	0.51395900
H	0.10871400	-1.54753000	0.50607800
Ga	1.98067400	0.17962800	-0.75486500
C	-5.04613700	0.05809000	-0.31626800
H	-6.12781900	0.06934300	-0.43901900
N	-3.08923600	1.47150400	-0.42240600

N	3.26364800	1.40524100	0.15161900
C	-4.40907000	1.30848300	-0.44652600
N	3.17914700	-1.41682300	-0.52468500
C	-5.46448700	-2.23845100	0.53522500
H	-5.24203100	-3.13979600	-0.05528700
H	-6.50140600	-1.93469200	0.35158800
H	-5.35605700	-2.52689900	1.58998000
C	-1.96072200	-4.67042200	2.63105200
H	-1.64524800	-5.52984100	3.22735500
C	-2.02945400	-4.76618100	1.24725000
H	-1.76663100	-5.70636400	0.75692300
C	-2.44446600	-3.68455800	0.46402100
C	-2.76204500	-2.46991800	1.10616700
C	-5.30035700	2.50737300	-0.60776500
H	-6.34284900	2.25371400	-0.38366800
H	-5.24441900	2.88092600	-1.64085900
H	-4.97751200	3.33245000	0.04181200
C	-2.67538100	-2.35186700	2.51045400
C	-2.28658300	-3.47064300	3.25198900
H	-2.22455400	-3.39369100	4.34026400
C	-2.54513200	-3.84684400	-1.03659300
H	-3.04471300	-2.95259800	-1.43756200
C	-3.38215800	-5.05875300	-1.44171700
H	-2.89807800	-6.00666200	-1.15809000
H	-3.52044400	-5.07451000	-2.53378200
H	-4.37898700	-5.04352900	-0.97425400
C	-3.00099300	-1.05952600	3.23317500
H	-3.18687200	-0.29328500	2.46683700
C	-1.15921900	-3.90357500	-1.67042100
H	-0.55232800	-3.02517400	-1.40467100
H	-1.23963600	-3.93818900	-2.76767800
H	-0.60230100	-4.79119600	-1.33558800
C	-4.26686400	-1.18493900	4.08140000
H	-4.14373700	-1.93929700	4.87497500
H	-5.13879400	-1.47571600	3.47725800
H	-4.50328300	-0.22504600	4.56669700
C	-1.81843700	-0.58987500	4.07592400
H	-1.55830800	-1.32163400	4.85679700
H	-2.05636600	0.35817200	4.58129900
H	-0.93194000	-0.43137600	3.44599800
C	-2.54554100	2.79411400	-0.42610600
C	-2.45328600	3.52967400	-1.62741300
C	-2.00432300	4.85159700	-1.55873500
H	-1.93451500	5.43691700	-2.47846800
C	-1.62354400	5.42320200	-0.35268100
H	-1.26895100	6.45634700	-0.32219700
C	-1.66744700	4.66807100	0.81301400
H	-1.33592600	5.11292000	1.75182500
C	-2.13051100	3.34956700	0.80394400
C	-2.77498100	2.92871900	-2.98132400
H	-3.25912800	1.95453100	-2.81513700
C	-3.72544900	3.79054200	-3.81104100
H	-4.64799900	4.03978400	-3.26465800
H	-4.01035600	3.26016100	-4.73261200
H	-3.25714700	4.74050700	-4.11310600
C	-1.48926300	2.65118500	-3.76122700
H	-0.77870300	2.04441700	-3.18218400
H	-0.97813400	3.59124900	-4.02319100
H	-1.71448400	2.11017400	-4.69320500
C	-2.22535700	2.55718100	2.09420600
H	-2.03137300	1.50415600	1.83580000
C	-3.62716100	2.63690400	2.70209200
H	-3.66392200	2.08932000	3.65686800
H	-4.39152000	2.19966000	2.04458800
H	-3.90733600	3.68340400	2.90375500
C	-1.17000900	2.96710500	3.11204300
H	-1.17328900	2.27192500	3.96298800
H	-1.35453800	3.97481500	3.51758700
H	-0.16460800	2.95593400	2.67105300
C	4.57403200	1.19803000	0.03270800
C	5.14104500	-0.01926600	-0.37184500

H	6.22782500	-0.03893300	-0.43318500
C	4.49978800	-1.26691800	-0.47868800
C	5.52513300	2.30594000	0.39030700
H	5.40262500	2.60713000	1.43987100
H	5.31071100	3.20029400	-0.21133600
H	6.56507500	2.00095000	0.22623600
C	5.40002600	-2.47606800	-0.46622500
H	5.42221600	-2.91003700	0.54439000
H	6.42476900	-2.19257400	-0.73653200
H	5.05034700	-3.26970400	-1.13549900
C	2.82642800	2.56364300	0.86298700
C	2.53898000	3.76164700	0.17733800
C	2.13304300	4.87359300	0.92240000
H	1.89591600	5.80389000	0.40043800
C	2.04127500	4.82118000	2.30713100
H	1.73308700	5.70428800	2.87214500
C	2.34078800	3.63677600	2.97114300
H	2.26540600	3.59625200	4.06080600
C	2.72460900	2.49078600	2.27043900
C	2.67376100	3.88091900	-1.32659800
H	3.04662500	2.91929000	-1.70335900
C	3.65681300	4.97824600	-1.73486500
H	4.65143600	4.83782600	-1.28464000
H	3.29888300	5.97566400	-1.43351600
H	3.78174800	4.99098600	-2.82892300
C	1.31459400	4.10385800	-1.97842700
H	0.59489600	3.33807300	-1.66286400
H	1.39953400	4.04732200	-3.07405700
H	0.89919500	5.08794800	-1.71283000
C	3.03973400	1.21898100	3.03693500
H	3.25610300	0.43539500	2.29620800
C	4.27876200	1.37577400	3.91925400
H	4.51072100	0.42742600	4.42898600
H	4.12340800	2.14242900	4.69518900
H	5.16548800	1.66588500	3.33693600
C	1.84089100	0.75239100	3.85828700
H	2.07650200	-0.18403900	4.38703700
H	0.97378700	0.57256400	3.20735700
H	1.55262100	1.49622600	4.61784500
C	2.67037400	-2.74846700	-0.50350200
C	2.51976500	-3.45430200	-1.71894800
C	2.20579600	-4.81410500	-1.64452100
H	2.10662300	-5.39167900	-2.56489300
C	1.98961800	-5.44103300	-0.42131900
H	1.74679200	-6.50602500	-0.38908400
C	2.03406600	-4.70181300	0.75374100
H	1.80368300	-5.18720600	1.70388500
C	2.37116100	-3.34398800	0.73870300
C	2.66106700	-2.76176700	-3.06793700
H	2.28511100	-1.73532000	-2.93106900
C	4.10356800	-2.63819600	-3.56697000
H	4.70921500	-1.96151500	-2.95046900
H	4.10641500	-2.23166000	-4.59033600
H	4.60276800	-3.62055200	-3.59822900
C	1.80866600	-3.41903100	-4.14990300
H	2.22182100	-4.39158600	-4.46335700
H	1.77544400	-2.77383600	-5.03944700
H	0.77427800	-3.57305400	-3.81575100
C	2.39367700	-2.54255000	2.03028900
H	2.23476200	-1.48894600	1.75185300
C	3.73548900	-2.61271900	2.76082100
H	3.66607300	-2.10014700	3.73312800
H	4.54034400	-2.12424800	2.19495200
H	4.03049800	-3.65713600	2.95206600
C	1.25342200	-2.93792500	2.96264000
H	1.39947400	-3.94132300	3.39302200
H	0.28544700	-2.93391000	2.44395200
H	1.18659000	-2.23121800	3.80248000
H	2.85887200	0.82794500	-2.96920900

Cartesian coordinates of the optimized geometry of Int-**4** at PBE0-D3BJ/def2-SVP level of theory:

Ga	-0.45023100	1.69280200	-0.53189400
P	-0.95795400	1.03357900	-2.67656300
O	-0.06903000	-1.19029500	-3.92808500
N	1.03196500	-0.91433200	-1.96193300
H	1.63079900	-0.12816400	-1.69925400
C	-0.01166000	-0.44888700	-2.96077800
C	-1.96143800	4.13971800	0.02321100
N	-1.92484800	2.82536200	0.23416800
P	0.05140800	0.11164300	1.18556300
H	-1.34714300	-0.14474000	1.32773700
Ga	0.36921900	-1.67779400	-0.20160000
C	-0.88025100	4.88720700	-0.45730300
H	-1.06852000	5.95130200	-0.59829700
N	0.87451400	3.23194500	-0.51447700
N	1.95404000	-2.79541000	0.20860800
C	0.45985800	4.48863700	-0.59907900
N	-0.88966300	-3.14366600	-0.54940200
C	-3.22273400	4.91273000	0.30321700
H	-3.83806700	4.92892400	-0.60973600
H	-2.99321000	5.95189300	0.56971500
H	-3.82617600	4.45618700	1.09720700
C	-4.97893500	0.91660300	2.43184000
H	-5.75977200	0.41209000	3.00637900
C	-5.12835400	1.10480700	1.06531300
H	-6.03264800	0.74719700	0.56709300
C	-4.14663800	1.75188700	0.30602800
C	-2.97898900	2.19431500	0.95960800
C	1.43175200	5.61611900	-0.83724100
H	1.23799300	6.43345000	-0.12905100
H	1.27046500	6.02083000	-1.84781400
H	2.47900600	5.30673100	-0.74980700
C	-2.81603100	2.01753500	2.35498900
C	-3.83164100	1.37902400	3.06970700
H	-3.72303100	1.23935100	4.14690300
C	-4.39030500	1.97826000	-1.17252900
H	-3.51093300	2.49825800	-1.58206400
C	-5.62794200	2.84547500	-1.41507100
H	-6.54632000	2.31917500	-1.10908300
H	-5.72548300	3.07985800	-2.48656300
H	-5.59480300	3.79304400	-0.85863900
C	-1.59271300	2.55808900	3.07260100
H	-0.77122800	2.55402600	2.33989300
C	-4.51811900	0.65779200	-1.92033900
H	-3.64247200	0.02245700	-1.74122800
H	-4.58237900	0.83302900	-3.00470200
H	-5.41876400	0.10709600	-1.60635700
C	-1.80033600	4.00944100	3.51057700
H	-2.65063400	4.09033400	4.20697200
H	-1.99738400	4.67279700	2.65801800
H	-0.90257300	4.38832000	4.02433800
C	-1.15366300	1.70297500	4.25607700
H	-1.87746600	1.74037400	5.08571400
H	-0.19457700	2.07247400	4.64958300
H	-1.01214000	0.65305600	3.96282100
C	2.26607000	2.93518100	-0.56656400
C	2.95165100	2.86357900	-1.80217100
C	4.33202400	2.63199200	-1.77444500
H	4.88045400	2.59568800	-2.71912200
C	5.01477200	2.43986000	-0.58106600
H	6.09202000	2.25546500	-0.58797000
C	4.32126100	2.47899200	0.62335000
H	4.85876800	2.32196000	1.55999800
C	2.94937900	2.73758000	0.65651900
C	2.27483400	3.08417000	-3.14462300
H	1.19359200	3.18872400	-2.96473900
C	2.79253800	4.34847700	-3.83602900
H	2.71807900	5.23795200	-3.19719300
H	2.22006600	4.54065700	-4.75663700

H	3.85099800	4.23802200	-4.12245400
C	2.45086600	1.89153000	-4.08273700
H	2.04152700	0.96916100	-3.65438100
H	3.51108900	1.72246400	-4.33203800
H	1.90573900	2.06588900	-5.02224300
C	2.23037800	2.90116400	1.98130000
H	1.17296200	2.66235100	1.79853300
C	2.29523600	4.35072600	2.46526400
H	1.75279700	4.46265400	3.41751200
H	1.84581800	5.04513900	1.74292700
H	3.33906600	4.66374200	2.62980300
C	2.72860000	1.94660500	3.05684500
H	2.08730900	2.01552100	3.94845900
H	3.75655100	2.17988600	3.37822700
H	2.70519800	0.90779000	2.70309800
C	1.98494000	-4.09126200	-0.09298800
C	0.88972100	-4.79498700	-0.60751700
H	1.07091400	-5.85028200	-0.80929800
C	-0.45185900	-4.39481900	-0.70563400
C	3.23525500	-4.89844800	0.12697100
H	3.94063400	-4.40526100	0.80550000
H	3.74016100	-5.05206500	-0.83918100
H	2.98214900	-5.89066500	0.52268900
C	-1.41995900	-5.51317800	-0.97444200
H	-1.10330700	-6.42125100	-0.44581000
H	-1.41297600	-5.73785500	-2.05131200
H	-2.44672400	-5.26214000	-0.68722900
C	3.06552700	-2.17024900	0.85528200
C	4.18605100	-1.75002900	0.10774500
C	5.22981100	-1.10703400	0.78100700
H	6.09835200	-0.76435700	0.21374300
C	5.18506800	-0.90590200	2.15333300
H	6.01171600	-0.40420400	2.66175600
C	4.08589100	-1.34931200	2.88079000
H	4.06133600	-1.19708000	3.96118500
C	3.00867000	-1.97929500	2.25462400
C	4.32194300	-2.00522600	-1.38217100
H	3.41762700	-2.54168400	-1.70927700
C	5.52806500	-2.89217400	-1.70074600
H	5.51734100	-3.83196100	-1.13243600
H	6.46931300	-2.37374900	-1.46052000
H	5.55231800	-3.13863600	-2.77366500
C	4.41820300	-0.70514400	-2.17647200
H	3.64986900	0.02321000	-1.88879700
H	4.33211800	-0.89290600	-3.25801400
H	5.38377900	-0.20798500	-2.00087000
C	1.85400900	-2.52245800	3.07455200
H	0.98025200	-2.56751200	2.40770200
C	2.14883100	-3.95082500	3.53747900
H	1.30083400	-4.35451400	4.11253600
H	3.04032500	-3.97586300	4.18451700
H	2.33087100	-4.62787600	2.69112600
C	1.46997400	-1.63365400	4.25121800
H	0.57131400	-2.03404300	4.74462700
H	1.24451300	-0.61250300	3.91308900
H	2.26164400	-1.58911900	5.01561700
C	-2.30389900	-2.89960900	-0.44167900
C	-3.13498200	-2.86019600	-1.58268300
C	-4.50739800	-2.67302800	-1.38099200
H	-5.16565700	-2.64485400	-2.25193800
C	-5.04466000	-2.50206600	-0.11470000
H	-6.11811300	-2.34093700	0.00971400
C	-4.21084200	-2.53011400	0.99621700
H	-4.63659200	-2.39219200	1.99057600
C	-2.83851900	-2.74652100	0.86019800
C	-2.64634600	-3.04650300	-3.00954600
H	-1.54921700	-3.13211500	-2.99414100
C	-3.23849300	-4.30818400	-3.64871200
H	-3.09898500	-5.21328100	-3.04324700
H	-2.78103400	-4.48061000	-4.63510300
H	-4.32201100	-4.18984200	-3.80862400

C	-2.99001300	-1.84732000	-3.89328900
H	-4.08116100	-1.74685100	-4.01291500
H	-2.54410800	-1.97890900	-4.88888200
H	-2.59629600	-0.90768100	-3.48490800
C	-1.97493300	-2.90393800	2.09919500
H	-0.97232100	-2.51772200	1.85747800
C	-1.81020000	-4.37747100	2.47683300
H	-1.18668400	-4.47594300	3.37949000
H	-1.33059100	-4.96059700	1.67898700
H	-2.78962600	-4.83509100	2.68837800
C	-2.47189700	-2.09937900	3.29418300
H	-3.39671400	-2.52206400	3.71760500
H	-2.67003300	-1.05248300	3.02530500
H	-1.71659900	-2.10974300	4.09401800
H	1.60197200	-1.58924400	-2.47890300

Cartesian coordinates of the optimized geometry of Int-**5** at PBE0-D3BJ/def2-SVP level of theory:

Ga	1.08953900	1.35775100	-0.49450700
P	0.65191600	1.22096700	-2.75801100
O	-0.08466900	-0.69696600	-4.32813800
N	-0.64162800	-1.27437400	-2.20586000
H	-0.79276800	-2.16354100	-2.68056500
C	-0.07341100	-0.35519900	-3.01142200
C	1.83793300	4.16022600	-0.02787900
N	0.88286900	3.24839700	0.16091200
P	-0.03691400	0.04605500	1.11993400
H	-1.10223100	1.00165700	1.10513800
Ga	-1.12784700	-1.33823800	-0.34834900
C	3.15557800	3.84644400	-0.36556200
H	3.82965200	4.69354500	-0.48715500
N	3.11869300	1.42863000	-0.31159200
N	-0.90427500	-3.25375700	0.09263100
C	3.77149500	2.58080600	-0.36705000
N	-3.11908700	-1.44876200	-0.40963400
C	1.51375300	5.62019500	0.14068100
H	0.92489500	5.96554300	-0.72236000
H	2.42858800	6.22181100	0.19676400
H	0.90226700	5.80487000	1.03379200
C	-2.64869900	4.45560500	2.12039400
H	-3.55912500	4.77046400	2.63650300
C	-2.55659500	4.56200500	0.73923600
H	-3.40092100	4.95975300	0.17099500
C	-1.39759700	4.17706700	0.05681800
C	-0.32353000	3.65313200	0.80218000
C	5.27754200	2.62344200	-0.40835400
H	5.64597400	3.37087600	0.30760000
H	5.60310100	2.94517300	-1.40890900
H	5.74035800	1.65537400	-0.18873800
C	-0.40586800	3.52837300	2.20909900
C	-1.57646200	3.94539800	2.84648500
H	-1.65231800	3.86661500	3.93300400
C	-1.32895600	4.33309200	-1.44817600
H	-0.31170800	4.05847500	-1.76620000
C	-1.60603200	5.76738300	-1.89967900
H	-2.64696600	6.06067100	-1.68930000
H	-1.45220800	5.86144400	-2.98583900
H	-0.95384600	6.49820600	-1.39857400
C	0.76702800	3.00306700	3.01691300
H	1.36696400	2.38370700	2.33324500
C	-2.28551600	3.36611900	-2.13274300
H	-2.11192900	2.33728200	-1.79154100
H	-2.13801800	3.38304000	-3.22278100
H	-3.33328200	3.62411400	-1.91554300
C	1.66567700	4.14352600	3.49981500
H	1.10412600	4.84116700	4.14226000
H	2.08642500	4.71714800	2.66274500
H	2.50974000	3.74679600	4.08564600
C	0.34734200	2.11744600	4.18529900

H	-0.19762000	2.68032500	4.95977200
H	1.23827600	1.68688100	4.66751400
H	-0.28575100	1.28493900	3.84678500
C	3.85257800	0.20584500	-0.27192500
C	4.46849500	-0.31361300	-1.43364100
C	5.20201400	-1.49947700	-1.31194700
H	5.69645700	-1.90683900	-2.19749200
C	5.31087200	-2.16883000	-0.10076700
H	5.88420100	-3.09705100	-0.03537300
C	4.69126000	-1.64963100	1.03064300
H	4.78228000	-2.17353100	1.98325200
C	3.97044900	-0.45550200	0.97213200
C	4.42687300	0.37425500	-2.78852600
H	3.83824500	1.29841100	-2.68313600
C	5.83407000	0.73588600	-3.27528200
H	6.41590900	1.28509800	-2.52273800
H	5.77531500	1.35598400	-4.18330700
H	6.40723200	-0.16855600	-3.53492100
C	3.72645300	-0.47286800	-3.84926700
H	2.67354100	-0.63773600	-3.59258000
H	4.22518100	-1.44691800	-3.97986000
H	3.74416900	0.04891300	-4.81850600
C	3.41766500	0.17348700	2.23603200
H	2.53363600	0.75727700	1.94240600
C	4.42639700	1.14831600	2.84595700
H	4.01300300	1.60949800	3.75707600
H	4.68558700	1.95920300	2.15166200
H	5.35804500	0.62801000	3.12125500
C	2.95285900	-0.84463500	3.26604000
H	2.43311900	-0.33533600	4.09121100
H	3.79282400	-1.40519600	3.70744500
H	2.25483600	-1.56527200	2.82105800
C	-1.82699800	-4.17351700	-0.18866200
C	-3.13131800	-3.86143600	-0.58532600
H	-3.78608700	-4.70961600	-0.78067300
C	-3.75842100	-2.60203800	-0.56959400
C	-1.47667900	-5.62990200	-0.05846100
H	-1.00613500	-5.84381700	0.91080800
H	-0.74047400	-5.90929000	-0.82733000
H	-2.36504700	-6.26107600	-0.17686700
C	-5.25466400	-2.63054800	-0.73529900
H	-5.67438700	-3.49622000	-0.20653200
H	-5.50361100	-2.74473700	-1.80118400
H	-5.73529200	-1.71412900	-0.37407200
C	0.28454000	-3.66092700	0.77000100
C	1.39833900	-4.11336100	0.03749100
C	2.53510900	-4.51683600	0.74307200
H	3.41480900	-4.85581900	0.19187700
C	2.56044500	-4.49773500	2.13209900
H	3.45462900	-4.82575100	2.66758300
C	1.44955400	-4.05284400	2.84104500
H	1.48017500	-4.03586500	3.93264700
C	0.29899200	-3.61381700	2.18112400
C	1.37933200	-4.16225900	-1.47651600
H	0.33274000	-4.02447700	-1.78862100
C	1.85370100	-5.50490400	-2.02986800
H	1.29561100	-6.35147900	-1.60076200
H	2.92130500	-5.67306400	-1.81983400
H	1.73144200	-5.53291800	-3.12377400
C	2.18915000	-3.01465900	-2.06928500
H	1.88710500	-2.04361700	-1.65386900
H	2.06201900	-2.96583200	-3.16156600
H	3.25959600	-3.13923700	-1.85503700
C	-0.91579700	-3.15739500	2.96971500
H	-1.54858200	-2.58309300	2.27667100
C	-1.74160700	-4.35015200	3.45603300
H	-2.62342900	-4.00535300	4.01867200
H	-1.14671500	-4.99598500	4.12195800
H	-2.10267900	-4.96838300	2.62189900
C	-0.56247700	-2.22991300	4.12781400
H	-1.48247500	-1.87359300	4.61695000

H	-0.00626300	-1.35285900	3.76831500
H	0.03776900	-2.73754400	4.89936600
C	-3.85698400	-0.22730200	-0.37208300
C	-4.36496700	0.33539400	-1.56231800
C	-5.09349500	1.52498400	-1.46580500
H	-5.49961400	1.97486800	-2.37494100
C	-5.29541800	2.15151400	-0.24380200
H	-5.85679400	3.08752700	-0.19405400
C	-4.77479300	1.59076200	0.91752600
H	-4.93082900	2.09410500	1.87228000
C	-4.06057400	0.39154300	0.88172600
C	-4.16947900	-0.30342200	-2.92703700
H	-3.65997700	-1.26649400	-2.77278500
C	-5.50363800	-0.56974700	-3.62828300
H	-6.20768900	-1.12843800	-2.99544600
H	-5.34409700	-1.14296200	-4.55499000
H	-5.99673100	0.37319700	-3.91177900
C	-3.26909900	0.54084800	-3.82748400
H	-3.74218700	1.50671400	-4.06393600
H	-3.08600800	0.02447100	-4.78358100
H	-2.29887500	0.75675700	-3.36167300
C	-3.57765700	-0.26734400	2.16104000
H	-2.61776600	-0.75653400	1.93068000
C	-4.54123200	-1.36200300	2.62393800
H	-4.17721400	-1.82201900	3.55620600
H	-4.64643900	-2.16411500	1.88085900
H	-5.54218800	-0.94530200	2.81972000
C	-3.30847000	0.72047100	3.28812200
H	-4.23964600	1.15340000	3.68772800
H	-2.66385200	1.54593300	2.95650600
H	-2.80435600	0.20955600	4.12187500
H	-0.78754400	-1.34355300	-4.47998200

Cartesian coordinates of the optimized geometry of Int-**6** at PBE0-D3BJ/def2-SVP level of theory:

Ga	1.13386900	1.34039500	-0.49548100
P	0.71504200	1.17132900	-2.75472200
O	-0.10739100	-0.78523300	-4.29932200
N	-0.71177400	-1.26802600	-2.22711100
H	-1.03275300	-2.06095100	-2.78112000
C	-0.08134300	-0.38492900	-3.00478600
C	1.95476700	4.11711900	-0.02733000
N	0.97486700	3.23115300	0.15801800
P	-0.04341300	0.04538800	1.08746200
H	-1.08523000	1.02653500	1.07163100
Ga	-1.17661000	-1.32427100	-0.36799200
C	3.26445300	3.76723400	-0.36084800
H	3.96203000	4.59556100	-0.47945700
N	3.16093000	1.35127700	-0.30714400
N	-1.00750500	-3.23569500	0.09862800
C	3.84573500	2.48504200	-0.35890900
N	-3.16728200	-1.37272400	-0.41238100
C	1.66852800	5.58478200	0.14130000
H	1.08550700	5.94496600	-0.71968000
H	2.59842700	6.16305000	0.19501100
H	1.06430200	5.78447600	1.03614900
C	-2.52691300	4.52730200	2.11464200
H	-3.42967000	4.86471800	2.62989900
C	-2.43123000	4.63088000	0.73352500
H	-3.26526900	5.04864800	0.16452000
C	-1.28184100	4.21657000	0.05214900
C	-0.22196100	3.66618300	0.79852900
C	5.35253400	2.48604400	-0.39041300
H	5.73661400	3.21992000	0.33135000
H	5.69400900	2.80307800	-1.38715500
H	5.78660000	1.50464000	-0.17128900
C	-0.30836300	3.54370800	2.20520500
C	-1.46851500	3.99039800	2.84157500
H	-1.54714200	3.91359800	3.92805400

C	-1.20925800	4.36800500	-1.45300400
H	-0.19913100	4.06705500	-1.77002200
C	-1.45022800	5.80793800	-1.90713800
H	-2.48279000	6.12833500	-1.69532100
H	-1.29654600	5.89591700	-2.99386400
H	-0.77833800	6.52286700	-1.40901100
C	0.84988000	2.99032700	3.01525500
H	1.44428300	2.36762100	2.32998900
C	-2.18999500	3.42309200	-2.13402900
H	-2.04000200	2.39087600	-1.79220000
H	-2.04579800	3.43495100	-3.22458500
H	-3.23100700	3.70537200	-1.91487700
C	1.76358800	4.11125100	3.51534400
H	1.20958600	4.81041700	4.16264800
H	2.19701500	4.68786300	2.68670400
H	2.59849500	3.69531700	4.10092500
C	0.40641400	2.10041300	4.17154500
H	-0.14137500	2.66318900	4.94401200
H	1.28646900	1.65577900	4.66086100
H	-0.23219800	1.27828700	3.81837300
C	3.85911400	0.10733600	-0.25997200
C	4.46755900	-0.43388600	-1.41564600
C	5.16381900	-1.64115800	-1.28642700
H	5.65146700	-2.06612300	-2.16743800
C	5.24409900	-2.31038900	-0.07324000
H	5.78784100	-3.25572900	-0.00179700
C	4.63271100	-1.76956200	1.05225000
H	4.70040700	-2.29423700	2.00624900
C	3.94828100	-0.55465700	0.98620600
C	4.45718900	0.25012000	-2.77303300
H	3.88760300	1.18723900	-2.67749100
C	5.87720300	0.57679300	-3.24689600
H	6.46258900	1.11756000	-2.49125000
H	5.84319200	1.19261300	-4.15921800
H	6.43275700	-0.34195800	-3.49401400
C	3.74927400	-0.58662400	-3.83691600
H	2.69221800	-0.72829500	-3.58229000
H	4.22594200	-1.57239800	-3.95941100
H	3.79068500	-0.06948500	-4.80863100
C	3.40283300	0.09023200	2.24522300
H	2.53213300	0.69079200	1.94568200
C	4.42828600	1.04592400	2.85752600
H	4.02074500	1.51605500	3.76666000
H	4.70569000	1.85105100	2.16356500
H	5.34847800	0.50775500	3.13686800
C	2.91195100	-0.91485200	3.27604600
H	2.39692200	-0.39251700	4.09585100
H	3.73831700	-1.48930100	3.72515600
H	2.20325000	-1.62414700	2.82956200
C	-1.95577200	-4.13107600	-0.17485900
C	-3.25328600	-3.78502400	-0.56649800
H	-3.93172700	-4.61544800	-0.75636600
C	-3.84233500	-2.50783100	-0.56126800
C	-1.64310600	-5.59542900	-0.04221300
H	-1.16992100	-5.81930400	0.92348100
H	-0.92101200	-5.89433800	-0.81711800
H	-2.54841200	-6.20362600	-0.15211400
C	-5.33788600	-2.48646600	-0.72897000
H	-5.78392600	-3.37183300	-0.25834900
H	-5.58780200	-2.51940500	-1.80016000
H	-5.79230200	-1.58087100	-0.31000600
C	0.17457000	-3.67011800	0.76986100
C	1.27498100	-4.14473100	0.03135200
C	2.40469000	-4.57617500	0.73194300
H	3.27390600	-4.93435100	0.17612400
C	2.43592800	-4.56120700	2.12096300
H	3.32405800	-4.91199300	2.65217400
C	1.33884100	-4.09134600	2.83554500
H	1.37418500	-4.07715500	3.92714700
C	0.19631500	-3.62441700	2.18088700
C	1.24884900	-4.18834400	-1.48263900

H	0.20945300	-4.00632700	-1.79410500
C	1.67101000	-5.54701800	-2.03958600
H	1.08190900	-6.37272300	-1.61126100
H	2.73187700	-5.75670800	-1.83177600
H	1.54437500	-5.56769900	-3.13307200
C	2.10096200	-3.07210800	-2.07592700
H	1.83295800	-2.09021300	-1.66242300
H	1.97080600	-3.02179600	-3.16749000
H	3.16670500	-3.23493600	-1.86225600
C	-1.00343000	-3.13818000	2.97476600
H	-1.63253600	-2.56113800	2.28062100
C	-1.84520500	-4.31059200	3.48248700
H	-2.71774500	-3.94406600	4.04590900
H	-1.25656200	-4.95708300	4.15332700
H	-2.22118200	-4.93406300	2.65889400
C	-0.62310100	-2.20364000	4.11869100
H	-1.53178000	-1.83013000	4.61614800
H	-0.05975500	-1.33796300	3.74316100
H	-0.01905400	-2.71116800	4.88737000
C	-3.86972300	-0.13029700	-0.37620700
C	-4.36280800	0.44543800	-1.56647500
C	-5.05770400	1.65507700	-1.46952100
H	-5.45183400	2.11535500	-2.37872200
C	-5.24054400	2.28791300	-0.24768000
H	-5.77602900	3.23904400	-0.19782900
C	-4.73330700	1.71441700	0.91343300
H	-4.87332100	2.22311900	1.86787700
C	-4.05256800	0.49578500	0.87737600
C	-4.17837200	-0.19567000	-2.93129900
H	-3.70415700	-1.17627600	-2.77767200
C	-5.51302400	-0.41386600	-3.64760300
H	-6.24287500	-0.95184700	-3.02556500
H	-5.36082200	-0.98806900	-4.57469600
H	-5.97205700	0.54587700	-3.93224200
C	-3.23895200	0.61839500	-3.81970900
H	-3.68734200	1.59043000	-4.07942500
H	-3.03308000	0.07813200	-4.75642500
H	-2.27821300	0.81651800	-3.32864300
C	-3.58160000	-0.17275900	2.15640900
H	-2.63636300	-0.68825900	1.92275300
C	-4.57056900	-1.24067600	2.62807900
H	-4.21467100	-1.70497100	3.56139700
H	-4.69802200	-2.04381900	1.88962500
H	-5.56011700	-0.79820500	2.82536300
C	-3.28050600	0.81088400	3.27916500
H	-4.19780000	1.26983900	3.68182300
H	-2.61584000	1.61791000	2.94181900
H	-2.78612300	0.28932600	4.11215900
H	0.39531700	-0.12081000	-4.79738100

Cartesian coordinates of the optimized geometry of TS-**1a** at PBE0-D3BJ/def2-SVP level of theory:

Ga	1.78752500	0.19124700	0.99062800
Ga	-1.84517400	-0.02725100	-0.76345600
P	-0.29829300	-0.69497300	0.74867700
P	-1.41055200	0.50725700	-3.08823500
O	1.29391500	0.61300000	-2.31587200
C	0.16595000	0.56728100	-2.62161400
N	3.21864300	-1.19228200	1.09801100
N	3.03388100	1.68167900	0.66843000
N	-3.02133500	-1.61828100	-1.00468900
N	-3.35626700	1.22638400	-0.45036700
C	4.35441300	-0.84335600	1.68286100
C	4.68556500	0.49583100	1.98533500
H	5.59187100	0.63488300	2.57359500
C	4.14878800	1.65560700	1.41291700
C	5.39947900	-1.87323300	2.00857600
H	5.87094800	-1.63356300	2.97186300
H	6.19133400	-1.84768200	1.24451600

H	4.99169300	-2.89052100	2.03757900
C	4.88986000	2.94223700	1.65756700
H	5.83133400	2.75795200	2.18792700
H	4.26963900	3.62299000	2.26071400
H	5.10257900	3.47003100	0.71829900
C	3.03333600	-2.51940600	0.60211900
C	2.20947100	-3.42057500	1.31345500
C	2.03150900	-4.70560500	0.79732100
H	1.38624300	-5.40764300	1.32674600
C	2.65536500	-5.10492700	-0.37913900
H	2.50454800	-6.11655200	-0.76372500
C	3.46060100	-4.20870800	-1.06710400
H	3.93989900	-4.51971300	-1.99866100
C	3.65917000	-2.90482100	-0.60142000
C	1.57041700	-3.03464300	2.63201600
H	1.35307800	-1.96088300	2.57321900
C	2.52625200	-3.25207300	3.80632000
H	3.44515600	-2.65700400	3.70904500
H	2.81940300	-4.31153200	3.88413100
H	2.04197900	-2.96760700	4.75435500
C	0.23575900	-3.72373500	2.87721500
H	-0.25182600	-3.28887000	3.76293100
H	0.35058500	-4.80298900	3.06782800
H	-0.43597800	-3.58820800	2.01829200
C	4.51481600	-1.96165500	-1.42594900
H	4.59186900	-1.00727300	-0.88355300
C	5.93214900	-2.49897700	-1.62992800
H	6.55904500	-1.74765100	-2.13533300
H	5.93155200	-3.40303200	-2.25908300
H	6.41626000	-2.76349300	-0.67842500
C	3.85348000	-1.67050400	-2.77249600
H	4.48589000	-1.00078800	-3.37448200
H	2.87334000	-1.19241600	-2.64531200
H	3.70709400	-2.59695200	-3.34967600
C	2.73406100	2.87868700	-0.05506800
C	2.01095900	3.91269100	0.57359700
C	1.77620200	5.09361900	-0.13510500
H	1.21234500	5.89952400	0.33923100
C	2.24679100	5.25500200	-1.43062100
H	2.05545000	6.18374500	-1.97334500
C	2.95387200	4.22566700	-2.04085900
H	3.31111300	4.36005500	-3.06250000
C	3.21415900	3.02345800	-1.37611700
C	1.47091300	3.76159400	1.98062800
H	2.04433800	2.95341800	2.46051600
C	1.65641100	5.01331100	2.83513600
H	2.70042000	5.36342900	2.83036300
H	1.36943200	4.80732700	3.87790700
H	1.02370500	5.84428400	2.48702400
C	0.00314500	3.34200300	1.94018900
H	-0.40192800	3.20589400	2.95552700
H	-0.14815600	2.40158600	1.38745500
H	-0.60743800	4.10956400	1.44430700
C	4.00593600	1.92462200	-2.06466000
H	3.58664100	0.97278400	-1.71006500
C	5.49078900	1.94018900	-1.69152400
H	5.94876300	2.91594200	-1.92161200
H	6.03137300	1.17289600	-2.26860600
H	5.65977400	1.72188500	-0.62883700
C	3.85677100	1.95881500	-3.58244200
H	4.37600000	2.82314400	-4.02646700
H	2.80072700	1.99401600	-3.88243500
H	4.30322800	1.05715200	-4.02545400
C	-4.18150900	-1.45264300	-1.62777500
C	-4.80178600	-0.19830500	-1.77374200
H	-5.73876600	-0.19906400	-2.32824900
C	-4.49803000	0.99999800	-1.10220400
C	-4.90222300	-2.65319200	-2.17074200
H	-5.19753900	-3.33076400	-1.35683400
H	-4.23439300	-3.23159400	-2.82569500
H	-5.79781000	-2.35869400	-2.73035000

C	-5.56039200	2.06457400	-1.11748000
H	-5.76596300	2.42633700	-0.09979200
H	-6.48833700	1.68313200	-1.55860600
H	-5.22670200	2.93812200	-1.69503100
C	-2.61200300	-2.93262000	-0.62068600
C	-3.09799100	-3.46340700	0.59347100
C	-2.72576900	-4.76084700	0.95458400
H	-3.10171200	-5.18270600	1.89020700
C	-1.87094100	-5.51370200	0.15897200
H	-1.58254400	-6.52300000	0.46279000
C	-1.37504200	-4.96716100	-1.01725900
H	-0.68651300	-5.54859200	-1.63500400
C	-1.73770400	-3.68268200	-1.43267000
C	-3.98291100	-2.66414700	1.53042900
H	-4.11766900	-1.66615700	1.08845700
C	-5.36822300	-3.28907300	1.69270900
H	-6.00245500	-2.66277000	2.33977600
H	-5.30725100	-4.28831700	2.15269400
H	-5.88299500	-3.39899600	0.72663000
C	-3.29899300	-2.48050900	2.88418000
H	-2.33533700	-1.96270300	2.76836600
H	-3.10520800	-3.44919100	3.37085800
H	-3.93456400	-1.89289200	3.56372900
C	-1.16423900	-3.13311600	-2.71947100
H	-1.64878200	-2.16545500	-2.91519300
C	-1.44585200	-4.03468300	-3.91980600
H	-1.08777000	-3.55747800	-4.84527400
H	-2.52230000	-4.23503300	-4.03797100
H	-0.93559900	-5.00712400	-3.83105400
C	0.33079300	-2.86194500	-2.56612500
H	0.53011100	-2.22827300	-1.68861500
H	0.72486200	-2.35506100	-3.46038700
H	0.89593700	-3.79641100	-2.42348800
C	-3.22180600	2.44383400	0.28655900
C	-3.41422200	2.41304600	1.68394200
C	-3.43562900	3.62353800	2.38238100
H	-3.60773700	3.61606900	3.46033900
C	-3.24350500	4.83532000	1.72962100
H	-3.27527700	5.77258100	2.29035900
C	-2.97561700	4.84371800	0.36657900
H	-2.77862900	5.79230600	-0.13849400
C	-2.94553100	3.65948400	-0.37675200
C	-3.64161900	1.10522700	2.41521500
H	-3.17837400	0.31366100	1.80627800
C	-5.13552100	0.79672500	2.52468600
H	-5.30434300	-0.14784200	3.06417200
H	-5.60146800	0.69990000	1.53300200
H	-5.66207400	1.59659900	3.06995200
C	-2.95877400	1.07234200	3.77886700
H	-1.88519400	1.29339100	3.68680900
H	-3.05473400	0.07374500	4.22746300
H	-3.40274100	1.79002800	4.48650900
C	-2.55299000	3.71525200	-1.83883200
H	-2.75677900	2.72999500	-2.28416100
C	-3.32161100	4.76311100	-2.64114000
H	-3.07678700	5.78762200	-2.31908600
H	-4.41216200	4.64187000	-2.55196700
H	-3.06088400	4.68663500	-3.70780000
C	-1.04700900	3.95092700	-1.95260900
H	-0.77600500	4.94646900	-1.56849300
H	-0.71841300	3.88504900	-3.00095800
H	-0.47276400	3.20982000	-1.37826800
N	1.21856900	0.41279100	2.90664200
H	1.67773600	-0.13447300	3.63585900
H	-0.00052600	-0.04114900	2.24849600
H	1.11871600	1.36339200	3.25742500

Cartesian coordinates of the optimized geometry of TS-**1b** at PBE0-D3BJ/def2-SVP level of theory:

Ga	1.78086600	0.02006100	1.21812500
Ga	-1.74502400	0.05906500	-0.76066900
P	-0.45664200	-0.38107300	1.03169000
P	-0.98565100	0.13310500	-3.07148700
O	1.49978200	-0.42060600	-1.87200700
C	0.46551500	-0.18700800	-2.36663500
N	3.04146400	-1.53883300	0.93366500
N	3.15831300	1.36858600	0.66220500
N	-3.16263300	-1.35067800	-0.92442700
N	-3.07096300	1.54822700	-0.79237700
C	4.25478600	-1.35445200	1.43921700
C	4.81066600	-0.08058700	1.67866500
H	5.79082700	-0.07953300	2.15484400
C	4.36910800	1.15854200	1.19414600
C	5.11820600	-2.52092700	1.84669400
H	5.09791400	-2.58398000	2.94699300
H	6.16479600	-2.36329000	1.55558100
H	4.76602500	-3.47615100	1.44281300
C	5.33346400	2.30934800	1.31575300
H	6.28108300	1.97936600	1.75728600
H	4.89921300	3.10092700	1.94356300
H	5.53719300	2.77444800	0.34218600
C	2.68830400	-2.82613500	0.42887600
C	1.84435900	-3.67747600	1.17377800
C	1.60621800	-4.96900000	0.69483800
H	0.95907100	-5.63645400	1.26717200
C	2.18711700	-5.42085600	-0.48288600
H	2.00363300	-6.44024600	-0.83126700
C	2.98210000	-4.55937500	-1.22828300
H	3.41256300	-4.90592400	-2.17058000
C	3.23231700	-3.25080900	-0.80666100
C	1.26008500	-3.26178700	2.50861200
H	1.26975400	-2.16415100	2.53087800
C	2.11748600	-3.76554900	3.67005600
H	3.15353100	-3.40591200	3.59973200
H	2.14940800	-4.86698500	3.68568700
H	1.70448400	-3.42864800	4.63479600
C	-0.19566300	-3.67704700	2.66991700
H	-0.60862000	-3.25520400	3.59976700
H	-0.31675800	-4.77019000	2.72700200
H	-0.79895800	-3.29861500	1.83384900
C	4.08480800	-2.34548900	-1.67952900
H	3.95326600	-1.32072000	-1.30537000
C	5.57259800	-2.69333900	-1.60200600
H	6.15815400	-2.01758100	-2.24517500
H	5.75279000	-3.72442400	-1.94730800
H	5.97018600	-2.60996000	-0.58322300
C	3.63003300	-2.36797200	-3.13822900
H	4.17771100	-1.60752000	-3.71609400
H	2.55731700	-2.15724900	-3.22499900
H	3.83247100	-3.34103800	-3.61290900
C	2.96873000	2.62014900	-0.00076300
C	2.44896100	3.72822100	0.69853200
C	2.46463100	4.98083500	0.07564300
H	2.07863800	5.84891600	0.61621000
C	2.96815500	5.14268500	-1.20785200
H	2.98828300	6.13192200	-1.67141600
C	3.41542700	4.03033500	-1.91186200
H	3.77418300	4.15101700	-2.93634700
C	3.41315000	2.75738700	-1.33641400
C	1.87771700	3.61576800	2.09839900
H	1.99025500	2.57396900	2.42917200
C	2.61311400	4.49979900	3.10605500
H	3.69205800	4.28630400	3.13229200
H	2.21616000	4.33776500	4.12060700
H	2.49367400	5.56941500	2.87117000
C	0.38636500	3.94498900	2.08158100
H	-0.02741900	3.93378500	3.10370400
H	-0.18071900	3.23659900	1.45874300
H	0.20146400	4.95214500	1.68125400
C	3.88718500	1.56397500	-2.14470500

H	3.49151600	0.67275300	-1.64231400
C	5.41091800	1.43693800	-2.18526700
H	5.87890200	2.35189000	-2.58420900
H	5.70434000	0.59902400	-2.83743000
H	5.83683500	1.23906100	-1.19180200
C	3.31477900	1.57781000	-3.56096000
H	3.76499300	2.36987500	-4.18068200
H	2.22606700	1.72950200	-3.55458000
H	3.51701700	0.61869300	-4.06000600
C	-4.21651100	-1.09831500	-1.68830900
C	-4.55982100	0.19526400	-2.13111000
H	-5.40521800	0.25028300	-2.81470600
C	-4.11512600	1.42237100	-1.61559500
C	-5.13512100	-2.22133500	-2.07744800
H	-5.58508800	-2.67403300	-1.18163400
H	-4.57706100	-3.02387900	-2.57917400
H	-5.93560300	-1.86706600	-2.73718600
C	-4.90908800	2.64273800	-1.99182800
H	-5.23921300	3.18724900	-1.09555700
H	-5.78658800	2.37013600	-2.58935400
H	-4.28848700	3.34258800	-2.56954700
C	-3.00605700	-2.65939300	-0.36913500
C	-3.55932900	-2.93469800	0.89888400
C	-3.45124500	-4.22931500	1.41315200
H	-3.88206600	-4.45167300	2.39274100
C	-2.79423600	-5.23127800	0.71040800
H	-2.71233300	-6.23629900	1.13130600
C	-2.22891600	-4.94030300	-0.52411300
H	-1.69550000	-5.72112400	-1.07148800
C	-2.32810200	-3.66516600	-1.08908600
C	-4.26041300	-1.87185700	1.71987700
H	-4.21423800	-0.93501800	1.14658400
C	-5.73431300	-2.20742200	1.94442700
H	-6.23455000	-1.41008700	2.51628400
H	-5.85156500	-3.14466100	2.51120100
H	-6.27327600	-2.32517900	0.99219500
C	-3.53127200	-1.64102400	3.04284700
H	-2.48484400	-1.35451800	2.85706100
H	-3.53181400	-2.54964300	3.66573100
H	-4.02146200	-0.84304000	3.62183800
C	-1.69031200	-3.40479200	-2.43627700
H	-1.95643200	-2.38297600	-2.74350900
C	-2.18518800	-4.36080800	-3.52057100
H	-1.75294400	-4.08441300	-4.49478700
H	-3.28134200	-4.34302400	-3.62269300
H	-1.88988100	-5.40162500	-3.31284700
C	-0.16968500	-3.45641500	-2.32046700
H	0.19739300	-2.81561200	-1.50603300
H	0.30069500	-3.12527700	-3.25912100
H	0.17770600	-4.47826400	-2.10794700
C	-2.84493900	2.81478000	-0.17258500
C	-3.33018900	3.03135400	1.13839800
C	-3.19161300	4.30623900	1.69496300
H	-3.57163100	4.49985400	2.69888800
C	-2.58395500	5.34090400	0.99164400
H	-2.49413700	6.33144000	1.44413700
C	-2.07282000	5.10126500	-0.27536900
H	-1.56741900	5.90501400	-0.81540500
C	-2.18645300	3.84396400	-0.87697400
C	-4.00478100	1.91766500	1.92048700
H	-3.41277500	1.00563500	1.73201000
C	-5.43794200	1.65449200	1.44975200
H	-5.91667900	0.89806800	2.09031100
H	-5.48092800	1.27953300	0.41940800
H	-6.04097200	2.57471800	1.51100500
C	-4.01556600	2.16353400	3.42588600
H	-3.02004400	2.41930300	3.81433100
H	-4.36390500	1.26304800	3.95238300
H	-4.70123200	2.98041800	3.70103600
C	-1.55922100	3.61261200	-2.23475800
H	-1.94570400	2.66194900	-2.63090700

C	-1.88428900	4.70576600	-3.25007100
H	-1.41877800	5.66753000	-2.98290600
H	-2.96796600	4.87742000	-3.34508300
H	-1.49813600	4.42343800	-4.24137300
C	-0.05057100	3.44805800	-2.07879500
H	0.41029100	4.37177200	-1.70034000
H	0.41513900	3.19969300	-3.04387100
H	0.19619100	2.64191400	-1.37283400
N	1.94488500	0.28252500	3.15993500
H	1.92324800	-0.62041300	3.63635200
H	0.41346500	0.94616800	3.65648700
H	2.85674600	0.67143800	3.40102200
H	-0.79635100	0.56565700	2.41241900
N	-0.63524200	1.13670300	3.59912800
H	-0.82410100	2.13602600	3.58150200
H	-1.15104400	0.69253300	4.35579000

Cartesian coordinates of the optimized geometry of TS-**2** at PBE0-D3BJ/def2-SVP level of theory:

Ga	1.80373000	0.32974400	1.19600500
Ga	-1.84178200	-0.03151300	-0.81777000
P	-0.09135600	-0.80268900	0.46194700
P	-1.55611500	0.80228500	-3.05035100
O	1.18118800	0.50529000	-2.45841800
C	0.04532500	0.62777000	-2.69493800
N	3.27611100	-1.02652400	1.18210600
N	3.01846900	1.76041900	0.49654400
N	-2.93355300	-1.67242300	-1.07287500
N	-3.39849200	1.09162400	-0.28496000
C	4.44047400	-0.62375500	1.66263900
C	4.76801400	0.74083100	1.82270000
H	5.70233800	0.94747400	2.34400000
C	4.17227300	1.83095500	1.17313100
C	5.51011600	-1.61193000	2.03519800
H	5.90025500	-1.36349800	3.03315600
H	6.35303500	-1.53799200	1.33225100
H	5.14380700	-2.64517300	2.03391000
C	4.88525200	3.15310100	1.27455400
H	5.87718900	3.03179500	1.72556200
H	4.29420900	3.84107300	1.89918200
H	4.99039100	3.63757600	0.29521500
C	3.06442500	-2.37587400	0.77593300
C	2.26035100	-3.21977500	1.57379400
C	2.01070200	-4.51743200	1.12392100
H	1.37989400	-5.17709100	1.72172500
C	2.54023400	-4.98136900	-0.07492300
H	2.32814100	-5.99944800	-0.41063900
C	3.33625400	-4.14396300	-0.84340400
H	3.75186000	-4.51002700	-1.78570200
C	3.61189500	-2.83254400	-0.44145700
C	1.72085100	-2.76033800	2.91309200
H	1.61341400	-1.66379700	2.89454700
C	2.71271400	-3.06952200	4.03640700
H	3.68131500	-2.57719400	3.87039000
H	2.89512200	-4.15374200	4.11777000
H	2.32076600	-2.71403600	5.00237700
C	0.34715200	-3.34037000	3.22657200
H	-0.09174500	-2.81938700	4.09166100
H	0.39143400	-4.41184200	3.48205700
H	-0.33011300	-3.22523300	2.36827400
C	4.48531300	-1.96632100	-1.33034900
H	4.57403600	-0.97609700	-0.85888700
C	5.89317300	-2.54839000	-1.47324400
H	6.54421500	-1.85345400	-2.02691000
H	5.87505800	-3.49888700	-2.02992700
H	6.35815600	-2.74989900	-0.49774900
C	3.85533000	-1.75894900	-2.70622800
H	4.51349700	-1.14363500	-3.33836200
H	2.88279400	-1.25540400	-2.63492300

H	3.70301200	-2.71837300	-3.22499300
C	2.64126100	2.88891400	-0.29204000
C	1.91065300	3.94103600	0.29804500
C	1.59703400	5.05883900	-0.47986500
H	1.02983100	5.87855500	-0.03329000
C	1.99455700	5.14214600	-1.80681100
H	1.73952600	6.02116400	-2.40359900
C	2.71582600	4.09871400	-2.37663000
H	3.02098200	4.17333600	-3.42112400
C	3.05793600	2.95965400	-1.64169500
C	1.45063100	3.87569300	1.73977900
H	2.02610200	3.08273000	2.23878100
C	1.68479600	5.17565000	2.50508500
H	2.73014900	5.51330300	2.42919500
H	1.44846700	5.03653700	3.57151400
H	1.04455600	5.99273500	2.13710800
C	-0.01516500	3.45865700	1.80890700
H	-0.35060300	3.35530700	2.85285000
H	-0.19078800	2.49784700	1.30086800
H	-0.66395300	4.20338000	1.32698400
C	3.86929500	1.84705000	-2.28517400
H	3.49046200	0.90719300	-1.85953900
C	5.36330900	1.92719800	-1.95753200
H	5.78273900	2.90434900	-2.24757700
H	5.91068700	1.15041300	-2.51526200
H	5.57126600	1.76375900	-0.89235500
C	3.68913600	1.78578900	-3.79913100
H	4.18320500	2.63192300	-4.30324500
H	2.62866900	1.78410400	-4.08421900
H	4.14556300	0.86740300	-4.19550700
C	-4.11862600	-1.52862100	-1.65698200
C	-4.82136400	-0.31196100	-1.65838200
H	-5.78174400	-0.32348600	-2.17155900
C	-4.56287800	0.83544400	-0.88540000
C	-4.74953300	-2.69540800	-2.36180100
H	-4.54199600	-3.64477800	-1.85257700
H	-4.30591700	-2.76788500	-3.36867300
H	-5.83243800	-2.56314400	-2.47316700
C	-5.70212400	1.80513600	-0.73120500
H	-5.83685900	2.08790900	0.32225200
H	-6.63443100	1.37202700	-1.11104000
H	-5.49791600	2.73593600	-1.27858700
C	-2.43008200	-2.97387600	-0.76189100
C	-2.82343700	-3.56716100	0.45917900
C	-2.32007200	-4.83084300	0.77738600
H	-2.61597700	-5.30486900	1.71538700
C	-1.44074700	-5.49154700	-0.07261000
H	-1.04931500	-6.47480900	0.19921300
C	-1.05754700	-4.89154200	-1.26382200
H	-0.35672700	-5.40529400	-1.92599200
C	-1.54261900	-3.63378800	-1.63254800
C	-3.79465800	-2.87989100	1.40178600
H	-3.77978600	-1.80861900	1.15129200
C	-5.22688100	-3.37574600	1.19203100
H	-5.92024100	-2.85630600	1.87230600
H	-5.29944100	-4.45677800	1.39326500
H	-5.57632200	-3.20272500	0.16510800
C	-3.39121700	-3.02143000	2.86629900
H	-2.37278600	-2.64961400	3.04083500
H	-3.42977800	-4.06815000	3.20502300
H	-4.08030700	-2.45499200	3.50977100
C	-1.08812700	-3.02124300	-2.93893300
H	-1.58412500	-2.04468100	-3.04262500
C	-1.49009300	-3.87294800	-4.14250700
H	-1.21268000	-3.36672200	-5.08029900
H	-2.57382200	-4.06333300	-4.16557500
H	-0.98378500	-4.85144300	-4.13096700
C	0.41727300	-2.76714200	-2.92153600
H	0.71362500	-2.20279300	-2.02640600
H	0.72737000	-2.19933800	-3.81208500
H	0.98430900	-3.71148600	-2.90697400

C	-3.30017500	2.25675100	0.53995100
C	-3.37212000	2.10234700	1.94000500
C	-3.40767400	3.24702300	2.74127100
H	-3.48221200	3.13841100	3.82527900
C	-3.36369900	4.51746500	2.18166900
H	-3.40551200	5.40209800	2.82138300
C	-3.23844600	4.65481100	0.80507700
H	-3.16648800	5.65375900	0.36874300
C	-3.18807100	3.54116200	-0.03855600
C	-3.49132400	0.73309900	2.57622600
H	-3.12156600	0.00249000	1.84167500
C	-4.96021300	0.40602000	2.85104300
H	-5.06453900	-0.58066800	3.32621400
H	-5.54982100	0.39277400	1.92240800
H	-5.40775700	1.15460500	3.52411300
C	-2.63558500	0.58299200	3.83029000
H	-1.58033100	0.81649500	3.62489900
H	-2.68042800	-0.45209300	4.19870000
H	-2.97774700	1.23446800	4.64939800
C	-2.95205500	3.75523800	-1.52047000
H	-3.12333700	2.79751200	-2.03443100
C	-3.88014500	4.79674500	-2.14346300
H	-3.67817500	5.80777600	-1.75635800
H	-4.94299500	4.58010000	-1.95584300
H	-3.73023400	4.83112200	-3.23332300
C	-1.49039400	4.13409800	-1.75699400
H	-1.26052800	5.10883400	-1.29878100
H	-1.27124700	4.20025300	-2.83347400
H	-0.80150000	3.39438800	-1.32686100
N	1.57106300	0.70240000	3.02154400
H	2.38799000	1.02312500	3.53432000
H	-0.75403000	-0.66363700	1.71758200
H	0.78070100	1.28601200	3.27374600

Cartesian coordinates of the optimized geometry of TS-**3** at PBE0-D3BJ/def2-SVP level of theory:

Ga	-1.13637000	-1.34697100	-0.48289000
P	-0.51748100	-1.72649100	-2.68720100
O	0.36630900	0.23492100	-4.39351300
N	0.27548500	1.33464500	-2.11885700
H	-0.69460800	1.62930000	-2.00069100
C	0.04027100	-0.24496000	-3.36334400
C	-2.24299800	-3.96840600	0.12240000
N	-1.20243200	-3.16538200	0.34988900
P	-0.02946700	0.01157000	1.08026200
H	0.99072800	-0.98286200	1.18679400
Ga	1.15750700	1.32007000	-0.40199300
C	-3.46902600	-3.52670200	-0.38277600
H	-4.22698700	-4.29682400	-0.52219700
N	-3.15045900	-1.12923700	-0.47282900
N	1.25367400	3.21049900	0.21873800
C	-3.92953200	-2.20254000	-0.51541900
N	3.10481700	1.12803300	-0.65939000
C	-2.12759500	-5.43427300	0.43917700
H	-1.45998000	-5.91622800	-0.29042800
H	-3.10636600	-5.92571100	0.39278400
H	-1.68362000	-5.60222800	1.42966500
C	1.89282700	-4.64559500	2.80709400
H	2.68353300	-5.03650500	3.45197600
C	1.94675500	-4.84697800	1.43486200
H	2.78580800	-5.39789600	1.00309500
C	0.94133400	-4.36609300	0.58795900
C	-0.13051000	-3.65098700	1.15736000
C	-5.42581000	-2.08217000	-0.66255400
H	-5.91461700	-2.56467800	0.19632000
H	-5.74661300	-2.62956300	-1.56105100
H	-5.77731200	-1.04804300	-0.73300100
C	-0.18535300	-3.41638000	2.55153000
C	0.83278700	-3.93135800	3.35658000

H	0.79927800	-3.76498700	4.43509500
C	1.04324100	-4.62604400	-0.90024500
H	0.12317100	-4.24965900	-1.37298000
C	1.17492100	-6.11385600	-1.22782200
H	2.13236700	-6.52260400	-0.86758600
H	1.14269300	-6.26661600	-2.31760100
H	0.37236900	-6.71606800	-0.77607900
C	-1.34422900	-2.66130100	3.17387900
H	-1.76560600	-2.02521000	2.38152000
C	2.21004200	-3.84628100	-1.49294100
H	2.11314400	-2.77005500	-1.29525500
H	2.25233100	-3.98203300	-2.58392700
H	3.16765100	-4.18301300	-1.06833400
C	-2.45216300	-3.61138700	3.63243700
H	-2.07226300	-4.32608300	4.38047200
H	-2.87158700	-4.18682400	2.79566700
H	-3.27883600	-3.04692200	4.09161400
C	-0.91064200	-1.74429100	4.31213600
H	-0.56780700	-2.30919400	5.19342100
H	-1.75742300	-1.12145300	4.63837500
H	-0.10151600	-1.07263100	3.99142300
C	-3.75221200	0.16278500	-0.56137000
C	-4.16713700	0.67478200	-1.81303600
C	-4.88614000	1.87442800	-1.82896100
H	-5.23620900	2.26900000	-2.78613100
C	-5.15743100	2.57401500	-0.65989400
H	-5.72554500	3.50688300	-0.69723500
C	-4.68476500	2.09232500	0.55482500
H	-4.87922600	2.65327700	1.47112500
C	-3.98515600	0.88447200	0.62950200
C	-3.88977000	-0.03102700	-3.12774700
H	-3.27523800	-0.91886000	-2.91426900
C	-5.17137700	-0.48320300	-3.82917600
H	-5.78679700	-1.13479600	-3.19491100
H	-4.92503600	-1.03905100	-4.74700600
H	-5.79373500	0.37839300	-4.12069000
C	-3.07133300	0.85427000	-4.06633700
H	-2.15296800	1.21413800	-3.58645200
H	-3.64683300	1.73373200	-4.39841600
H	-2.76569900	0.28925900	-4.95929800
C	-3.57643700	0.32635300	1.97714200
H	-2.78721700	-0.41506300	1.79000900
C	-4.74415100	-0.40266800	2.64246300
H	-4.43486300	-0.82587600	3.61123300
H	-5.11626700	-1.22805600	2.01900300
H	-5.58540900	0.28507800	2.82635000
C	-2.98295700	1.38567200	2.89545500
H	-2.59501300	0.91879200	3.81324300
H	-3.72606600	2.13914500	3.20135700
H	-2.14759400	1.90678100	2.40950100
C	2.29338100	3.99243000	-0.05911400
C	3.50056400	3.51867200	-0.59223600
H	4.26900400	4.27093400	-0.76026100
C	3.91677600	2.18570500	-0.73563900
C	2.20994200	5.46692200	0.22200500
H	1.75504000	5.66969500	1.20051500
H	1.56608300	5.94807900	-0.52944300
H	3.20104900	5.93294600	0.17664100
C	5.40004000	1.97948900	-0.90419200
H	5.83159600	1.63454600	0.04709900
H	5.88764400	2.92194400	-1.18069000
H	5.64170300	1.21017900	-1.64499100
C	0.17934700	3.74291500	0.99420600
C	-0.88616400	4.42184500	0.37191700
C	-1.89611000	4.95645100	1.17885200
H	-2.73303500	5.47985100	0.71009300
C	-1.84794000	4.83947800	2.56162800
H	-2.64047300	5.27189400	3.17686600
C	-0.79561000	4.15496900	3.16118300
H	-0.77319400	4.05026600	4.24774600
C	0.22442800	3.58364300	2.39753200

C	-0.97089500	4.57596600	-1.13348000
H	-0.05136000	4.14557600	-1.55836500
C	-1.05895000	6.03703200	-1.57511700
H	-0.22596100	6.64227100	-1.18864000
H	-1.99260700	6.50373500	-1.22414000
H	-1.04932900	6.10788600	-2.67387600
C	-2.15789600	3.79051600	-1.68376800
H	-2.19910500	2.76647500	-1.28422400
H	-2.13135600	3.73818100	-2.78322200
H	-3.10829900	4.26317200	-1.39756500
C	1.36901500	2.84934400	3.07013900
H	1.81385600	2.19178100	2.30823200
C	2.46004400	3.81630500	3.53360800
H	3.27626300	3.26852400	4.02964000
H	2.05711600	4.54763600	4.25277900
H	2.89941200	4.37445800	2.69481700
C	0.90362200	1.95750700	4.21619100
H	1.74161900	1.34284700	4.58001300
H	0.10478900	1.28069400	3.88181800
H	0.53523800	2.54150600	5.07443900
C	3.73698500	-0.15684300	-0.68898400
C	4.03095400	-0.75031200	-1.93583400
C	4.85861700	-1.87579800	-1.93856500
H	5.12009000	-2.34390300	-2.88901600
C	5.33102400	-2.42716000	-0.75210900
H	5.97920100	-3.30642800	-0.77946900
C	4.93627100	-1.89255800	0.46781100
H	5.26303800	-2.36650700	1.39554600
C	4.13200100	-0.74946500	0.52653200
C	3.45476700	-0.20686700	-3.23416600
H	2.50508100	0.27857800	-2.96926000
C	4.32303400	0.85325400	-3.91688900
H	4.39657600	1.78324200	-3.33758200
H	3.88391100	1.11266500	-4.89230600
H	5.34375300	0.47796700	-4.09796100
C	3.12439400	-1.31576900	-4.22935000
H	4.03257800	-1.78167400	-4.64412400
H	2.54970900	-0.89494300	-5.06585100
H	2.50562800	-2.09598500	-3.76603200
C	3.71395200	-0.17456700	1.86940900
H	2.81470600	0.43399000	1.69055000
C	4.76659200	0.75211000	2.47936100
H	4.45992000	1.06149300	3.49083500
H	4.90510400	1.66806500	1.88830100
H	5.74094000	0.24454200	2.56457600
C	3.31886400	-1.26589200	2.85960200
H	4.19137700	-1.83713300	3.21438000
H	2.60973100	-1.97991600	2.41895700
H	2.84023900	-0.81936200	3.74461600
H	0.67639600	1.95896800	-2.81954300

Cartesian coordinates of the optimized geometry of TS-**4a** at PBE0-D3BJ/def2-SVP level of theory:

Ga	0.13292100	1.75691600	-0.49112200
P	-0.52042900	1.37228900	-2.68395900
O	-0.27651900	-1.00783100	-3.96846300
N	0.58548600	-1.26890600	-2.04863200
H	1.55882500	-0.99652600	-1.88879900
C	-0.08278000	-0.29153900	-2.90877200
C	-0.54718900	4.54798200	0.07266300
N	-0.92089500	3.28607200	0.27373000
P	0.04410700	0.05088400	1.14927000
H	-1.36435300	0.26412400	1.26370300
Ga	-0.17646100	-1.72738800	-0.29366900
C	0.71449400	4.92283200	-0.40222100
H	0.87152700	5.99376900	-0.52822300
N	1.86686600	2.80041300	-0.48630000
N	0.94965600	-3.28140500	0.19854300
C	1.86371700	4.12614500	-0.54467300

N	-1.85350900	-2.72922900	-0.56880900
C	-1.50720800	5.67091800	0.36080700
H	-2.12039800	5.85310700	-0.53543300
H	-0.96795600	6.59757000	0.59245400
H	-2.19438300	5.43196200	1.18183300
C	-4.43178800	2.43704800	2.45108700
H	-5.33566200	2.20799400	3.02096800
C	-4.50574500	2.65680600	1.08291900
H	-5.47338700	2.60000900	0.57859300
C	-3.36445000	2.95836700	0.33115900
C	-2.12193300	3.01241000	0.99306200
C	3.14148400	4.90050500	-0.74703100
H	3.20536300	5.71235300	-0.00947000
H	3.12200300	5.37062500	-1.74181100
H	4.03977200	4.27825300	-0.67404300
C	-2.03002400	2.79060600	2.38784600
C	-3.20055400	2.51099400	3.09596600
H	-3.14803000	2.34314400	4.17334800
C	-3.50751400	3.23391300	-1.15195900
H	-2.50745400	3.46930400	-1.54691800
C	-4.42833400	4.42509300	-1.42467500
H	-5.46616700	4.19985800	-1.13141500
H	-4.43543200	4.66417600	-2.49949600
H	-4.12276900	5.32709500	-0.87507800
C	-0.70012000	2.90329100	3.11012900
H	0.07877400	2.65038000	2.37475400
C	-4.00684800	2.00055500	-1.89331100
H	-3.37218300	1.13051900	-1.68342000
H	-3.99150300	2.16867600	-2.98052100
H	-5.03726100	1.74960300	-1.59757800
C	-0.43761100	4.33753900	3.57402100
H	-1.22072200	4.67276600	4.27325200
H	-0.41014900	5.04397100	2.73347700
H	0.53188300	4.40270700	4.09285100
C	-0.55898900	1.92957000	4.27476400
H	-1.23678500	2.17681400	5.10726300
H	0.46641500	1.96768400	4.67281400
H	-0.75657100	0.89574500	3.95766800
C	3.10587600	2.09715200	-0.54277000
C	3.75407600	1.86612800	-1.77807700
C	5.01296000	1.25580900	-1.75500200
H	5.53770500	1.08997200	-2.69925000
C	5.60036900	0.84484800	-0.56564000
H	6.58215200	0.36480300	-0.57526400
C	4.92963000	1.03729800	0.63672200
H	5.38787500	0.70405800	1.56958100
C	3.68677700	1.67401200	0.67425100
C	3.16840800	2.28722500	-3.11489200
H	2.16950300	2.71029000	-2.92734200
C	4.03076400	3.34626200	-3.80635400
H	4.22079800	4.21682300	-3.16504200
H	3.53687400	3.70007300	-4.72452600
H	5.00985300	2.93252700	-4.09708500
C	2.97733000	1.09808600	-4.05524500
H	2.32590200	0.33038300	-3.62123200
H	3.94051700	0.62982700	-4.31521100
H	2.49728000	1.42645400	-4.98918300
C	3.02689800	1.99228300	2.00141700
H	1.95140500	2.10350800	1.80299400
C	3.52996400	3.32617600	2.55553400
H	3.02738700	3.56204300	3.50703900
H	3.33777800	4.15648400	1.86224600
H	4.61513300	3.28958100	2.74438800
C	3.17934100	0.87902100	3.02764600
H	2.58047700	1.10616300	3.92254800
H	4.22316300	0.75457100	3.35791400
H	2.82963200	-0.07966900	2.62329700
C	0.57798900	-4.52232000	-0.10106200
C	-0.68256800	-4.85409600	-0.61398200
H	-0.83967400	-5.91431900	-0.81050000
C	-1.83196200	-4.05536500	-0.71215800

C	1.52113600	-5.67428500	0.11645600
H	2.31412800	-5.43892800	0.83570700
H	1.99927000	-5.92980800	-0.84194600
H	0.97171400	-6.56204100	0.45491500
C	-3.10374400	-4.81829800	-0.96825300
H	-3.10241300	-5.75456300	-0.39526300
H	-3.14988900	-5.08634000	-2.03429000
H	-4.00241200	-4.24269000	-0.72196100
C	2.18641100	-3.04209200	0.87222600
C	3.38947100	-2.95618500	0.14271800
C	4.57482800	-2.70814100	0.84248300
H	5.51367200	-2.62824700	0.28925900
C	4.57837700	-2.56969600	2.22370800
H	5.51483600	-2.38169200	2.75417600
C	3.38462600	-2.66748700	2.93102100
H	3.39400000	-2.55635300	4.01691900
C	2.17168300	-2.89325400	2.27710000
C	3.44021800	-3.14276700	-1.36128500
H	2.41275100	-3.32976000	-1.70797200
C	4.30277500	-4.34029800	-1.76421900
H	3.98292200	-5.26859200	-1.27128600
H	5.35817300	-4.17363600	-1.49698600
H	4.25900600	-4.49752600	-2.85323500
C	3.94621500	-1.88268000	-2.05729400
H	3.45577200	-0.97223500	-1.68422700
H	3.79486200	-1.94079000	-3.14589600
H	5.02116000	-1.73369200	-1.87664100
C	0.88730500	-3.04734700	3.06848400
H	0.06045700	-2.81657100	2.38058100
C	0.70587700	-4.49391800	3.53233700
H	-0.23791000	-4.60756700	4.08841700
H	1.53017700	-4.79823100	4.19728000
H	0.68166900	-5.19573600	2.68678300
C	0.77982900	-2.07694000	4.23890200
H	-0.20901200	-2.16941300	4.71359300
H	0.89693500	-1.03888200	3.89704800
H	1.53021600	-2.28034200	5.01908100
C	-3.11603800	-2.05097800	-0.48367100
C	-3.86703600	-1.75479600	-1.64220900
C	-5.11819900	-1.15147300	-1.47288900
H	-5.71623900	-0.92322500	-2.35801700
C	-5.60368800	-0.81935200	-0.21703800
H	-6.57802900	-0.33546800	-0.11507000
C	-4.84160800	-1.09586100	0.91167700
H	-5.22368000	-0.82603600	1.89690400
C	-3.60104300	-1.72777800	0.80567700
C	-3.41362100	-2.08199500	-3.05501700
H	-2.40060600	-2.50891200	-2.99891000
C	-4.34127100	-3.09927500	-3.72800500
H	-4.49405300	-4.00785000	-3.13124000
H	-3.93212200	-3.39523900	-4.70629200
H	-5.33438500	-2.65845500	-3.90948600
C	-3.33278100	-0.83320800	-3.93282400
H	-4.33249000	-0.39610000	-4.08757400
H	-2.91380600	-1.09125400	-4.91552300
H	-2.68393700	-0.06352800	-3.49672600
C	-2.84489500	-2.13060600	2.05894400
H	-1.77039700	-2.08226600	1.82383000
C	-3.15521000	-3.57560500	2.45470300
H	-2.60606700	-3.84943500	3.36939600
H	-2.86972400	-4.29136300	1.67208000
H	-4.23137000	-3.70175300	2.65366500
C	-3.07846800	-1.19313200	3.23720400
H	-4.09563800	-1.29415200	3.64790700
H	-2.92966500	-0.14148700	2.95587800
H	-2.37689500	-1.43081100	4.05062600
H	0.33172300	-1.81583700	-3.19345800

Cartesian coordinates of the optimized geometry of TS-**4b** at PBE0-D3BJ/def2-SVP level of theory:

Ga	-0.99022000	1.47755100	-0.58604200
P	-1.17777600	0.59370800	-2.71034400
O	0.24559400	-1.42396700	-3.70297300
N	1.27747800	-0.58548800	-1.84833900
H	1.48624600	0.37609400	-1.57822400
C	0.17601700	-0.55692800	-2.78546900
C	-3.19979100	3.36784100	-0.20423500
N	-2.76966700	2.14333700	0.08961700
P	-0.09347100	0.21032700	1.22815600
H	-1.34034000	-0.47476300	1.35736800
Ga	0.84640700	-1.39614400	-0.11591000
C	-2.38263100	4.38433100	-0.71346800
H	-2.88298800	5.32888000	-0.92655000
N	-0.20291200	3.35275300	-0.62629400
N	2.68567800	-1.97322400	0.44825100
C	-0.97985100	4.41224200	-0.80143000
N	0.10735200	-3.22600100	-0.36191400
C	-4.64641600	3.73048100	0.00506200
H	-5.19864300	3.53499800	-0.92735800
H	-4.75230300	4.79759300	0.23743400
H	-5.11679000	3.13526600	0.79716200
C	-5.13641800	-0.53167100	2.33567700
H	-5.73370000	-1.23398900	2.92257900
C	-5.29687600	-0.45788600	0.95949300
H	-6.02572800	-1.10503500	0.46558700
C	-4.54657300	0.43360300	0.18417900
C	-3.59952600	1.25216400	0.83193300
C	-0.39206400	5.76984500	-1.09261000
H	-0.85252500	6.52572800	-0.44160100
H	-0.63144700	6.04920200	-2.12985100
H	0.69569600	5.80221200	-0.96603800
C	-3.43429700	1.19759300	2.23721700
C	-4.21517600	0.29879400	2.96698700
H	-4.10072600	0.24705000	4.05135200
C	-4.80100700	0.50496000	-1.30801900
H	-4.11140400	1.25141500	-1.73078400
C	-6.23708200	0.93569600	-1.61551900
H	-6.95818000	0.16721400	-1.29357300
H	-6.36970700	1.07711600	-2.69956800
H	-6.51265200	1.87297100	-1.11145700
C	-2.47148700	2.13262200	2.94548200
H	-1.66261100	2.34913300	2.23120700
C	-4.49171200	-0.82292500	-1.98624800
H	-3.46756000	-1.14428200	-1.76233700
H	-4.57494600	-0.72630100	-3.07913200
H	-5.18569200	-1.61205600	-1.65588200
C	-3.14193700	3.46448100	3.28905000
H	-4.00076600	3.30871700	3.96196100
H	-3.50462600	3.98590100	2.39337200
H	-2.42934200	4.13423800	3.79621100
C	-1.83250400	1.52278200	4.18789500
H	-2.56332500	1.37057600	4.99814600
H	-1.05568300	2.19731400	4.57868600
H	-1.35480500	0.55908000	3.96105200
C	1.21321700	3.49549200	-0.62419700
C	1.94291500	3.56017600	-1.83423400
C	3.32638200	3.75808500	-1.75744100
H	3.90148500	3.83153200	-2.68396500
C	3.98182900	3.85533300	-0.53762600
H	5.06397700	4.00555800	-0.50540900
C	3.25583400	3.75559300	0.64385300
H	3.77370700	3.82404900	1.60195900
C	1.87000800	3.58619100	0.62555300
C	1.29126600	3.48080900	-3.20450000
H	0.22409100	3.25265700	-3.05774900
C	1.42121000	4.80091000	-3.96878700
H	1.03939600	5.65659800	-3.39703600
H	0.86434200	4.74904200	-4.91730400
H	2.47427600	5.01420300	-4.21459600

C	1.86846000	2.34803000	-4.05179100
H	1.75864700	1.37591800	-3.55647900
H	2.93584100	2.51129600	-4.27451500
H	1.32852600	2.28048500	-5.00811600
C	1.07753100	3.59044400	1.91791100
H	0.15430100	3.02639600	1.72222900
C	0.67311400	5.01218400	2.31079700
H	0.07994400	5.00080800	3.23906100
H	0.06567100	5.49552700	1.53398300
H	1.56227500	5.64024600	2.48325200
C	1.79654200	2.89304800	3.06374200
H	1.12657000	2.80607100	3.93233300
H	2.68860100	3.44812400	3.39650400
H	2.10746300	1.88107800	2.77377600
C	3.13020800	-3.18705100	0.16180400
C	2.33291900	-4.19757200	-0.40793300
H	2.84344200	-5.14887100	-0.56167800
C	0.92802100	-4.26509200	-0.49161200
C	4.55149700	-3.58128300	0.46573000
H	5.05663200	-2.85609400	1.11355200
H	5.12137500	-3.65950000	-0.47282200
H	4.57423100	-4.57047800	0.94255900
C	0.38201800	-5.65084900	-0.70504000
H	0.97750900	-6.38250700	-0.14416300
H	0.45675300	-5.90948500	-1.77163200
H	-0.66992700	-5.73495300	-0.41122800
C	3.52320300	-1.01259400	1.09635300
C	4.50389700	-0.30457900	0.36746000
C	5.25263500	0.67079900	1.03334000
H	6.00266000	1.23695300	0.47605800
C	5.06774100	0.92456600	2.38469900
H	5.66340400	1.69025100	2.88713800
C	4.12631300	0.19239300	3.09896500
H	3.99642900	0.38274300	4.16553200
C	3.33807400	-0.77813400	2.47760000
C	4.81618700	-0.59383200	-1.08860200
H	4.16295400	-1.41751100	-1.40617800
C	6.26767900	-1.04298900	-1.27611000
H	6.53363000	-1.88410700	-0.62108000
H	6.96676900	-0.22152500	-1.05521400
H	6.44632900	-1.34978800	-2.31921600
C	4.52260700	0.60170200	-1.98868000
H	3.50584800	0.98438900	-1.84762900
H	4.64240300	0.33394500	-3.05081200
H	5.20976400	1.43442000	-1.77783300
C	2.38327400	-1.62597000	3.29418100
H	1.59729200	-1.97253200	2.60773700
C	3.10332800	-2.86412500	3.83293600
H	2.41045700	-3.49806500	4.40804500
H	3.93151400	-2.57513600	4.49980800
H	3.52484100	-3.47727300	3.02373500
C	1.69325600	-0.86055300	4.41614200
H	0.96015600	-1.51018700	4.91741800
H	1.15733300	0.01340500	4.01959300
H	2.40201400	-0.52442900	5.18919700
C	-1.31091500	-3.44563200	-0.24561900
C	-2.11443400	-3.73771200	-1.37073400
C	-3.47983900	-3.96266400	-1.15974200
H	-4.11164800	-4.19010500	-2.02089100
C	-4.04938300	-3.88398000	0.10060700
H	-5.12188600	-4.04552600	0.23183100
C	-3.24729900	-3.59587000	1.19717300
H	-3.69662900	-3.53639700	2.18864000
C	-1.87400700	-3.39255200	1.05296100
C	-1.59671000	-3.86737800	-2.79308600
H	-0.52487300	-3.62086900	-2.79192600
C	-1.78963800	-5.28931000	-3.33401300
H	-1.39707000	-6.06901200	-2.66792100
H	-1.29662600	-5.39232400	-4.31340400
H	-2.85916000	-5.50384600	-3.48678200
C	-2.28020600	-2.88497600	-3.74381900

H	-3.35655100	-3.10662500	-3.82821100
H	-1.83499400	-2.96436400	-4.74579800
H	-2.15637500	-1.84408400	-3.41939300
C	-1.01060700	-3.21376300	2.28775600
H	-0.18976000	-2.53110300	2.01997300
C	-0.37882600	-4.54006200	2.71506700
H	0.23762700	-4.40046600	3.61693700
H	0.26910900	-4.96216600	1.93462200
H	-1.15758300	-5.28378800	2.94737500
C	-1.74723400	-2.57216200	3.45747300
H	-2.48631600	-3.25687100	3.90275600
H	-2.27202300	-1.65577300	3.15261200
H	-1.03349500	-2.30780200	4.25111600
H	2.29405500	-1.43112500	-2.51538300
H	1.54206900	-2.11149000	-3.70084800
N	2.54940400	-2.27414400	-3.26219200
H	2.64064500	-3.16610800	-2.77241900
H	3.32751300	-2.10929900	-3.89536200

Cartesian coordinates of the optimized geometry of TS-**5** at PBE0-D3BJ/def2-SVP level of theory:

Ga	1.14008700	1.32617200	-0.49477000
P	0.74567600	1.16690100	-2.77328800
O	-0.22131500	-0.67773000	-4.35277900
N	-0.74238800	-1.21523500	-2.22490000
H	-1.05981600	-2.00715800	-2.78156300
C	-0.12947500	-0.32051300	-3.01938100
C	1.96391000	4.10573600	-0.02794300
N	0.98475700	3.21950400	0.16136100
P	-0.04087400	0.04690000	1.10573800
H	-1.07775700	1.03352300	1.08180400
Ga	-1.17233200	-1.31121500	-0.36048300
C	3.27273200	3.75587300	-0.36261300
H	3.97038800	4.58382600	-0.48305000
N	3.16982400	1.33932800	-0.30874500
N	-1.00464200	-3.22740100	0.08695900
C	3.85391200	2.47317600	-0.35901900
N	-3.17037500	-1.36457700	-0.39597600
C	1.67639500	5.57366100	0.13763300
H	1.08279000	5.92940200	-0.71797200
H	2.60558700	6.15407600	0.17859400
H	1.08199500	5.77594400	1.03852300
C	-2.51635000	4.52290100	2.11156400
H	-3.41906600	4.86325300	2.62501000
C	-2.42035200	4.62010500	0.72978300
H	-3.25401600	5.03583900	0.15867800
C	-1.27052900	4.20252300	0.05152600
C	-0.21057100	3.65659700	0.80103100
C	5.36069000	2.47496500	-0.38732900
H	5.74282400	3.21163200	0.33259500
H	5.70353100	2.78850200	-1.38468800
H	5.79491100	1.49443900	-0.16466300
C	-0.29743300	3.54013000	2.20806500
C	-1.45830900	3.98928600	2.84157600
H	-1.53756500	3.91757100	3.92839000
C	-1.19396200	4.34335200	-1.45452900
H	-0.17865500	4.05368500	-1.76525600
C	-1.45112200	5.77578800	-1.92247500
H	-2.48843100	6.08574000	-1.71835200
H	-1.29261900	5.85658100	-3.00904500
H	-0.78987600	6.50236900	-1.42698500
C	0.86094700	2.98920900	3.01975000
H	1.45426800	2.36360100	2.33610100
C	-2.15833100	3.37950600	-2.13257700
H	-1.99757200	2.35221100	-1.77926000
H	-2.00734100	3.38525800	-3.22237200
H	-3.20399100	3.64978800	-1.92076300
C	1.77594900	4.11116100	3.51516300
H	1.22323500	4.81338100	4.16029200

H	2.20978000	4.68403800	2.68418300
H	2.61067600	3.69629100	4.10175200
C	0.41794600	2.10359300	4.17951700
H	-0.12990700	2.66897800	4.95009000
H	1.29842800	1.66112000	4.67009400
H	-0.21993200	1.27965300	3.82925400
C	3.86960400	0.09611000	-0.26449200
C	4.48123500	-0.43888700	-1.42132200
C	5.17601800	-1.64754100	-1.29560400
H	5.66501000	-2.06879500	-2.17760200
C	5.25285300	-2.32222100	-0.08514800
H	5.79571700	-3.26832400	-0.01682800
C	4.63993300	-1.78585800	1.04183600
H	4.70602400	-2.31435800	1.99387300
C	3.95615000	-0.57048900	0.97936400
C	4.47597300	0.25221900	-2.77527400
H	3.91447800	1.19374600	-2.67484700
C	5.89934300	0.57368400	-3.24329100
H	6.48826400	1.10265100	-2.48181200
H	5.87038200	1.19798100	-4.14989400
H	6.44805100	-0.34678700	-3.49915800
C	3.76525900	-0.57199300	-3.84738400
H	2.70409700	-0.70598700	-3.60598700
H	4.23575300	-1.56051500	-3.97252500
H	3.81359000	-0.04994000	-4.81547200
C	3.40965000	0.07135700	2.23957300
H	2.54021900	0.67419300	1.94072500
C	4.43544700	1.02402300	2.85600500
H	4.02673400	1.49331500	3.76510100
H	4.71598900	1.82979600	2.16404700
H	5.35406400	0.48367700	3.13635100
C	2.91573500	-0.93564600	3.26703700
H	2.39999900	-0.41459300	4.08730100
H	3.74060800	-1.51248100	3.71588500
H	2.20682400	-1.64292700	2.81768300
C	-1.95049400	-4.12204400	-0.19531400
C	-3.24975500	-3.77465100	-0.58048700
H	-3.92750000	-4.60447700	-0.77659600
C	-3.84261400	-2.49976200	-0.55424600
C	-1.63408400	-5.58700700	-0.07862600
H	-1.16830800	-5.82085000	0.88835100
H	-0.90409100	-5.87431000	-0.85052300
H	-2.53652100	-6.19669900	-0.20305500
C	-5.34108900	-2.48931300	-0.70500300
H	-5.77978300	-3.31922900	-0.13538400
H	-5.60423400	-2.64457800	-1.76226400
H	-5.79195600	-1.54564900	-0.37751000
C	0.17829200	-3.66769800	0.75301900
C	1.27699200	-4.13511000	0.00771500
C	2.40694800	-4.57521800	0.70232500
H	3.27524800	-4.92814800	0.14193300
C	2.43962700	-4.57482200	2.09151000
H	3.32812300	-4.93197300	2.61784000
C	1.34416400	-4.11119200	2.81256000
H	1.38112100	-4.10812300	3.90419800
C	0.20118400	-3.63648900	2.16418700
C	1.24772900	-4.15790500	-1.50656300
H	0.20434100	-3.99080300	-1.81352700
C	1.69364900	-5.49957200	-2.08511000
H	1.12224800	-6.34276000	-1.66662500
H	2.75939500	-5.69170900	-1.88560400
H	1.56276100	-5.50600000	-3.17820900
C	2.07624100	-3.01597500	-2.08382800
H	1.78480700	-2.04497100	-1.66054300
H	1.94842600	-2.95389500	-3.17493700
H	3.14438900	-3.15765100	-1.86805500
C	-0.99753000	-3.15753700	2.96407400
H	-1.62732800	-2.57402200	2.27596200
C	-1.83871000	-4.33461700	3.46188700
H	-2.71078500	-3.97351900	4.02959200
H	-1.24937900	-4.98721400	4.12616200

H	-2.21531200	-4.95062100	2.63291700
C	-0.61571400	-2.23345200	4.11601000
H	-1.52369900	-1.86623300	4.61948700
H	-0.05476900	-1.36324400	3.74729600
H	-0.00887100	-2.74744500	4.87814800
C	-3.86977500	-0.12215300	-0.34949900
C	-4.36978500	0.46110200	-1.53348300
C	-5.05697400	1.67443800	-1.42913400
H	-5.45861100	2.13836500	-2.33335800
C	-5.22563200	2.30571000	-0.20455000
H	-5.75473500	3.25992300	-0.14790600
C	-4.71353100	1.72485200	0.95069400
H	-4.84336400	2.23133300	1.90768900
C	-4.04112800	0.50195900	0.90674100
C	-4.21600100	-0.18553400	-2.89993300
H	-3.72642600	-1.15973100	-2.75439600
C	-5.56922500	-0.41973800	-3.57557900
H	-6.27277700	-0.95857000	-2.92592900
H	-5.44289000	-1.00124200	-4.50227700
H	-6.04454000	0.53437900	-3.85207600
C	-3.31426300	0.63423400	-3.82192800
H	-3.75981200	1.61557700	-4.04681500
H	-3.18791900	0.11200400	-4.78621000
H	-2.32985100	0.82379000	-3.37197400
C	-3.56819000	-0.17456400	2.18043900
H	-2.62565800	-0.69215200	1.94064600
C	-4.56093500	-1.24064800	2.64823900
H	-4.20196300	-1.71612700	3.57465500
H	-4.69818600	-2.03546400	1.90264600
H	-5.54643100	-0.79395100	2.85597000
C	-3.26001500	0.80171600	3.30754900
H	-4.17432500	1.26139100	3.71622400
H	-2.59345200	1.60804100	2.97230700
H	-2.76427900	0.27363200	4.13552500
H	-1.08870300	-0.39633300	-4.67283400

Cartesian coordinates of the optimized geometry of TS-**6a** at PBE0-D3BJ/def2-SVP level of theory:

Ga	1.30584600	1.27471800	-0.51628300
P	0.53765000	1.21494300	-2.69547000
H	1.00759100	0.36788000	-4.07841600
O	0.33207200	-0.77349200	-4.17442800
N	-0.85985500	-1.30350500	-2.30730200
H	-1.03695800	-2.16054400	-2.83037100
C	-0.08289600	-0.47701200	-2.99418300
C	2.33143000	3.95618500	0.02367500
N	1.27437600	3.15785700	0.16894400
P	-0.05954500	0.04792900	0.97289200
H	-1.00755300	1.11904100	0.96064800
Ga	-1.32802600	-1.25719700	-0.44319900
C	3.61267100	3.49693600	-0.29705700
H	4.38238400	4.26320400	-0.38214200
N	3.29840600	1.09767000	-0.30050600
N	-1.30907100	-3.16025200	0.06982200
C	4.08074400	2.17119800	-0.31208000
N	-3.30641400	-1.11753000	-0.44765400
C	2.17908300	5.43984200	0.22057300
H	1.73680500	5.87791500	-0.68735400
H	3.15155800	5.91667200	0.39118000
H	1.50365300	5.67946500	1.05175200
C	-2.10186900	4.67954700	2.19175700
H	-2.96863600	5.07662900	2.72583800
C	-2.01502800	4.80178700	0.81217200
H	-2.82033000	5.29520600	0.26283300
C	-0.91105600	4.31275900	0.10449900
C	0.11369600	3.66873000	0.82521200
C	5.58130400	2.03617400	-0.31712400
H	6.01982400	2.74394800	0.39920900
H	5.96579800	2.30182800	-1.31330400

H	5.92033900	1.02259200	-0.07707700
C	0.03262500	3.52340300	2.23051200
C	-1.08021200	4.04597600	2.89228300
H	-1.15029800	3.95259400	3.97804400
C	-0.85497100	4.50401900	-1.39695400
H	0.08776600	4.06173400	-1.75360900
C	-0.88081300	5.98170200	-1.79183500
H	-1.84314800	6.44803700	-1.52678100
H	-0.75263800	6.08846600	-2.88024700
H	-0.09014100	6.56397400	-1.29671900
C	1.15110600	2.86798000	3.01960600
H	1.71053400	2.23665800	2.31345100
C	-1.99249200	3.75550300	-2.08072700
H	-2.01225600	2.70273600	-1.77267900
H	-1.86814000	3.77757400	-3.17370800
H	-2.96787600	4.20247600	-1.83333500
C	2.12716900	3.91229800	3.56558900
H	1.61395700	4.61070300	4.24638500
H	2.58801400	4.50296100	2.76185100
H	2.93979300	3.42559700	4.12738800
C	0.64501600	1.96332700	4.13829500
H	0.12275800	2.52840100	4.92635600
H	1.49199300	1.45008800	4.61849900
H	-0.03777100	1.19490600	3.74833500
C	3.88567000	-0.20405800	-0.26319500
C	4.47278400	-0.76606800	-1.41859800
C	5.06504600	-2.02886800	-1.30777600
H	5.53336700	-2.47269700	-2.18977900
C	5.06129500	-2.73040400	-0.11003000
H	5.52305500	-3.71917800	-0.05101700
C	4.46751400	-2.16935800	1.01498200
H	4.46640200	-2.72243800	1.95514900
C	3.88721600	-0.90014900	0.96674000
C	4.52308800	-0.05619200	-2.76008800
H	4.04839000	0.92947600	-2.64035700
C	5.96250300	0.14687700	-3.24187800
H	6.59923200	0.62605400	-2.48570300
H	5.97701400	0.77138800	-4.14863800
H	6.43149900	-0.81531000	-3.50204400
C	3.72823200	-0.80880800	-3.82426100
H	2.67728400	-0.92489700	-3.53950700
H	4.15002500	-1.81119000	-4.00236400
H	3.75452600	-0.26088600	-4.77904200
C	3.36213700	-0.24548600	2.22900600
H	2.54506700	0.42518200	1.92658500
C	4.44260700	0.61539300	2.88581400
H	4.05273300	1.09161200	3.79936300
H	4.79369400	1.41496600	2.21895200
H	5.31410200	0.00293700	3.16802700
C	2.77245600	-1.23485400	3.22246000
H	2.28376000	-0.69542800	4.04693800
H	3.54114300	-1.88523000	3.67020600
H	2.01855400	-1.87354600	2.74454900
C	-2.33316500	-3.97021400	-0.20305100
C	-3.59392700	-3.51382200	-0.60113700
H	-4.33963700	-4.28352000	-0.79468500
C	-4.07483700	-2.19064200	-0.59620400
C	-2.14856100	-5.45478600	-0.05880200
H	-1.74793300	-5.71314900	0.93105900
H	-1.41105700	-5.81378400	-0.79248300
H	-3.09333200	-5.98760800	-0.21665500
C	-5.56235000	-2.04108500	-0.76372100
H	-6.08442000	-2.88389800	-0.29321800
H	-5.81192200	-2.05262800	-1.83558600
H	-5.93591200	-1.09834100	-0.34663800
C	-0.17871700	-3.69359000	0.76021000
C	0.89549500	-4.25127300	0.04071100
C	1.96804000	-4.78648500	0.75958800
H	2.81558000	-5.21103200	0.21708100
C	1.96811700	-4.79361600	2.14901300
H	2.80956300	-5.22759200	2.69458100

C	0.89992900	-4.23768800	2.84500900
H	0.91128300	-4.23731700	3.93741300
C	-0.18190000	-3.66476900	2.17168400
C	0.90627500	-4.26833900	-1.47350500
H	-0.10704100	-3.99672400	-1.80496000
C	1.22459400	-5.64742900	-2.04829500
H	0.54946700	-6.42493900	-1.65835000
H	2.25496900	-5.95593100	-1.81238600
H	1.13302400	-5.63369200	-3.14527100
C	1.86708700	-3.21762800	-2.02101600
H	1.68749800	-2.23029300	-1.57270100
H	1.75778300	-3.11373000	-3.11064600
H	2.90938200	-3.48651600	-1.79900000
C	-1.34190700	-3.07229500	2.95138500
H	-1.93803800	-2.48204800	2.23972600
C	-2.25294500	-4.16554100	3.51245800
H	-3.09873300	-3.72076600	4.05999700
H	-1.70403200	-4.81751100	4.21103700
H	-2.66976100	-4.80058800	2.71753100
C	-0.88346900	-2.12102400	4.05282900
H	-1.75672100	-1.67615000	4.55480200
H	-0.27676500	-1.30577500	3.63367500
H	-0.29221500	-2.63621200	4.82626800
C	-3.89294200	0.18370900	-0.40281600
C	-4.33065400	0.80997600	-1.58891600
C	-4.91331900	2.07691000	-1.48288800
H	-5.26159000	2.57839200	-2.38896100
C	-5.03798100	2.71510200	-0.25668400
H	-5.48625300	3.70995600	-0.19991100
C	-4.57995600	2.09239200	0.89948700
H	-4.66996100	2.60659800	1.85702700
C	-4.01047300	0.81823700	0.85412400
C	-4.19504200	0.16941800	-2.95931400
H	-3.79776600	-0.84645900	-2.81654700
C	-5.54155400	0.06409200	-3.67887100
H	-6.31108300	-0.42626700	-3.06509100
H	-5.43213700	-0.50640600	-4.61419700
H	-5.92599300	1.06003200	-3.94943800
C	-3.19249600	0.92029100	-3.83478000
H	-3.56121200	1.92895300	-4.08041100
H	-3.02923100	0.38145000	-4.78093800
H	-2.21816300	1.03531600	-3.34158800
C	-3.58746300	0.10677900	2.12652500
H	-2.69504600	-0.49126200	1.88074100
C	-4.66410700	-0.86856300	2.60647200
H	-4.34123600	-1.37008900	3.53247800
H	-4.87550200	-1.65110500	1.86498000
H	-5.60571200	-0.33751900	2.81910400
C	-3.18607700	1.05681800	3.24685600
H	-4.05257300	1.60079300	3.65597400
H	-2.44999400	1.79712300	2.90473100
H	-2.73759700	0.49081600	4.07657000

Cartesian coordinates of the optimized geometry of TS-**6b** at PBE0-D3BJ/def2-SVP level of theory:

Ga	1.07643800	-1.35918500	0.34048900
P	0.34855900	-1.32288500	2.51116000
H	0.90858500	-0.38063800	5.10518600
O	0.17659100	0.73956100	4.22759400
N	-0.60821100	1.32500500	2.21625400
H	-0.77894300	2.17486400	2.75475500
C	-0.02697800	0.41601100	3.02952300
C	1.82263400	-4.14460600	-0.20579500
N	0.85539700	-3.24212300	-0.34676800
P	-0.06589600	0.04406900	-1.16814300
H	-1.13365700	-0.90869900	-1.18165300
Ga	-1.12816700	1.37738000	0.38656600
C	3.14491000	-3.82606100	0.12538000
H	3.82696600	-4.67194900	0.20780300

N	3.09243700	-1.40878800	0.11858800
N	-0.93369400	3.29800400	-0.07771400
C	3.75343000	-2.55980100	0.14342800
N	-3.12395500	1.46410500	0.43968800
C	1.52863800	-5.60639500	-0.41595200
H	1.17510900	-6.03782400	0.53347400
H	2.43362300	-6.14922300	-0.71515900
H	0.74068700	-5.77079600	-1.16113600
C	-2.70264800	-4.35453400	-2.32396100
H	-3.61968600	-4.63986800	-2.84536000
C	-2.59914400	-4.52113700	-0.95020000
H	-3.44098800	-4.93895300	-0.39292300
C	-1.43360000	-4.17277700	-0.25843000
C	-0.36140100	-3.62205300	-0.98829800
C	5.26091600	-2.58961300	0.16439200
H	5.62781900	-3.34101600	-0.54781600
H	5.60559600	-2.89481300	1.16391800
H	5.71048800	-1.61943700	-0.07394200
C	-0.45433400	-3.44087500	-2.38865200
C	-1.63167100	-3.82249600	-3.03500700
H	-1.71289700	-3.69880500	-4.11690900
C	-1.36053100	-4.42756900	1.23327300
H	-0.37527600	-4.08178300	1.58186600
C	-1.50621700	-5.91440500	1.56466000
H	-2.51419500	-6.28024100	1.31129700
H	-1.35772300	-6.08298700	2.64296600
H	-0.78621900	-6.54039900	1.01854100
C	0.71980600	-2.90946600	-3.18949800
H	1.35233400	-2.34463100	-2.48871700
C	-2.41129700	-3.61599500	1.97969900
H	-2.33020200	-2.55139000	1.73014800
H	-2.27195500	-3.71161100	3.06740100
H	-3.42892900	-3.95572500	1.73152900
C	1.56749500	-4.05707200	-3.74272100
H	0.97405800	-4.69001900	-4.42238100
H	1.95996600	-4.69940800	-2.94234600
H	2.42817100	-3.66538100	-4.30729000
C	0.30848700	-1.95484800	-4.30513600
H	-0.28209700	-2.45799800	-5.08703400
H	1.20389100	-1.54233100	-4.79454500
H	-0.27723200	-1.11217600	-3.91080300
C	3.81514500	-0.18007500	0.07120100
C	4.44510300	0.33589900	1.22527500
C	5.16192100	1.53190500	1.10871000
H	5.66493300	1.93681800	1.99056900
C	5.24222500	2.21565300	-0.09567800
H	5.79972900	3.15330700	-0.15997100
C	4.61114300	1.69984900	-1.22194800
H	4.67750800	2.23806000	-2.16813000
C	3.90518500	0.49681500	-1.16735400
C	4.43426900	-0.36981200	2.57085400
H	3.86394800	-1.30469200	2.45828900
C	5.85479800	-0.70652200	3.03593900
H	6.43844200	-1.22699300	2.26490600
H	5.82585000	-1.34610000	3.93251700
H	6.40945500	0.20636300	3.30513800
C	3.73143000	0.45934000	3.64331900
H	2.65874900	0.56810500	3.44090800
H	4.17103000	1.46517000	3.72587500
H	3.85327800	-0.02049500	4.62859000
C	3.33033700	-0.11504100	-2.42965100
H	2.42334300	-0.66174900	-2.13394300
C	4.30081400	-1.12705500	-3.04119600
H	3.87530600	-1.55813300	-3.96126500
H	4.51619400	-1.95832400	-2.35627900
H	5.25681600	-0.64558500	-3.30333600
C	2.90503900	0.91881400	-3.46165300
H	2.35761100	0.42977200	-4.28065900
H	3.76750700	1.43675200	-3.91181900
H	2.24361600	1.67314700	-3.01619500
C	-1.86002000	4.20436900	0.23014100

C	-3.15419400	3.87437200	0.64531900
H	-3.81224500	4.71345600	0.86714000
C	-3.76927300	2.60918900	0.62881500
C	-1.52678000	5.66666600	0.11880100
H	-1.02779500	5.89594100	-0.83234500
H	-0.82191300	5.94844700	0.91585300
H	-2.42714700	6.28463600	0.21494800
C	-5.26127000	2.62181800	0.83193300
H	-5.70221700	3.49459700	0.33299900
H	-5.48093900	2.71121200	1.90658300
H	-5.74408500	1.70863900	0.46535200
C	0.24590400	3.72102100	-0.75849700
C	1.36407400	4.17347800	-0.03176500
C	2.49274800	4.58964900	-0.74344200
H	3.37352500	4.93174000	-0.19539500
C	2.50886100	4.58046000	-2.13256000
H	3.39677200	4.91863000	-2.67241800
C	1.39450700	4.13505500	-2.83628000
H	1.41544500	4.12919400	-3.92824800
C	0.25113700	3.68642000	-2.17055500
C	1.35966800	4.21273100	1.48262200
H	0.32514500	4.02913600	1.80776600
C	1.78940600	5.57004700	2.03739000
H	1.19355500	6.39681100	1.62093900
H	2.84672500	5.78129100	1.81298100
H	1.67977000	5.58689700	3.13283700
C	2.21995300	3.09560600	2.06245200
H	1.95610400	2.12038600	1.63182900
H	2.08371400	3.02498900	3.15209900
H	3.28473800	3.27168000	1.85168800
C	-0.97118100	3.23704500	-2.95100600
H	-1.58388900	2.63741400	-2.26157600
C	-1.81869900	4.43314100	-3.38945000
H	-2.70613700	4.09381000	-3.94665500
H	-1.24245100	5.10461000	-4.04651400
H	-2.17249000	5.02273000	-2.53198000
C	-0.62965700	2.34564800	-4.14005700
H	-1.55477000	1.99285500	-4.62198000
H	-0.05863500	1.46518900	-3.81365300
H	-0.04883300	2.87978800	-4.90874500
C	-3.85473900	0.23948900	0.38286000
C	-4.33199200	-0.36477800	1.56550600
C	-5.06221200	-1.55134800	1.44570800
H	-5.44284500	-2.03321600	2.34951500
C	-5.29506200	-2.13607500	0.20852500
H	-5.85799400	-3.07020100	0.14092000
C	-4.80165600	-1.53665800	-0.94538400
H	-4.98022500	-2.00719200	-1.91304200
C	-4.08593700	-0.33927400	-0.88522900
C	-4.09773900	0.22474500	2.94568600
H	-3.57094100	1.18139800	2.81469400
C	-5.41169000	0.49091400	3.68404100
H	-6.11571500	1.09241300	3.09154300
H	-5.21845900	1.02028400	4.63013000
H	-5.92130200	-0.45226200	3.93717200
C	-3.19453000	-0.66622800	3.79730100
H	-3.69965900	-1.61292500	4.04918900
H	-2.93427600	-0.15706200	4.73819400
H	-2.25722100	-0.91432700	3.28022800
C	-3.62969000	0.36118400	-2.15204400
H	-2.67599500	0.85994900	-1.91794600
C	-4.61602300	1.45220500	-2.57353300
H	-4.27083300	1.94429100	-3.49667100
H	-4.72508900	2.23097100	-1.80659800
H	-5.61250300	1.02457200	-2.76905100
C	-3.35896300	-0.59174400	-3.30853100
H	-4.28776200	-1.03085300	-3.70709200
H	-2.69609800	-1.41419900	-3.00705400
H	-2.87496100	-0.05082400	-4.13535200
H	1.21254300	-1.61610700	4.03877200
N	1.37512800	-1.33398700	5.24261200

H	0.82714500	-1.93937900	5.85032100
H	2.33571300	-1.25675000	5.56950600

Cartesian coordinates of the optimized geometry of Int-**7** at PBE0-D3BJ/def2-SVP level of theory:

Ga	-1.55705600	-0.13165300	0.56574800
Ga	1.60401300	0.15084000	-0.53548300
P	0.48661300	0.06656800	1.41726300
P	0.67476900	0.08952200	-2.74849500
O	-1.77395900	-0.18341300	-1.38775600
C	-0.97118200	-0.09630300	-2.37801900
N	-3.04386100	1.19475700	0.95937900
N	-2.76299600	-1.70390000	0.99742700
N	2.85898500	1.72337500	-0.78304400
N	3.11986700	-1.16646400	-0.84063500
C	-4.05756300	0.90853700	1.77599900
C	-4.37171600	-0.38167300	2.22103900
H	-5.21147000	-0.45376400	2.90957900
C	-3.81227900	-1.59848600	1.81200200
C	-4.95184300	2.00946800	2.28219000
H	-5.86341400	1.59332000	2.72702300
H	-5.22434200	2.72062800	1.49195600
H	-4.42507800	2.58869200	3.05353200
C	-4.47382100	-2.83860600	2.35257900
H	-5.43744100	-2.59367100	2.81449700
H	-3.83071800	-3.29822700	3.11589700
H	-4.62561200	-3.60043800	1.57721400
C	-2.95379500	2.54727800	0.50629500
C	-2.31631000	3.53246200	1.28840000
C	-2.35574300	4.85873000	0.84142300
H	-1.86070900	5.63090500	1.43491400
C	-3.01779600	5.21337100	-0.32533900
H	-3.04976000	6.25800100	-0.64415500
C	-3.62656100	4.22849400	-1.09688200
H	-4.13372000	4.51302000	-2.02122100
C	-3.59395100	2.88783300	-0.70829100
C	-1.61157900	3.22451400	2.59595600
H	-1.71849000	2.14661000	2.79188700
C	-2.20550100	4.00043300	3.77466300
H	-3.29029100	3.85713000	3.88707100
H	-2.02997300	5.08275200	3.66780300
H	-1.72585000	3.68353600	4.71348000
C	-0.12066400	3.52191100	2.48751600
H	0.38556800	3.26883000	3.43172800
H	0.06114800	4.58723000	2.28201500
H	0.34495300	2.91950400	1.69616200
C	-4.26953500	1.82484200	-1.55740200
H	-3.74106700	0.88452800	-1.34770900
C	-5.74080600	1.61847300	-1.19503400
H	-6.20296000	0.87202400	-1.86157500
H	-6.30947700	2.55745600	-1.29169700
H	-5.85810700	1.25119800	-0.16709000
C	-4.13388700	2.11028600	-3.05217600
H	-4.47497700	1.24292600	-3.64483400
H	-3.09570900	2.37056200	-3.32117500
H	-4.75735400	2.95699600	-3.37849600
C	-2.42306500	-3.02241400	0.56451900
C	-1.58525900	-3.84880200	1.34142900
C	-1.38133800	-5.16724600	0.91579600
H	-0.73023200	-5.81732600	1.50493300
C	-1.99660000	-5.66720800	-0.22314000
H	-1.83726900	-6.70567200	-0.52351400
C	-2.80329200	-4.83361200	-0.99189300
H	-3.26997600	-5.22897000	-1.89670000
C	-3.01587200	-3.50298200	-0.62685700
C	-0.91644400	-3.38214000	2.62095000
H	-1.19413600	-2.32935500	2.78190100
C	-1.35252200	-4.20021000	3.83931100
H	-2.44212000	-4.21324500	3.98773300

H	-0.89596100	-3.78722300	4.75192600
H	-1.02297200	-5.24804800	3.75358100
C	0.60128000	-3.43570400	2.49002600
H	1.07559800	-3.03956000	3.40049000
H	0.94660600	-2.81992000	1.64944600
H	0.95430800	-4.46777200	2.34648000
C	-3.89161100	-2.60127700	-1.48036600
H	-3.54652800	-1.57496000	-1.29322700
C	-5.37159900	-2.66772900	-1.10094600
H	-5.75376900	-3.69908600	-1.16970800
H	-5.97273200	-2.03797400	-1.77696300
H	-5.54653400	-2.30453100	-0.07982000
C	-3.72276000	-2.88179300	-2.97283300
H	-4.18754700	-3.83195900	-3.27851200
H	-2.65755900	-2.95759000	-3.25093100
H	-4.21749200	-2.09790800	-3.57279700
C	3.86978800	1.64115900	-1.64136400
C	4.35651500	0.42909700	-2.16107800
H	5.16474300	0.51598200	-2.88522800
C	4.10320100	-0.86725400	-1.68230400
C	4.58731100	2.89314400	-2.07038900
H	4.92814300	3.46686700	-1.19642800
H	3.91102300	3.55563000	-2.62901000
H	5.45099800	2.65316200	-2.70150700
C	5.05341900	-1.94719300	-2.12340600
H	5.60034900	-2.35052300	-1.25832300
H	5.77711100	-1.56113600	-2.85078900
H	4.51140700	-2.79498500	-2.56441100
C	2.58053500	2.99362800	-0.19990900
C	3.10241400	3.28098400	1.07984300
C	2.94661900	4.57356200	1.58563400
H	3.35877600	4.81891600	2.56576900
C	2.25730500	5.54905800	0.87314200
H	2.14649900	6.55436600	1.28710200
C	1.68002100	5.22844200	-0.34827400
H	1.10155800	5.98286600	-0.88686000
C	1.82995200	3.95511500	-0.90636100
C	3.81811400	2.21086500	1.88161400
H	3.33959100	1.25587500	1.61117600
C	5.29986400	2.11346800	1.51545400
H	5.80043900	1.35162800	2.13346200
H	5.81081000	3.07498000	1.68702100
H	5.44658300	1.82996200	0.46370100
C	3.64132300	2.38619200	3.38547600
H	2.57697400	2.45585500	3.65160900
H	4.15742300	3.28276200	3.76445900
H	4.06473300	1.52263700	3.91802300
C	1.15096500	3.62841200	-2.22050500
H	1.56383200	2.67484600	-2.58489000
C	1.38189000	4.67996300	-3.30340100
H	0.95763100	4.33893800	-4.26080800
H	2.45202900	4.88328000	-3.46297100
H	0.89707300	5.63726300	-3.05475300
C	-0.34101000	3.41044000	-1.98654700
H	-0.51718800	2.63807500	-1.22419400
H	-0.83356500	3.08921900	-2.91871900
H	-0.82736700	4.33365500	-1.64019100
C	3.09243400	-2.47737300	-0.28140700
C	3.76081100	-2.71651800	0.93716600
C	3.78949500	-4.01892500	1.44258800
H	4.31093600	-4.21391100	2.38323200
C	3.16232000	-5.06479600	0.77724900
H	3.19355500	-6.07653100	1.18912900
C	2.48151800	-4.81195700	-0.40726600
H	1.97347300	-5.63058800	-0.92300800
C	2.44054900	-3.52892200	-0.96002500
C	4.44933000	-1.60505900	1.70281000
H	4.32093300	-0.68122500	1.12070900
C	5.95016800	-1.85839800	1.84279800
H	6.44001400	-1.01263300	2.35027600
H	6.43447000	-1.98699900	0.86272300

H	6.15450700	-2.76354800	2.43683300
C	3.78807500	-1.38863800	3.06277900
H	2.73214400	-1.10083600	2.94320300
H	4.30343900	-0.59076500	3.61876700
H	3.83029400	-2.30141100	3.67871000
C	1.69994100	-3.30256500	-2.26136900
H	1.92340600	-2.27861900	-2.59851500
C	2.13609600	-4.26007100	-3.36892600
H	1.86193300	-5.30219900	-3.14034900
H	3.22411400	-4.23286800	-3.53343700
H	1.64629200	-3.99250700	-4.31856900
C	0.19459500	-3.38344600	-2.03578700
H	-0.10889000	-4.39074100	-1.71599600
H	-0.34422900	-3.14263400	-2.96691500
H	-0.13244000	-2.67805700	-1.25903600
N	-1.84470700	-0.20581600	-3.64935700
H	-1.22936200	-0.14996300	-4.46806300
H	-2.53785100	0.55561400	-3.66280500
H	-2.35858800	-1.09802100	-3.64023000

Cartesian coordinates of the optimized geometry of Int-**8** at PBE0-D3BJ/def2-SVP level of theory:

Ga	-1.92499900	-0.12833300	-0.45349700
Ga	1.89351700	0.13007500	-0.02815000
P	0.06460900	0.30227500	-1.35389600
P	0.44070400	0.04707100	3.07820500
O	-1.87551700	-0.33541000	1.51655300
C	-0.86201600	-0.17106400	2.20453300
N	-3.00654500	-1.70661600	-1.00531000
N	-3.42126200	1.15669300	-0.73713800
N	3.12014100	-1.14506800	0.94040400
N	2.89739600	1.73151900	0.66224800
C	-4.11191100	-1.59843200	-1.74714700
C	-4.76307000	-0.39096600	-2.01752200
H	-5.65183700	-0.45993200	-2.64124300
C	-4.49437900	0.87201000	-1.47458900
C	-4.74917100	-2.83772700	-2.31523500
H	-5.62639600	-2.57907500	-2.91927900
H	-5.05570300	-3.52878600	-1.51810900
H	-4.03487000	-3.39382000	-2.93604800
C	-5.52353500	1.93780800	-1.73452600
H	-6.33105900	1.55087900	-2.36649400
H	-5.07813200	2.81816800	-2.21369200
H	-5.95142000	2.29751300	-0.78836100
C	-2.63115600	-3.03842200	-0.64278800
C	-1.77508200	-3.79571400	-1.46892600
C	-1.59325200	-5.14825700	-1.16039100
H	-0.94714800	-5.75647700	-1.79863200
C	-2.21114100	-5.73002400	-0.05903100
H	-2.06168400	-6.79067800	0.15721200
C	-2.99886200	-4.95057400	0.78162600
H	-3.45800600	-5.40299000	1.66430500
C	-3.22003900	-3.59724800	0.51325000
C	-1.04611100	-3.19981400	-2.66050300
H	-1.27849900	-2.12432900	-2.69206600
C	-1.47099200	-3.83024000	-3.98729100
H	-2.54773000	-3.71787600	-4.18112900
H	-1.23960900	-4.90733300	-4.01463000
H	-0.93490800	-3.35496500	-4.82345900
C	0.46755300	-3.31199000	-2.48317400
H	0.98033900	-2.82333600	-3.32698400
H	0.80188000	-4.36085500	-2.44579400
H	0.78857600	-2.81104600	-1.56019600
C	-4.10362700	-2.77677700	1.43912900
H	-4.04615600	-1.73226200	1.10409000
C	-5.56955900	-3.20636300	1.35962300
H	-6.18720500	-2.58665700	2.02864900
H	-5.69666300	-4.25750000	1.66532800
H	-5.97574300	-3.10056400	0.34334200

C	-3.60928800	-2.80947900	2.88381100
H	-4.23875200	-2.16096400	3.51301800
H	-2.57719500	-2.44331000	2.96041400
H	-3.65089800	-3.82489700	3.30938200
C	-3.34627100	2.48890500	-0.21827400
C	-2.82390800	3.52606400	-1.02124700
C	-2.92276200	4.83685100	-0.54423500
H	-2.53706400	5.65525400	-1.15745000
C	-3.49133800	5.11683100	0.69345800
H	-3.56330800	6.14962600	1.04307700
C	-3.94482400	4.07580500	1.49529700
H	-4.36569900	4.29609100	2.47951800
C	-3.88027000	2.74840900	1.06154900
C	-2.15960800	3.27367200	-2.36466500
H	-2.14173700	2.18577300	-2.52928900
C	-2.91239100	3.92394400	-3.52724200
H	-3.94730400	3.56401700	-3.62073100
H	-2.40119200	3.70575800	-4.47767300
H	-2.94975100	5.01973300	-3.41704900
C	-0.70692800	3.74537500	-2.35116900
H	-0.22302300	3.50911400	-3.31161900
H	-0.13402800	3.23632800	-1.56499000
H	-0.63567600	4.83364900	-2.19717000
C	-4.40906700	1.63787700	1.95311500
H	-4.14337000	0.68352200	1.47926100
C	-5.93266000	1.68330400	2.07995200
H	-6.26908400	2.62982000	2.53308800
H	-6.28918200	0.86076600	2.72007300
H	-6.43142300	1.58283900	1.10492700
C	-3.75337700	1.64986200	3.33261500
H	-4.01949800	2.55425300	3.90314500
H	-2.65839500	1.59907800	3.26217400
H	-4.08791200	0.77946500	3.91822500
C	3.74960000	-0.80291500	2.06634200
C	3.94744800	0.51890200	2.47095700
H	4.49034100	0.65493500	3.40482400
C	3.64356600	1.69205000	1.76475400
C	4.28039400	-1.86894700	2.98476700
H	4.57245700	-2.78185100	2.45171300
H	3.47754000	-2.14357900	3.68887700
H	5.12932500	-1.49867200	3.57258300
C	4.21439800	2.96448900	2.32825000
H	4.55959800	3.64070500	1.53562900
H	5.04450600	2.74428200	3.00987000
H	3.44249100	3.50828800	2.89234000
C	3.28752800	-2.46589100	0.42578700
C	4.34198600	-2.70315900	-0.49088900
C	4.43467700	-3.96540100	-1.08439000
H	5.23311700	-4.16524600	-1.80220100
C	3.53565200	-4.97841400	-0.76251500
H	3.61650000	-5.95483000	-1.24607800
C	2.55758600	-4.75256600	0.19697400
H	1.87573200	-5.55908900	0.47666000
C	2.42720800	-3.50716600	0.82041700
C	5.40860700	-1.65179200	-0.75381500
H	4.95660100	-0.67173600	-0.53733800
C	6.58227700	-1.81600400	0.21567100
H	7.34933300	-1.04849300	0.02647800
H	7.05140700	-2.80542900	0.09489700
H	6.26517100	-1.71918000	1.26174600
C	5.92959200	-1.64455600	-2.19043700
H	5.12122000	-1.63681900	-2.93716700
H	6.55447900	-2.52655700	-2.40001600
H	6.56156400	-0.75900200	-2.36130800
C	1.40873800	-3.30712000	1.91817200
H	1.60852900	-2.33428100	2.38827400
C	1.51125700	-4.37981800	3.00032700
H	0.85020700	-4.12232400	3.84212300
H	2.53709500	-4.47835200	3.38812000
H	1.19735300	-5.36841500	2.62899900
C	0.00066900	-3.22362900	1.35886700

H	-0.08268000	-2.40576200	0.62967700
H	-0.71702800	-3.02819500	2.16592700
H	-0.28701500	-4.15800000	0.85923100
C	2.65265600	3.00995400	0.06620300
C	3.39149200	3.39407800	-1.07170800
C	3.13991400	4.64254700	-1.64770400
H	3.70678300	4.95114000	-2.52947400
C	2.19384100	5.50332000	-1.10588600
H	2.00539100	6.47450100	-1.56951900
C	1.49402300	5.12418200	0.03264000
H	0.75200500	5.80251600	0.45977600
C	1.70778700	3.88572900	0.64330000
C	4.50273300	2.52606800	-1.62763800
H	4.40735100	1.54448100	-1.14188100
C	5.87962500	3.07125700	-1.24490100
H	6.68000500	2.41789200	-1.62847700
H	5.99620500	3.13918800	-0.15354300
H	6.03635800	4.07880500	-1.66183200
C	4.39269900	2.34002600	-3.14060600
H	3.38090000	2.02625000	-3.43842300
H	5.11771900	1.59075500	-3.49987400
H	4.61213700	3.27511900	-3.67860200
C	0.91542700	3.52589300	1.88278800
H	1.35075400	2.61653600	2.32248400
C	0.94611300	4.62419200	2.94516300
H	0.38164400	5.51434700	2.62554100
H	1.96938200	4.95312400	3.18607500
H	0.47775400	4.25859200	3.87174400
C	-0.51964800	3.18141000	1.51507400
H	-1.03505000	4.05099700	1.08512500
H	-1.07375000	2.85608600	2.40591500
H	-0.54643100	2.36843400	0.77588200
H	3.74758100	0.04725700	-2.29576100
H	2.10237300	-0.11143800	-2.56763900
N	2.88142900	-0.35526600	-1.94607800
H	2.96901200	-1.37277500	-1.96922300

Cartesian coordinates of the optimized geometry of TS-**7** at PBE0-D3BJ/def2-SVP level of theory:

Ga	1.58933700	-0.09967400	-0.60758800
Ga	-1.62468500	0.11554900	0.54071600
P	-0.47970900	0.04846700	-1.39385300
P	-0.73756300	0.08760200	2.80919300
O	1.70736200	-0.13410800	1.43638300
C	0.83357100	-0.05551800	2.29544200
N	3.03021900	1.25847700	-0.95971100
N	2.82010400	-1.64001900	-0.99299800
N	-2.90320100	1.65732300	0.78543600
N	-3.09391200	-1.23851900	0.84394000
C	4.06288200	0.99717700	-1.76315100
C	4.41430100	-0.28563600	-2.20108400
H	5.26406800	-0.33912700	-2.87866300
C	3.87840700	-1.51383800	-1.79551500
C	4.93282600	2.12194300	-2.25623800
H	5.84166800	1.72986400	-2.72739800
H	5.20996000	2.81316500	-1.45008200
H	4.38476300	2.71806700	-2.99879400
C	4.57323900	-2.74261200	-2.31739800
H	5.50602500	-2.47333100	-2.82632000
H	3.92256000	-3.26600800	-3.03083700
H	4.79134000	-3.46216400	-1.51762200
C	2.89633800	2.61318500	-0.51776100
C	2.23334000	3.56835600	-1.31512200
C	2.24033700	4.90210600	-0.88933200
H	1.72682100	5.65302000	-1.49426900
C	2.89220700	5.28880100	0.27260700
H	2.89909800	6.33845100	0.57613500
C	3.51968100	4.33023400	1.06220800
H	4.01150300	4.64056500	1.98586500

C	3.52220800	2.98279200	0.69579200
C	1.52787500	3.22210500	-2.61317400
H	1.64688100	2.14153200	-2.78605900
C	2.10528400	3.97622600	-3.81376200
H	3.18731100	3.82619700	-3.94225000
H	1.93517400	5.06078000	-3.72241700
H	1.61021700	3.64465100	-4.73940700
C	0.03342900	3.50312600	-2.50342100
H	-0.47606000	3.21131200	-3.43458000
H	-0.16217500	4.57218300	-2.33236500
H	-0.41868300	2.92255800	-1.68856000
C	4.21093300	1.94847600	1.57177800
H	3.66808800	1.00468300	1.42134600
C	5.67016800	1.70674700	1.17966400
H	6.14249900	0.99893200	1.87987300
H	6.24858300	2.64438900	1.20659200
H	5.76168700	1.27470100	0.17465000
C	4.12540900	2.29730900	3.05574900
H	4.49043500	1.45470500	3.66670700
H	3.09455100	2.54746900	3.35295200
H	4.75721500	3.15968200	3.31931700
C	2.50203900	-2.96871700	-0.56788400
C	1.68720200	-3.80422700	-1.35870200
C	1.51668000	-5.13323500	-0.95179100
H	0.88505500	-5.79272200	-1.55155700
C	2.14028600	-5.62986000	0.18376800
H	2.00783500	-6.67585400	0.47076500
C	2.91727700	-4.78402200	0.96965600
H	3.38325300	-5.17698100	1.87521600
C	3.09832200	-3.44340400	0.62404100
C	1.00205400	-3.33632200	-2.62967100
H	1.24213900	-2.27166800	-2.77127500
C	1.46441100	-4.11179400	-3.86581200
H	2.55106300	-4.06389900	-4.02823800
H	0.97634700	-3.70825400	-4.76625000
H	1.19096900	-5.17661100	-3.79361500
C	-0.51313400	-3.44419800	-2.49738600
H	-1.00283800	-3.04345300	-3.39765600
H	-0.87715200	-2.86042900	-1.64201900
H	-0.83176600	-4.49040500	-2.37677900
C	3.93444900	-2.52714800	1.50380500
H	3.52653500	-1.51516200	1.37088200
C	5.41001100	-2.48408600	1.10103300
H	5.85203900	-3.49358200	1.10575000
H	5.98161700	-1.86262200	1.80908600
H	5.55344800	-2.04956300	0.10327900
C	3.81424700	-2.88042100	2.98455700
H	4.32871100	-3.82185800	3.23251000
H	2.76158800	-2.99744800	3.28697000
H	4.28966700	-2.10015600	3.60214400
C	-3.90614800	1.55050600	1.65141000
C	-4.35795700	0.32633600	2.17483800
H	-5.16082900	0.39358800	2.90696400
C	-4.07728400	-0.96421900	1.69501700
C	-4.64843500	2.78514800	2.08636600
H	-5.00388600	3.35428900	1.21529000
H	-3.98360200	3.45936900	2.64497700
H	-5.50455700	2.52515200	2.71977900
C	-4.99140200	-2.06937000	2.14794700
H	-5.52651600	-2.49923000	1.28828900
H	-5.72524800	-1.70014400	2.87381000
H	-4.41983200	-2.89402200	2.59617300
C	-2.65778700	2.93360000	0.19691300
C	-3.19149400	3.20397000	-1.08171900
C	-3.06465300	4.49702500	-1.59455500
H	-3.48530400	4.72875000	-2.57427100
C	-2.39425800	5.49052400	-0.88933500
H	-2.30615600	6.49578600	-1.30877300
C	-1.80765600	5.18873500	0.33222500
H	-1.24412000	5.95781900	0.86564100
C	-1.92721400	3.91511100	0.89684700

C	-3.88912800	2.11747600	-1.87720300
H	-3.38691800	1.17327000	-1.61182200
C	-5.36497200	1.98637600	-1.49772900
H	-5.85437600	1.21476800	-2.11241200
H	-5.89808300	2.93675100	-1.66324000
H	-5.49623400	1.69843800	-0.44516700
C	-3.73133300	2.29779300	-3.38269400
H	-2.67202200	2.39591700	-3.65992000
H	-4.27410100	3.18097400	-3.75549900
H	-4.13800100	1.42447000	-3.91212900
C	-1.23092700	3.60943100	2.20625100
H	-1.62262900	2.65152900	2.58131400
C	-1.46810700	4.66354300	3.28495300
H	-1.02718700	4.33505500	4.23904600
H	-2.53993400	4.84933700	3.45526100
H	-1.00241900	5.62758600	3.02614500
C	0.26133700	3.41323400	1.95564000
H	0.43799000	2.64784200	1.18647300
H	0.77044100	3.09171800	2.87794700
H	0.72976700	4.34469100	1.60646600
C	-3.03926500	-2.54746500	0.27818200
C	-3.70643800	-2.79422600	-0.93920800
C	-3.70797800	-4.09410100	-1.45227000
H	-4.22787000	-4.29507600	-2.39246900
C	-3.05667700	-5.12964200	-0.79442700
H	-3.06708100	-6.13951800	-1.21184200
C	-2.37854200	-4.86863100	0.38979200
H	-1.85053000	-5.67832000	0.89919400
C	-2.36257900	-3.58799700	0.94915400
C	-4.42205300	-1.69535200	-1.69765200
H	-4.30830600	-0.76991800	-1.11508800
C	-5.91848800	-1.97859500	-1.82673100
H	-6.42911800	-1.14380800	-2.33183200
H	-6.39256600	-2.11532300	-0.84277600
H	-6.10869800	-2.88878200	-2.41764600
C	-3.77437600	-1.46504100	-3.06184800
H	-2.72321200	-1.15789700	-2.94938400
H	-4.30826200	-0.67704900	-3.61412400
H	-3.80283600	-2.37788900	-3.67825300
C	-1.61025400	-3.34863400	2.24047300
H	-1.87131000	-2.34105800	2.59792700
C	-1.97902900	-4.33992500	3.34206600
H	-1.66105400	-5.36525600	3.09521700
H	-3.06390000	-4.36527700	3.52807000
H	-1.48248500	-4.06149100	4.28485900
C	-0.10859300	-3.35491700	1.97868500
H	0.23418400	-4.34561200	1.64689000
H	0.44367000	-3.08450300	2.89255100
H	0.16061300	-2.63440900	1.19358900
N	1.93044600	-0.16579900	3.94894100
H	1.29598500	-0.13150500	4.74444600
H	2.58226900	0.61760200	3.98220400
H	2.45510500	-1.04018800	3.95195900

Cartesian coordinates of the optimized geometry of TS-**8** at PBE0-D3BJ/def2-SVP level of theory:

Ga	1.89387300	-0.21272000	0.50855800
Ga	-1.74781700	0.20312900	-0.16795000
P	-0.07957100	0.36105800	1.35387100
P	-0.62944500	0.13231700	-2.86368600
O	1.77448900	-0.41072000	-1.50262100
C	0.73865500	-0.18015400	-2.11189200
N	2.91651200	-1.84626700	0.98653300
N	3.45398500	0.99293100	0.75715200
N	-3.18693100	-0.97198300	-0.93721100
N	-2.73419700	1.87678300	-0.68657300
C	4.03216500	-1.79977800	1.72110100
C	4.73351500	-0.62538800	2.01113200
H	5.62058600	-0.74173700	2.63025000

C	4.51887300	0.65501000	1.48421000
C	4.61825300	-3.07631900	2.25987900
H	5.52417700	-2.87086400	2.84150100
H	4.86383500	-3.77669600	1.44987400
H	3.89145200	-3.59655900	2.89756600
C	5.59369900	1.67300600	1.74761300
H	6.38897100	1.24566300	2.36897500
H	5.18685800	2.56419300	2.24125700
H	6.02918700	2.02730500	0.80285200
C	2.48678600	-3.15173000	0.58825800
C	1.61784000	-3.90467000	1.40388100
C	1.38577400	-5.24031400	1.05774400
H	0.72838500	-5.84567700	1.68697800
C	1.96712700	-5.80818400	-0.07004600
H	1.77879200	-6.85612800	-0.31560500
C	2.76720000	-5.02975600	-0.89990800
H	3.19729000	-5.46998700	-1.80301500
C	3.03793900	-3.69365800	-0.59403400
C	0.92260200	-3.32054900	2.62050900
H	1.21457800	-2.26196300	2.69821300
C	1.30996800	-4.02874600	3.91919100
H	2.39242100	-3.99452400	4.11149400
H	1.00947300	-5.08872100	3.90542000
H	0.80721600	-3.55397900	4.77592900
C	-0.59421500	-3.34559700	2.43673000
H	-1.08623700	-2.88245400	3.30626700
H	-0.97910600	-4.37285400	2.33752300
H	-0.88785200	-2.77871000	1.54344000
C	3.93446900	-2.87373700	-1.50709400
H	3.88748800	-1.83299700	-1.15948800
C	5.39563700	-3.31827500	-1.42501000
H	6.02185700	-2.70232000	-2.08965200
H	5.51224500	-4.36978100	-1.73358400
H	5.79887200	-3.21938300	-0.40693000
C	3.44511300	-2.88487500	-2.95350200
H	4.07118700	-2.22098200	-3.56982000
H	2.40927300	-2.52749800	-3.02611300
H	3.49582600	-3.89178500	-3.39753100
C	3.44194200	2.32220700	0.22503100
C	2.97261900	3.39362100	1.01471400
C	3.14129500	4.69189800	0.52274300
H	2.79712100	5.53621700	1.12524600
C	3.72982500	4.92774300	-0.71451800
H	3.85883500	5.95144100	-1.07422300
C	4.12764900	3.85546700	-1.50486400
H	4.56155100	4.04250700	-2.49027100
C	3.98828800	2.53858000	-1.05777200
C	2.29555400	3.19237400	2.35989900
H	2.22533000	2.10901300	2.54018700
C	3.07785100	3.82080000	3.51499800
H	4.09431400	3.41298300	3.61535700
H	2.55538700	3.64037900	4.46714700
H	3.16800800	4.91187600	3.39036600
C	0.86871700	3.73657400	2.33555000
H	0.36704400	3.53047200	3.29369800
H	0.27446200	3.25531700	1.54738700
H	0.85480100	4.82612700	2.17646000
C	4.44841200	1.38968600	-1.93857300
H	4.12028400	0.45846400	-1.45779800
C	5.97182600	1.33188200	-2.05936200
H	6.37289100	2.25192400	-2.51428800
H	6.27511500	0.48451900	-2.69462500
H	6.45779200	1.20167100	-1.08145400
C	3.79749900	1.43502900	-3.31955700
H	4.12814300	2.31235300	-3.89824400
H	2.70195900	1.46775300	-3.24710400
H	4.06804700	0.53735300	-3.89694000
C	-3.84799900	-0.57934200	-2.03111300
C	-3.96126900	0.75318000	-2.43301200
H	-4.54487100	0.93060600	-3.33495100
C	-3.56019600	1.90119400	-1.72770200

C	-4.50025300	-1.60629900	-2.91668600
H	-4.93307400	-2.44003800	-2.35059800
H	-3.72723300	-2.03124000	-3.57817300
H	-5.27367900	-1.15195900	-3.54756300
C	-4.11443700	3.20898600	-2.22339400
H	-4.37090700	3.88068200	-1.39396600
H	-5.00148000	3.03914000	-2.84524300
H	-3.36261000	3.73235500	-2.83275400
C	-3.44685100	-2.27945800	-0.42422200
C	-4.51330900	-2.44700500	0.49329200
C	-4.71542700	-3.71453400	1.04601000
H	-5.52398600	-3.86424500	1.76419000
C	-3.90780400	-4.79323500	0.69494900
H	-4.07524200	-5.77238700	1.15009800
C	-2.90913100	-4.62523200	-0.25423400
H	-2.29802200	-5.47916800	-0.55574600
C	-2.67615500	-3.37876100	-0.84554800
C	-5.44880700	-1.29954900	0.83823600
H	-4.85938300	-0.37636700	0.74026800
C	-6.61878000	-1.20420900	-0.14493100
H	-7.29840400	-0.38967700	0.15178400
H	-7.19769400	-2.14172400	-0.15879500
H	-6.28632900	-0.99424200	-1.16872100
C	-6.00031200	-1.37110800	2.26168300
H	-5.21969600	-1.57377100	3.00941300
H	-6.76236400	-2.15995900	2.36416200
H	-6.48920000	-0.42048000	2.52564200
C	-1.64314000	-3.23998300	-1.94087600
H	-1.79143800	-2.25863900	-2.41351000
C	-1.80051700	-4.30409900	-3.02474800
H	-1.12498000	-4.08372700	-3.86570200
H	-2.82988900	-4.34533500	-3.41350400
H	-1.54254500	-5.30911900	-2.65486200
C	-0.23248500	-3.23415700	-1.37896300
H	-0.10295300	-2.42923600	-0.64155300
H	0.49978900	-3.07465300	-2.18180100
H	0.00372000	-4.18519900	-0.88323100
C	-2.41589600	3.12011500	-0.05310100
C	-3.05216700	3.44596300	1.16177400
C	-2.77580100	4.68264300	1.75049500
H	-3.26439700	4.95123900	2.68935000
C	-1.89727200	5.57991800	1.15505400
H	-1.69246600	6.54309200	1.62856100
C	-1.27434100	5.24247600	-0.03958400
H	-0.57227400	5.94268100	-0.49765200
C	-1.51602000	4.01679300	-0.66600700
C	-4.06789300	2.50831900	1.78039100
H	-3.83822500	1.50334200	1.40247900
C	-5.48812500	2.84982100	1.32729900
H	-6.21726900	2.15259800	1.77114700
H	-5.59038700	2.78329600	0.23445900
H	-5.76760200	3.87163400	1.63113700
C	-3.97239800	2.45166500	3.30158600
H	-2.94552900	2.22640700	3.62698900
H	-4.64418400	1.67378200	3.70019500
H	-4.27659900	3.39883400	3.77424400
C	-0.78192700	3.67789200	-1.94634500
H	-1.26684000	2.80033800	-2.39900500
C	-0.80606500	4.80892200	-2.97253400
H	-0.20770200	5.67232200	-2.64169000
H	-1.82598600	5.17278600	-3.17387400
H	-0.37369400	4.46174600	-3.92347700
C	0.64963100	3.26583300	-1.63107700
H	1.21393100	4.10454400	-1.20061700
H	1.16525200	2.93461500	-2.54365900
H	0.66692700	2.44066300	-0.90505200
H	-3.58965200	-0.19358300	2.81169300
H	-1.94113600	-0.28507500	2.63393300
N	-2.84050900	-0.50548500	2.19757700
H	-2.89435100	-1.52160300	2.14381300

5. References

[1] M. K. Sharma, C. Wölper, G. Haberhauer, S. Schulz, "Multi-Talented Gallaphosphene for Ga−P−Ga Heteroallyl Cation Generation, CO_2 Storage, and C(sp^3)−H Bond Activation" *Angew. Chem. Int. Ed.* **2021**, *60*, 6784–6790; *Angew. Chem.* **2021**, *133*, 6859–6865.

[2] G. R. Fulmer, A. J. M. Miller, N. H. Sherden, H. E. Gottlieb, A. Nudelman, B. M. Stoltz, J. E. Bercaw and K. I. Goldberg, "NMR Chemical Shifts of Trace Impurities: Common Laboratory Solvents, Organics, and Gases in Deuterated Solvents Relevant to the Organometallic Chemist" *Organometallics* **2010**, *29*, 2176–2179.

[3] M. K. Sharma, P. Dhawan, C. Helling, C. Wölper, S. Schulz, "Bis-Phosphaketenes $LM(PCO)_2$ (M = Ga, In): A New Class of Reactive Group 13 Metal-Phosphorus Compounds" *Chem. Eur. J.* **2022**, e202200444.

[4] G. M. Sheldrick, "Phase annealing in SHELX-90: direct methods for larger structures" *Acta Crystallogr., Sect. A*. **1990**, *A46*, 467−473.

[5] G. M. Sheldrick, SHELXL-2017, "Program for the Refinement of Crystal Structures" University of Göttingen, Göttingen (Germany) **2017**. See also: G. M. Sheldrick "Crystal structure refinement with SHELXL" *Acta Cryst.* **2015**, *C71*, 3-8.

[6] C. B. Hübschle, G. M. Sheldrick, B. Dittrich, "shelXle, A Qt GUI for SHELXL" *J. Appl. Crystallogr.* 2011, **44**, 1281−1284.

[7] M. J. Frisch, G. W. Trucks, H. B. Schlegel, G. E. Scuseria, M. A. Robb, J. R. Cheeseman, G. Scalmani, V. Barone, G. A. Petersson, H. Nakatsuji, X. Li, M. Caricato, A. V. Marenich, J. Bloino, B. G. Janesko, R. Gomperts, B. Mennucci, H. P. Hratchian, J. V. Ortiz, A. F. Izmaylov, J. L. Sonnenberg, D. Williams-Young, F. Ding, F. Lipparini, F. Egidi, J. Goings, B. Peng, A. Petrone, T. Henderson, D. Ranasinghe, V. G. Zakrzewski, J. Gao, N. Rega, G. Zheng, W. Liang, M. Hada, M. Ehara, K. Toyota, R. Fukuda, J. Hasegawa, M. Ishida, T. Nakajima, Y. Honda, O. Kitao, H. Nakai, T. Vreven, K. Throssell, J. A. Montgomery, Jr.; , J. E. Peralta, F. Ogliaro, M. J. Bearpark, J. J. Heyd, E. N. Brothers, K. N. Kudin, V. N. Staroverov, T. A. Keith, R. Kobayashi, J. Normand, K. Raghavachari, A. P. Rendell, J. C. Burant, S. S. Iyengar, J. Tomasi, M. Cossi, J. M. Millam, M. Klene, C. Adamo, R. Cammi, J. W. Ochterski, R. L. Martin, K. Morokuma, O. Farkas, J. B. Foresman, D. J. Fox, Gaussian, Inc., Wallingford CT, **2016**.

[8] C. Adamo, V. Barone, "Toward reliable density functional methods without adjustable parameters: The PBE0 model" *J. Chem. Phys.* **1999**, *110*, 6158-6170.

[9] S. Grimme, S. Ehrlich, L. Goerigk, "Effect of the damping function in dispersion corrected density functional theory" *J. Comp. Chem.* **2011**, *32*, 1456-1465.

[10] F. Weigend, R. Ahlrichs, "Balanced basis sets of split valence, triple zeta valence and quadruple zeta valence quality for H to Rn: Design and assessment of accuracy" *Phys. Chem. Chem. Phys.* **2005**, *7*, 3297-3305.

[11] F. Weigend, "Accurate Coulomb-fitting basis sets for H to Rn" *Phys. Chem. Chem. Phys.* **2006**, *8*, 1057-1065.

[12] A. V. Marenich, C. J. Cramer, D. G. Truhlar, "Universal Solvation Model Based on Solute Electron Density and on a Continuum Model of the Solvent Defined by the Bulk Dielectric Constant and Atomic Surface Tensions" *J. Phys. Chem. B* **2009**, *113*, 6378-6396.

[13] M. Mammen, E. I. Shakhnovich, J. M. Deutch, G. M. Whitesides, "Estimating the Entropic Cost of Self-Assembly of Multiparticle Hydrogen-Bonded Aggregates Based on the Cyanuric Acid·Melamine Lattice" *J. Org. Chem.* **1998**, *63*, 3821-3830.

[14] M. Besora, P. Vidossich, A. Lledós, G. Ujaque, F. Maseras, "Calculation of Reaction Free Energies in Solution: A Comparison of Current Approaches" *J. Phys. Chem. A* **2018**, *122*, 1392-1399.

[15] Y. Liang, S. Liu, Y. Xia, Y. Li, Z.-X. Yu, "Mechanism, Regioselectivity, and the Kinetics of Phosphine-Catalyzed [3+2] Cycloaddition Reactions of Allenoates and Electron-Deficient Alkenes" *Chem. Eur. J.* **2008**, *14*, 4361-4373.

[16] Z.-X. Yu, K. N. Houk, "The intramolecular nitrene type 1,1-cycloaddition reaction of allyl-substituted diazomethanes" *J. Am. Chem. Soc.* **2003**, *125*, 13825-13830.

[17] R. E. Plata, D. A. Singleton, "A Case Study of the Mechanism of Alcohol-Mediated Morita Baylis–Hillman Reactions. The Importance of Experimental Observations" *J. Am. Chem. Soc.* **2015**, *137*, 3811-3826.
